# Influenza A virus exploits transferrin receptor recycling to enter host cells

**DOI:** 10.1073/pnas.2214936120

**Published:** 2023-05-16

**Authors:** Beryl Mazel-Sanchez, Chengyue Niu, Nathalia Williams, Michael Bachmann, Hélèna Choltus, Filo Silva, Véronique Serre-Beinier, Wolfram Karenovics, Justyna Iwaszkiewicz, Vincent Zoete, Laurent Kaiser, Oliver Hartley, Bernhard Wehrle-Haller, Mirco Schmolke

**Affiliations:** ^a^Department of Microbiology and Molecular Medicine, Faculty of Medicine, University of Geneva, 1211 Geneva, Switzerland; ^b^Department of Cell Physiology and Metabolism, Faculty of Medicine, University of Geneva, 1211 Geneva, Switzerland; ^c^Thoracic Surgery, Geneva University Hospitals, 1205 Geneva, Switzerland; ^d^Molecular Modeling Group, Swiss Institute of Bioinformatics, 1015 Lausanne, Switzerland; ^e^Computer-Aided Molecular Engineering Group, Department of Oncology (University of Lausanne and the Lausanne University Hospital), Ludwig Institute for Cancer Research Lausanne, 1066 Épalinges, Switzerland; ^f^Department of Medicine, Faculty of Medicine, University of Geneva, 1211 Geneva, Switzerland; ^g^Geneva Centre for Emerging Viral Diseases, Geneva University Hospitals, 1205 Geneva, Switzerland; ^h^Division of Infectious Diseases, Geneva University Hospitals, 1205 Geneva, Switzerland; ^i^Department of Pathology and Immunology, Faculty of Medicine, University of Geneva, 1211 Geneva, Switzerland; ^j^Geneva Center of Inflammation Research, University of Geneva, 1211 Geneva, Switzerland

**Keywords:** influenza A virus, transferrin receptor 1, endocytosis, recycling, antiviral

## Abstract

Influenza A virus (IAV) enters host cells mostly through clathrin-dependent receptor-mediated endocytosis. A single bona fide entry receptor protein supporting this entry mechanism remains elusive. Here we performed proximity ligation of biotin to host cell surface proteins in the vicinity of attached trimeric hemagglutinin-HRP and characterized biotinylated targets using mass spectrometry. This approach identified transferrin receptor 1 (TfR1) as a candidate entry protein. Genetic gain-of-function and loss-of-function experiments, as well as in vitro and in vivo chemical inhibition, confirmed the functional involvement of TfR1 in IAV entry. Recycling deficient mutants of TfR1 do not support entry, indicating that TfR1 recycling is essential for this function. The binding of virions to TfR1 via sialic acids confirmed its role as a directly acting entry factor, but unexpectedly even headless TfR1 promoted IAV particle uptake in *trans*. TIRF microscopy localized the entering virus-like particles in the vicinity of TfR1. Our data identify TfR1 recycling as a revolving door mechanism exploited by IAV to enter host cells.

## Significance

We still have an incomplete understanding of the proteins influenza A virus (IAV) uses to enter the host cell. Here we demonstrate that IAV entry is diminished in the absence of transferrin receptor 1 (TfR1). Consequently, pharmacological targeting of TfR1 efficiently interferes with IAV replication. While this process could be mediated by an interaction of IAV HA with glycosylated TfR1, a “headless” TfR1 mutant still functions as a host entry factor for IAV in trans. Our “revolving door” model provides a plausible explanation as to why IAV is capable of entering such a broad range of cell types in culture and why the adaptation of the zoonotic avian IAV to mammalian cells involves primarily an adjustment to attachment factors rather than the entry machinery.

By definition, viral infection of a host cell begins with attachment and entry of the virion. The attachment is frequently mediated by low affinity binding to broadly displayed cell surface structures, such as carbohydrates, lipids, or proteins (1). The subsequent entry step often depends on specific protein–protein interactions between viral ligands and host receptors, which allows selected infection. Virion entry can occur at the cell surface, e.g., by direct fusion of the viral and the plasma membrane or by receptor-medicated endocytosis (reviewed in ref. 2). The latter usually requires the direct interaction of viral surface protein(s) with one or more host surface proteins causing outside-in-signaling events that trigger endocytosis of the virion. The expression pattern of these specific entry receptors defines, at least to some extent, the tropism and transmission routes of probably all viruses.

It is well established that influenza A virus (IAV) binds via the globular head of its major surface glycoprotein hemagglutinin (HA) to sialic acids on the host cell surface (3). These terminal ends of glycans on post-translationally modified host surface proteins are present on all cell types. Accordingly, IAV attaches to and enters into cell lines of various tissue origins in vitro (including keratinocytes, cardiomyocytes, neuronal cells, kidney epithelial cells) (4–9), and removal of sialic acids by enzymatic or genetic means largely abolishes attachment (10–12) and infection. In a second step, IAV virions are endocytosed mostly in a clathrin-dependent fashion, via de novo formation of clathrin-coated pits (13), although clathrin-independent entry, e.g., by micropinocytosis (14) has been reported. After lowering of endosomal pH to 5.0 to 5.5 (15), HA undergoes conformational changes, releasing the N-terminal fusion peptide of the HA2 subunit, thus triggering the fusion of viral and endosomal membranes. Ultimately, this leads to uncoating and release of viral genetic material into the cytoplasm (16).

A number of experimental findings suggest the existence of specific host protein entry factors downstream of the sialic acid-dependent attachment process. Firstly, sialic acids are added to a structurally and functionally diverse population of proteins. It is difficult to invisage how interaction with all these proteins will promote virion uptake. Secondly, the initial attachment environment of the virion on the host cell surface is not necessarily ideal for entry, since virions move laterally on the cell surface after attachment until they reach a suitable point of entry. In this context, the interplay between HA-dependent sialic acid binding and viral neuraminidase (NA)-dependent sialic acid cleavage allows a movement directed by the binding affinity of HA to the nearest host surface proteins (17, 18). Accordingly, application of NA inhibitors causes an attenuated IAV entry phenotype in stratified airway epithelial culture (19) or transformed cells (20). Thirdly, a handful of surface proteins were already identified as potential entry factors of IAV. These included epithelial growth factor receptor (EGFR) (21), free fatty acid receptor 2 (FFAR2) (22), nucleolin (23), and the voltage-dependent calcium channel Ca_v_1.2 (24). Residual viral entry in knockdown/knockout cells suggests at least a level of redundancy among these proposed entry factors and the potential presence of unkown ones.

Here we used a specifically designed molecular probe, consisting of a trimeric IAV HA ectodomain C-terminally fused to horseradish peroxidase (HRP), to identify host surface proteins in the immediate vicinity of its attachment point by biotin proximity ligation. Using multiple gain- and loss-of-function approaches, we identified transferrin receptor 1 (TfR1) as an IAV entry factor. The use of endocytosis-dead mutants of TfR1 confirmed that TfR1 recycling is involved in early viral replication by supporting cellular uptake of IAV virions.

## Results

### Cell Surface Proximity Ligation (CSPL) Coupled to Mass Spectrometry Localizes TfR1 in the Vicinity of Cell Surface-Attached HA Trimers.

The protein environment of attaching/entering IAV particles on the surface of susceptible cells is not well-characterized. In order to identify proteins involved at this step of the viral replication cycle, we expressed a trimeric molecular probe, namely the HA of influenza A/California/04/2009 (H1N1) C-terminally fused to HRP in insect cells (*SI Appendix*, Fig. S1 *A* and *B*). HRP processes biotin phenol into a short-lived biotin radical that will then be covalently linked to proteins in its vicinity (25). Conveniently, this biotin radical does not cross lipid membranes, limiting the biotinylation reaction to largely the cell surface. Besides the HA wt HRP, we also generated a trimeric HA Y98F HRP mutant, known to have a lower affinity for sialic acids (26). This mutant probe allowed us to control for host proteins that were proximity labeled as a consequence of sialic acid-independent interactions. Finally, we generated trimeric HRP probes lacking the HA ectodomain to control for unspecific labelling of host cell proteins. All molecular probes displayed comparable HRP activity, as measured in vitro by processing of a TMB substrate, with a slight reduction measured for the HA wt-HRP probe (*SI Appendix*, Fig. S1*C*). When added to A549 lung epithelial cells, these probes successfully coupled biotin to surface host proteins as confirmed by confocal imaging using Alexa 488-tagged streptavidin (*SI Appendix*, Fig. S2*A*) and by western blots of total cell lysates probed with streptavidin-HRP (*SI Appendix*, Fig. S2*B*). Cells treated with trimeric HA (either wt or Y98F) displayed overall higher protein biotinylation, indicating an HA mediated targeting of HRP to the cell surface. From total A549 cell lysates, we precipitated biotinylated proteins using streptavidin-agarose beads, performed an on-bead digestion with trypsin and analyzed the derived peptides by mass spectrometry. We bioinformatically excluded nonsurface proteins by filtering against a mass spectrometry-based cell surface protein atlas (27) and included only high-confidence cell surface proteins. Only hits with more than five peptides in the HA-wt-HRP approach were included. Next we limited the hits to those proteins which were either absent in the HRP control (0 peptides) or were more than 1.5-fold overrepresented in the HA-wt-HRP versus the HRP control. Proteins commonly found in proximity biotinylation experiments in human cell lines were removed by filtering against the CRAPome database (28) (using an arbitrary cutoff of proteins found in less than 100 from 716 annotated experiments) (Datasets S1–S7). Overall, we did not find substantial differences between the samples from HA wt HRP and HA Y98F HRP-treated cells (*SI Appendix*, Fig. S3*A*), suggesting that the sialic acid-specific interactions do not substantially contribute to the targeting of the probes.

From two independent CSPL experiments, we identified 31 target proteins fulfilling the above criteria (intersection of red and blue circles in *SI Appendix*, Fig. S3 *B* and *C* and Dataset S7). In combination with hits obtained from a smaller-scale pre-experiment (three combined wells of a 24-well plate instead of a 6-well format), but with the same cell to HA-probe ratio (yellow circle in *SI Appendix*, Fig. S3*B*), we further narrowed the hits down to one main candidate: transferrin receptor protein 1 (TfR1). STRING-analysis (https://string-db.org) of all proteins found in at least two of the mass spectrometry runs (*SI Appendix*, Fig. S4*A*) revealed an enrichment for the GO-molecular function “Virus receptor activity” (red) and the GO-biological process “entry into host cell” (blue), both indicated with the lowest false discovery rate (*SI Appendix*, Fig. S4*B*). In contrast, proteins enriched in only one of the mass spectrometry runs displayed enrichment for unrelated biological processes, and substantially higher false discovery rates within the two GO clusters (*SI Appendix*, Fig. S4*C*).

High abundance of a host protein on the cell surface might increase the chance of random biotinylation events. We thus searched for TfR1 in the published surface proteome database and found that it is not listed as a highly abundant surface protein in A549 cells (29), making unspecific biotinylation less likely.

### Genetic Depletion of TfR1 Impairs Early Viral Replication Steps.

**Fig. 1.**
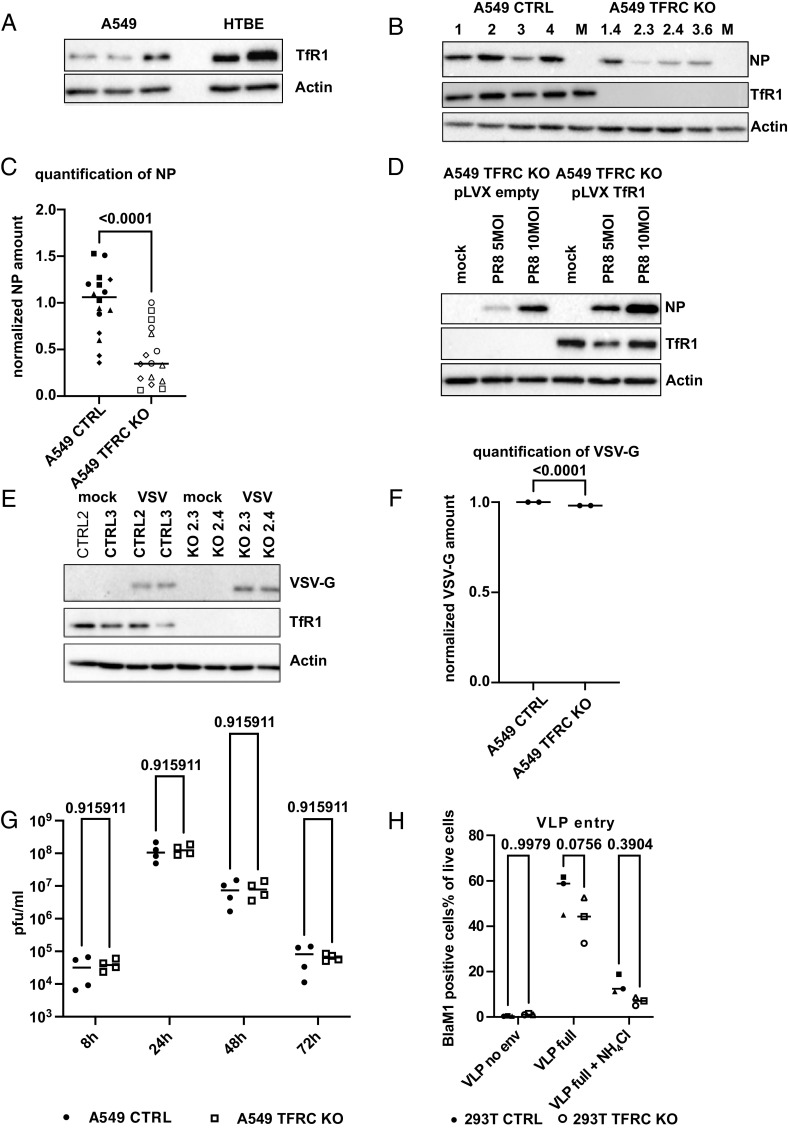
TFRC deficiency hampers IAV entry. (*A*) TfR1 levels in A549 (n = 3) and primary human tracheobronchial epithelial cells (HTBE, n = 2) as determined by western blot. Beta actin served as a loading control. (*B*) Single A549 cell clones transfected with a Cas9 expression plasmid only (1 to 4) or a Cas9 expression plasmid containing the cds for a TFRC targeting guide RNA (1.4, 2.3, 2.4 and 3.6) were infected for 4 h with 10 multiplicity of infection (MOI) of PR8. Total cell lysates were separated by SDS-PAGE and analyzed by western blot for NP and TfR1 levels. Beta actin served as a loading control. A representative blot from four independent experiments is shown. (*C*) Quantification of four independent infection experiments as shown in *B*. Each symbol refers to one independent experiment performed with one A549 CTRL and one A549 TFRC KO clone, respectively. Statistical significance was determined by a Mann–Whitney test. *P* values are indicated. (*D*) Complementation of A549 TFRC KO with lentiviral expression of TfR1 increases early IAV replication. Western Blot of total cell lysates from A549 TFRC KO transduced with empty pLVX IRES puro or pLVX TFRC IRES puro. Cells were selected with puromycin and infected with the indicated MOI of PR8 for 3 h. Total cell lysates were separated by SDS-PAGE and analyzed by western blot for NP and TfR1 levels. Equal loading was confirmed by probing for beta actin. A representative blot of four independent experiments is shown. (*E*) A549 CTRL and TFRC KO were infected with 5 MOI of VSV-GFP. Total cell lysates (8 hpi) were separated by SDS-PAGE and analyzed by western blot for VSV-G and TfR1 levels. Equal loading was confirmed by probing for beta actin. A representative blot of two independent experiments is shown. (*F*) Quantification of the VSV-.G signal displayed in panel *G*. Statistical significance was determined by a paired *t* test. *P* values are indicated. (*G*) A549 CTRL and TFRC KO were infected with 0.01 MOI of VSV-GFP. Supernatant were collected after the indicated time points and analyzed by standard plaque assay on MDCK cells. Median titers in pfu/mL are indicated from four independent biological samples in two independent experiments. Statistical significance was determined with multiple *t* tests. *P* values are indicated. (*H*) 293T CTRL (black symbols) and 293T TFRC KO (gray symbols) were infected with Bla-M1 VLPs displaying the HA and NA of WSN or no envelope. Ammonium chloride was used to inhibit endosomal entry. Only cells with substrate conversion (BlaM1 positive) are depicted. Each symbol refers to one independent experiment (n = 3). Statistical significance was determined by a two way ANOVA. Adjusted *P* values are indicated.

TfR1 is a type II transmembrane protein encoded by the transferrin receptor (*TFRC)* gene and was described to serve as an entry receptor for a number of relevant human pathogens (30), e.g., new world arenaviruses (31) or *Plasmodium vivax* (32). It is expressed as a functional homodimer on basically all nucleated cell types, matching the broad tropism of IAV in vitro. In respiratory tissue, TfR1 is expressed predominantly on macrophages but also on the apical side of type I and II pneumocytes (http://www.proteinatlas.org) (33, 34). TfR1 continuously recycles between surface and endosome, where its holo-transferrin cargo releases bound iron in a pH-dependent fashion and converts to apo-transferrin, which then returns complexed with TfR1 to the cell surface. Endosomal uptake of TfR1 occurs, as for IAV particles, through clathrin-mediated endocytosis (reviewed in ref. 35). It is frequently overexpressed in cancer cells (36), which might compromise the relevance of our A549 adenoma cell culture model. To address this, we experimentally compared TfR1 levels in A549 cells and primary human lung epithelial cells from a healthy donor pool, cultured in an air–liquid interface. Primary human lung epithelial cells displayed higher TfR1 protein levels than A549 cells (Fig. 1*A*).

The proximity of the proteins identified by CSPL to attached HA does not necessarily implicate a functional role in viral uptake. In order to assess the impact of TfR1 on IAV replication, we generated four A549 TFRC KO clones by transfection of three independent guide RNAs and four control clones exposed only to Cas9 expression (A549 CTRL). These clonal cell lines were infected with a standard H1N1 laboratory IAV strain [A/Puerto Rico/8/1934 (PR/8)]. Western blots for viral antigen at 4 h post-infection revealed approximately 50 to 60% lower levels of nucleoprotein (NP) in A549 TFRC KO cells compared to A549 CTRL (Fig. 1*B*), consistent with an involvement of TfR1 in IAV entry or early replication steps. Using a luciferase-based minigenome assay, we assessed the impact of the absence of TfR1 on viral genome replication. Using the polymerase complexes of the H1N1 and a H5N1 isolates, we observed no negative effect of TfR1 deficiency on viral polymerase function (*SI Appendix*, Fig. S5 *A* and *B*). This suggested that the TFRC-KO affected an upstream step in the viral replication cycle. Complementation of A549 TFRC KO cells with a lentiviral transduced human TfR1 cDNA restored early replication, confirming the specificity of the KO approach (Fig. 1*D*). Using a pool of our four A549 TFRC knockout vs. four control clones, we demonstrated that the early replication of Sendai virus (SeV) was not affected by the TfR1 depletion (*SI Appendix*, Fig. S6 *A* and *B*). In a multicycle growth curve, SeV-GFP replicated comparably in A549 CTRL or TFRC KO cells (*SI Appendix*, Fig. S6*C*) This virus enters in an endocytosis-independent fashion, by fusing at the plasma membrane (reviewed in ref. 37). To further demonstrate the specificity of the TfR1-dependent support for early IAV replication, we repeated the infection experiments using vesicular stomatitis virus (VSV), an unrelated virus that also enters cells by endocytosis. TFRC-KO and control cells did not differ either in viral protein production (Fig. 1 *E* and *F*) or in the production of infectious virus particles in a multicycle growth curve (Fig. 1*G*).

In order to specifically address the entry phase of the IAV life cycle, we measured the fusion of beta-lactamase-IAV-M1 (Bla-M1) based virus-like particles (VLP) with endosomal membranes (38). Upon fusion, these VLP expose beta-lactamase to the host cell cytoplasm, where it can process a membrane-permeable fluorescent substrate, which in turn shifts fluorescence from the green to blue wavelengths. Since A549 cells are not compatible with this assay (for unknown reasons), we generated 293T TFRC-KO cells. Knockout of TFRC resulted in a 30% reduced median Bla-M1 cleavage of the cytosolic substrate, pointing to a limitation at an early step in the viral replication cycle (Fig. 1*H*), although this was not statistically significant in the experiment we performed (*P* value of 0.076).

### Complementation of Poorly IAV Susceptible Cells with Human TfR1 Increases Viral Entry.

**Fig. 2.**
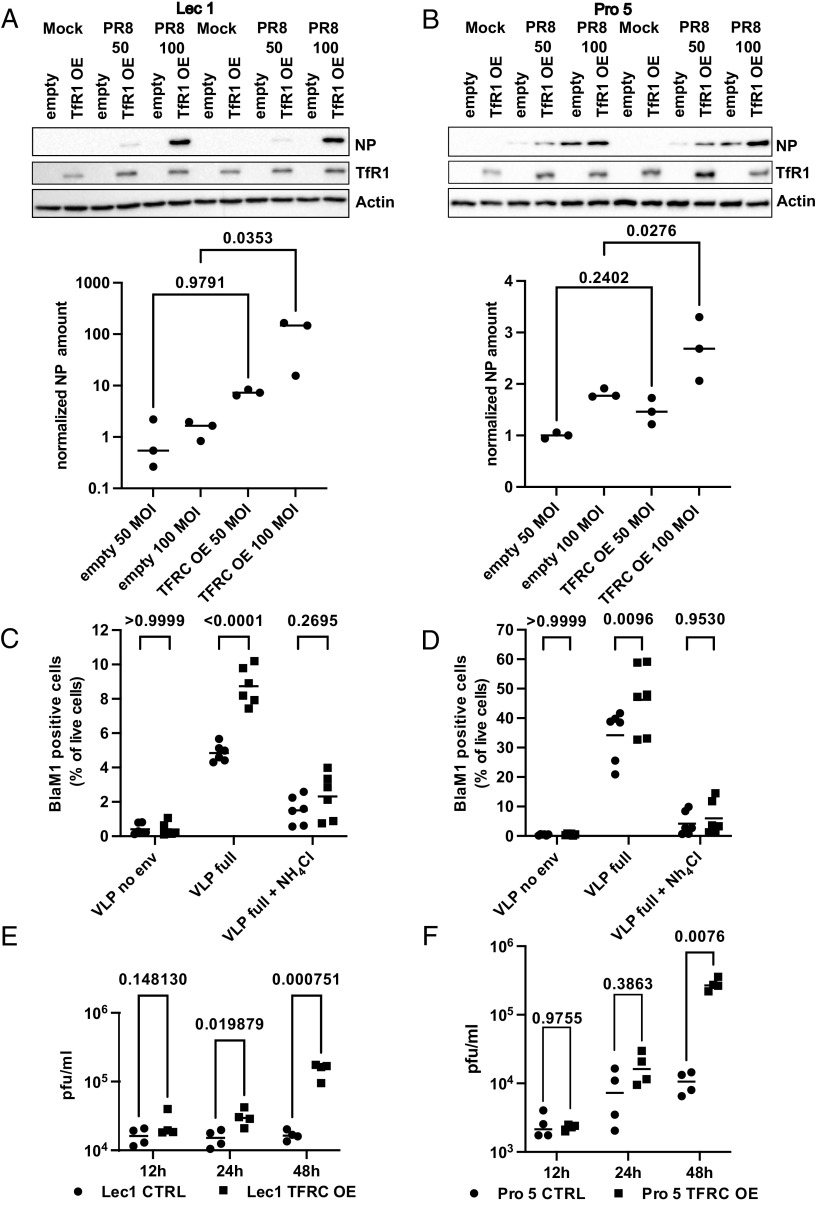
TfR1 expression increases susceptibility of CHO cell clones for IAV entry. CHO Lec1 and CHO Pro5 were transduced with empty pLVX IRES puro (empty) or pLVX TFRC IRES puro (TFRC). CHO Lec1 (*A*) and CHO Pro5 (*B*) infected with indicated MOI of PR8 for 5 h. Total cell lysates were separated by SDS-PAGE and analyzed by western blot for NP and TfR1 levels. Equal loading was confirmed by probing for beta actin. A representative blot of three independent experiments is shown. Quantifications of the NP signal are depicted below. Statistical significance was determined by one-way ANOVA. *P* values are indicated. CHO Lec1 (*C*) and CHO Pro5 (*D*) were infected with BlaM1-VLPs displaying the HA and NA of WSN or no envelope. Ammonium chloride was used as a blocker of endosomal entry. Only cells with substrate conversion (BlaM1 positive) are depicted. (n = 3). Statistical significance was determined by two way ANOVA. Adjusted *P* values are indicated. CHO Lec1 (*E*) and CHO Pro5 (*F*) were infected with PR8 and with MOI of 1 and 0.1 respectively for the indicated time points. Infectious viral particles were quantified by standard plaque assay on MDCK cells. Two independent experiments with biological duplicates were analyzed. Statistical significance was determined by multiple unpaired *t* test. *P* values are indicated.

In a laboratory setting, IAV enters into a large spectrum of cell types. In many of these, its efficient replication generally hampers gain-of-function approaches to identify proviral host dependency factors. Recently, two Chinese hamster ovary (CHO) cell clones (Lec1 and Pro5) were characterized with a very limited capacity for IAV attachment and entry (10). Reasoning that these CHO cell clones could provide a useful experimental model for gain-of-function entry assays. CHO Lec1 and Pro5 were transduced with a lentiviral vector expressing the hTFRC cDNA. In both cell backgrounds, hTFRC expression substantially improved the early infection of IAV as evidenced by NP expression after 5 h (Fig. 2 *A* and *B*) and VLP-Bla-M1 entry (Fig. 2 *C* and *D*). This positive effect of TfR1 expression on IAV infection was further confirmed in multicycle growth curves. In both, PR/8 grew more than tenfold higher at a 48 h post-infection in cells overexpressing TfR1 as compared to control cells (Fig. 2 *E* and *F*). Indeed, increased titers were already visible at 24 h post-infection.

### TfR1 Recycling Is Required for IAV Entry.

To distinguish whether TfR1 is required for virion attachment or entry, we quantified surface-bound virions on A549 TFRC-KO cells and on CHO-Lec1 cells overexpressing human TfR1 using a specific qRT-PCR for vRNA normalized to a standard M1 expressing plasmid (*SI Appendix*, Fig. S7*A*). In comparison with matched control cells, we did not find differences in the number of attached virions (*SI Appendix*, Fig. S7 *B* and *C*).

Under physiological conditions, TfR1 continuously recycles between surface and endosome (39). One could imagine that viruses take advantage of this “revolving door” to enter their host cells via co-endocytosis. We first ruled out that TfR1 deficiency in A549 or overexpression of TfR1 in CHO cells alters the overall endocytic capacity by visualizing the uptake of a TfR1 independent ligand, low densitiy lipoprotein (LDL). No obvious differences in LDL uptake were detected (*SI Appendix*, Fig. S8 *A* and *B*). Along the same line, nonspecific uptake of fluoresecent nanobeads was neither significantly diminshed in A549 TFRC KO cells nor significantly increased in CHO PRO5 TFRC OE cells (*SI Appendix*, Figs. S9 and S10). Together, these data make it improbable that TfR1 nonspecifically increases the endocytotic capacity of cells for random cargo.

**Fig. 3.**
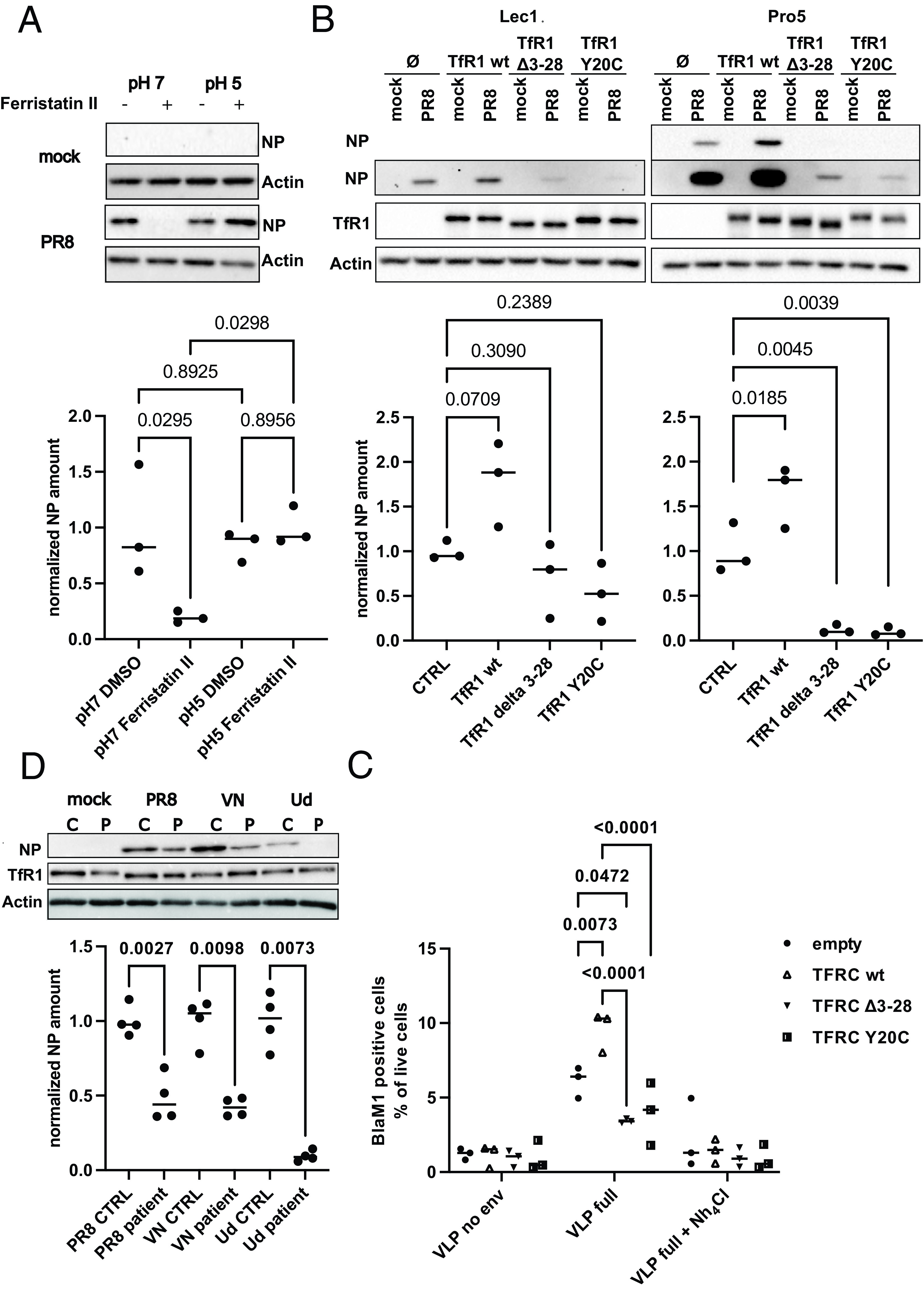
TfR1 recycling is required for IAV entry. (*A*) A549 cells were treated for 4 h with 50 µM of ferristatin II or an equivalent volume of DMSO. Cells were infected with 10 MOI or 20 MOI of PR8 on ice, and were pulsed with pH7 or pH5 medium, respectively. Total cell lysates were separated by SDS-PAGE and analyzed by western blot for NP levels. Equal loading was confirmed by probing for beta actin. A representative blot of three independent experiments is shown. Quantifications of the NP signal are depicted below. Statistical significance was determined by one-way ANOVA. *P* values are indicated. (*B*) CHO Lec1 (*Upper*) or CHO Pro5 (*Lower*) expressing TfR1 wt, TfR1Δ3-28, or TfR1 Y20C. Cells were infected with 5 MOI of PR8 for 4 h. Total cell lysates were separated by SDS-PAGE and analyzed by western blot for NP and TfR1 levels. Equal loading was confirmed by probing for beta actin. A representative blot of three independent experiments is shown. Quantifications of the NP signal are depicted below. Statistical significance was determined by one way ANOVA. *P* values are indicated. (*C*) CHO Pro5 expressing TfR1 wt, TfR1Δ3-28 or TfR1 Y20C were infected with BlaM1-VLPs displaying the HA and NA of WSN or no envelope. Ammonium chloride was used to inhibit endosomal entry. Only cells with substrate conversion (BlaM1 positive) are depicted (n = 3). Statistical significance was determined by two-way ANOVA. Adjusted *P* values are indicated. (*D*) Control and patient skin fibroblasts (homozygous for TFRC Y20H substitution) were infected with 5 MOI of the indicated viruses for 4 h. Total cell lysates were separated by SDS-PAGE and analyzed by western blot for NP and TfR1 levels. Equal loading was confirmed by probing for beta actin. A representative blot of two independent experiments is shown. Quantification of the NP signal is depicted on the right hand side. Statistical significance was determined by one way ANOVA. *P* values are indicated.

IAV virions enter mostly through clathrin-mediated endocytosis, fusing at approximately pH 5.0 to 5.5 in the late endosome (reviewed in ref. 15). Using an endosomal acid-bypass approach (40), we investigated whether inhibition of TfR1 affects viral entry efficacy at the plasma membrane or in the endosome. We applied nontoxic concentrations of ferristatin II, a recently described chemical inhibitor of TfR1 (41) (*SI Appendix*, Fig. S11 *A* and *B*), to interfere with TfR1 function during viral entry at different pH conditions. At neutral pH, when virions enter through endocytosis, ferristatin II eliminated early IAV replication (Fig. 3*A*). Strikingly, virions pulsed at pH 5, and thus fusing at the plasma membrane, were not affected by ferristatin II, implying that the TfR1 function during IAV entry is not required when virions directly fuse at the cell surface.

Clathrin-mediated endocytosis of TfR1 depends on the N-terminal YXXΦ-motif (aa20-23) (42, 43). Substitution of Y20 with nonaromatic residues or depletion of the whole motif results in dramatically slower TfR1 recycling (44). When we complemented low-susceptible CHO Lec1 or CHO Pro5 cells with two loss-of-function mutants of TfR1 [Y20C substitution mutant and a Δ3-28 deletion mutant (42, 43)], early IAV replication levels fell below the level observed in the parental CHO cells (Fig. 3*B*). Further support for the requirement for TfR1 recycling for virion entry was obtained in a BlaM1 uncoating assay (Fig. 3*C*).

Consistent with this, patient derived fibroblasts with a homozygous mutation in TfR1 (Y20H) (45) were less permissive to IAV replication as compared to control fibroblasts (Fig. 3*D*), indicating the importance of TfR1 recycling for IAV infection of primary human cells. Importantly, the very same patient-derived cells showed no early replication defect for VSV entry (*SI Appendix*, Fig. S12*A*) and even higher replication levels for SeV than control fibroblasts (*SI Appendix*, Fig. S12*B*). The molecular basis for this enhancement of SeV replication remains elusive. Regardless, these findings support a specific proviral function of TfR1-mediated endocytosis for IAV entry.

### TfR1 Promotes Virion Uptake in Cis and Trans.

**Fig. 4.**
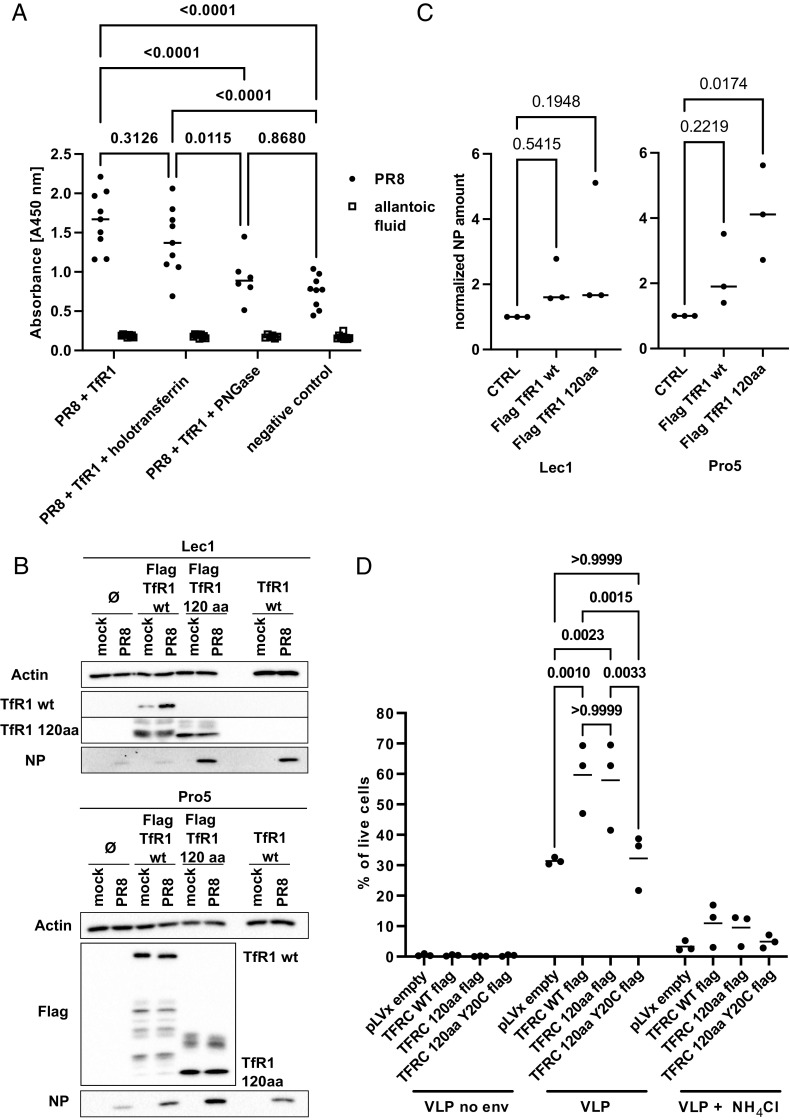
TfR1 supports IAV uptake in cis and trans. (*A*) Two-step ELLA assay. TfR1 coated wells and control wells were incubated with 5 × 10^8^ pfu of PR8. PNGase pretreated TfR1 was used to evaluate the involvement of sialic acids in IAV TfR1 interaction. Attached viruses were incubated with fetuin. Cleaved fetuin was transferred to a new well, incubated over night and probed with peroxidase conjugated lectin from *Arachis hypogaea*. Absorbance at 450 nm for three independent experiments with technical triplicates is shown. Statistical significance was determined by two-way ANOVA. Adjusted *P* values are indicated. (*B*) CHO Lec1 or (*C*) CHO Pro5 expressing Flag-TfR1wt or headless Flag-TfR1 120aa. Cells were infected with five MOI of PR8 for 4 h (n = 3). Total cell lysates were separated by SDS-PAGE and analyzed by western blot for NP and TfR1 levels. Equal loading was confirmed by probing for beta actin. Quantification of the NP signal for panel *B* and *C* is depicted below. Statistical significance was determined by one way ANOVA. *P* values are indicated. (*D*) CHO Pro5 expressing Flag-TfR1wt or headless Flag-TfR1 120aa or headless Flag-TfR1 120aa with a Y20C substitution. Cells were infected with BlaM1-VLPs displaying the HA and NA of WSN or no envelope. Ammonium chloride was used to inhibit endosomal entry. Only cells with substrate conversion (BlaM1 positive) are depicted (n = 3). Statistical significance was determined by multiple *t* test. *P* values are indicated.

Next, we addressed if IAV HA directly binds to TfR1 to enter host cells. TfR1 purified from 293T did not co-precipitate with trimeric HA (*SI Appendix*, Fig. S13). We further investigated if holotransferrin might bridge HA and TfR1, but pulldown experiments gave no indication for such a hetero-trimeric complex (*SI Appendix*, Fig. S13). Since viral particles bind to their host cell surface in a multivalent fashion due to the low affinity of HA to sialic acid (46), we applied a two-step enzyme-linked lectin assay (2-step ELLA) to measure IAV particle binding to a TfR1-coated plate (Fig. 4*A*). Desialylation of fetuin by attached IAV particles was monitored as a readout and increased twofold over background when TfR1 was used as a substrate to bind particles. The addition of holotransferrin neither increased nor decreased IAV particle attachment, implicating a direct and probable multivalent interaction between IAV particles and TfR1 (Fig. 4*A*). Desialylated TfR1 was not bound by virus particles, suggesting that contact involves post-translational modifications (Fig. 4*A*).

Endosomes transport a very diverse set of host proteins (47) and this could be exploited by IAV during the attachment step. In this scenario, TfR1 could support IAV particle uptake in *trans*, e.g., when virions are bound to a different cell surface protein in the proximity of TfR1. To challenge this hypothesis, we tested if a “headless” TfR1 truncation mutant (aa1-120), would support IAV entry. This headless version of TfR1 internalizes at rates comparable to full-length TfR1 (48). Importantly, expression of TfR1-120 supported IAV uptake events at similar or even higher levels than TfR1 wt (Fig. 4 *B* and *C*). However, it should be noted that the statistical analysis of signal intensity did not reach significance due to a substantial positive deviation in the TfR1 expressing cells. Importantly, in each case, we observed a qualitatively positive effect on IAV replication when either TfR1 or the 1-120aa mutant were expressed. This supports the hypothesis that direct interaction with HA is not required for TfR1-promoted virion entry. As with full-length TfR1, endocytosis was essential for the enhanced IAV uptake by TfR1 1-120 as the TfR1 1-120 Y20C mutant cell line behaved like parental CHO Pro 5 cells. (Fig. 4*D*).

### Colocalization of HA and TfR1 during VLP Entry.

**Fig. 5.**
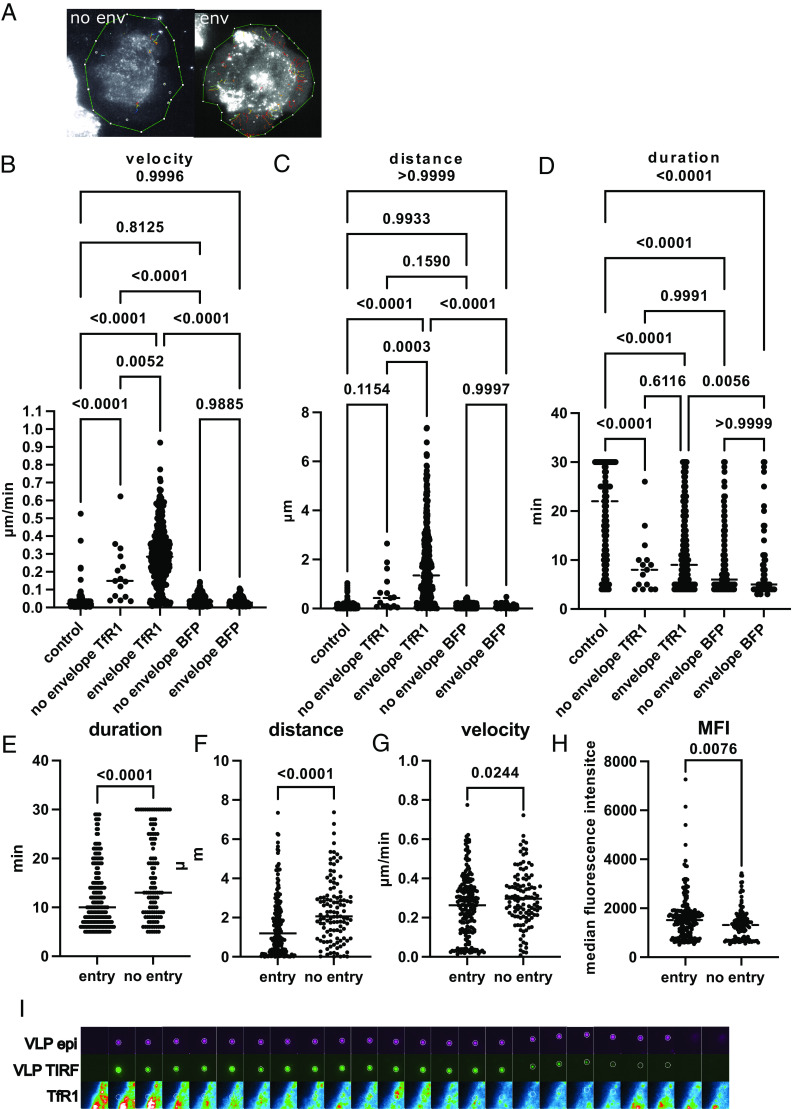
TfR1 colocalizes with entering IAV. CHO Pro5 TfR1 BFP or CHO Pro5 BFP were incubated with mNeonGreenM1 VLP and monitored for 30 min with a TIRF microscope. (*A*) Visualization of tracks recorded with HA/NA enveloped VLP or non enveloped VLP. Representative cells are shown. Detected VLP is indicated with a white circle. Tracks are color-coded for the starting time point (blue: beginning of Movie S1, red: end of it). (*B*–*D*) Comparison of track duration in min (*B*), track distance in µm (*C*), and mean VLP velocity in µm/min (*D*) for control tracks (HA/NA enveloped VLPs outside of the cell surface area, n = 269), tracks of non enveloped VLP (lacking HA/NA, n = 15) on the cell surface of TfR1 expressing cells, tracks of HA/NA enveloped VLP on the cell surface of TfR1 expressing cells (n = 371), tracks of non enveloped VLP (lacking HA/NA, n = 254) on the cell surface of BFP expressing cells and tracks of HA/NA enveloped VLP on the cell surface of BFP expressing cells (n = 66). Each dot represents a single track. Control tracks and enveloped VLP tracks on cell surfaces were recorded from three cells in three independent experiments, tracks from nonenveloped viruses were recorded from three cells in one experiment. Median values are indicated by a line, *P* values were calculated with one way ANOVA. (*E*–*G*) Tracks from enveloped VLPs on cells organized into entering VLPs (TIRF signal lost before frame 30, n = 199) and non entering VLPs (TIRF signal remains until frame 30, n = 113). Track duration (*E*), track distance (*F*) and VLP velocity (*G*) are indicated as for *B*–*D*, with the respective median indicated by a line. *P* values were calculated by *t* test. (*H*) Median fluorescence intensity of TfR1-BFP along the tracks of VLPs that enter or do not enter until frame 30. Each dot represents a single data point of individual tracks. Only tracks of six or more frames were considered. (*I*) Example of an enveloped VLP entering the host cell. The VLP is tracked in TIRF and epifluorescence mode (*Upper* rows). TfR1-BFP was solely monitored in TIRF mode.

IAV particles move laterally along the host cell surface after attachment (17). Using total internal reflection (TIRF) microscopy we assessed, if IAV VLP would co-migrate with TfR1 during this lateral movement process and if this correlated with successful entry. In order to reduce background entry through alternative surface proteins we used low-susceptible CHO-Pro5 cells expressing a BFP-TFR1 fusion protein or a BFP anchored via a PDGFR transmembrane protein at the cell surface. Both cells were infected with fluorescent IAV-VLP containing mNeonGreen-M1, designed based on Bla-M1 (38). We tracked VLP expressing HA and NA (enveloped) or VLP without surface glycoproteins (nonenveloped) on individual cells and determined colocalization with TfR1 during a 30-min time window. HA/NA decorated VLP traveled on average ~2 µm at a speed of ~0.3 µm/min. Generally, VLP without viral surface proteins were rather immobile compared to HA/NA decorated ones (Fig. 5*A*), confirming the essential interplay of attachment and detachment during this process (18).

For HA/NA decorated VLP, we found a clear increase of track duration, track distance, and particle velocity as compared to the naked VLP (Fig. 5 *B*–*D*). The same VLP remained immobile when recorded outside of the cell surface (control in Fig. 5 *B*–*D*). Cells overexpressing only a BFP with a transmembrane domain did not support VLP attachment or movement above background levels. Overall, only the combination of HA/NA decorated VLP with cells expressing TfR1 resulted in rapid and enduring VLP movement.

When comparing HA/NA enveloped VLPs which entered the cells during the 30-min observation period with those that did not (present in the TIRF plane until frame 30), we found shorter track duration and distance and a lower particle velocity. Notably, entering particles were colocalizing more with TfR1-BFP (Fig. 5 *E*–*G*). This colocalization did not occur predominantly before entering but rather in a dynamic fashion throughout the full length of the track (Fig. 5*I* and Movie S1). Accordingly, we did not detect an increase in VLP-TfR1 colocalization from the beginning to the end of the recorded tracks (*SI Appendix*, Fig. S14).

### Pharmacological Inhibition of TfR1 Reduces Viral Entry In Vitro and Limits Viral Replication In Vivo.

Finally, we asked if TfR1 could be a pharmacological target for IAV infection. We first demonstrated that nontoxic concentrations of ferristatin II (*SI Appendix*, Fig. S11 *A* and *B*) have indeed a broad inhibitory capacity for different IAV strains, including a recent H3N2 clinical isolate (*SI Appendix*, Fig. S15 *A*, *Right*). Multicycle growth curves in the presence of ferristatin II, revealed lower viral titers at 24 h post-infection for three different subtypes. The isolates Neth602 (H1N1) and Wy03 (H3N2) were replicating less even at 48 h post-infection (*SI Appendix*, Fig. S15*C*). Curiously, we did not observe a degradation of TfR1 in ferristatin II-treated cells, as reported earlier (*SI Appendix*, Fig. S16 *A* and *B*). Ferristatin II specifically inhibited virion entry, as shown by the BlaM1 assay (*SI Appendix*, Fig. S16*C*). We further demonstrated that ferristatin II-dependent inhibition of early IAV replication is abolished in TFRC-KO cells (*SI Appendix*, Fig. S16*D*), suggesting that ferristatin II-dependent inhibition of IAV entry fully relies on TfR1 antagonims. In contrast, SeV and VSV protein levels in infected cells were unaffected by ferristatin II. (*SI Appendix*, Fig. S16 *E*–*G*). Accordingly, VSV-G pseudotyped HIV VLP encoding for a Gaussia luciferase reporter generated a comparable signal in DMSO and ferristatin II-treated cells (*SI Appendix*, Fig. S16*H*). However, a multicycle growth curve of VSV in presence of ferristatin II resulted in significantly lower titers at 8 h and significantly higher titers at 24 h post-infection (*SI Appendix*, Fig. S16*I*). This is inconsistent with the unaffected replication of VSV in TFRC-KO cells (Fig. 1*G*) and the VLP entry assay (*SI Appendix*, Fig. S16*H*) and might point to a VSV-specific effect of ferristatin II at later stages of VSV replication. Similarly, ferristatin II did not diminish the number of GFP-positive A549 cells after 24 h and 48 h when using a low MOI of 0.5 (*SI Appendix*, Fig. S16*J*).

Additionally, we tested if inhibition of TfR1 in vivo could reduce viral replication in the lung in a small animal model. Adult C57BL/6J mice underwent a pre-administration schedule of i.p. injections at nontoxic concentrations of ferristatin II before intranasal infection with IAV (*SI Appendix*, Fig. S17*A*). Treatment with ferristatin II was discontinued after the infection. Four days post-infection, we measured a 10-fold reduced viral lung titer in total lung homogenates using a standard plaque assay (*SI Appendix*, Fig. S17*B*). The same trend was still detectable at D6 post-infection but did not reach statistical significance. In an attempt to increase the relevance of this finding for human patients, we treated precision-cut lung slices (PCLS) of healthy human lung tumor bystander tissue from two donors (*SI Appendix*, Table S1) with ferristatin II or DMSO. When infected with Wy03, these PCLS displayed substantially reduced replication kinetics in the presence of ferristatin II (*SI Appendix*, Fig. S15*D*).

These data show that short-term interference with the TfR1 function could be a strategic target for future antiviral drug development.

## Discussion

In conjunction with previous data, we extend the multistep model of IAV host cell entry, in which virions first attach via HA to sialylated host surface proteins. The subsequent lateral rolling allows the identification of an appropriate entry point. In our view, TfR1, and possibly other recycling surface proteins with a reasonable turnover time and cell surface density, could mark such an entry point. Clearly, the residual entry into TFRC-KO cells strongly suggests a role for alternative entry factors. Of the already described entry factors (EGFR, CaV1.2, nucleolin, FFAR2), none were enriched in our CSPL assay (EGFR was found solely in one of the three experiments). This might arise due to the lack of multivalent binding, as observed with assays using full virus. A lower surface density of these factors in our cells as compared to cells used in other labs, would also reduce the sensitivity of the assay. A recent study also employed chemical crosslinking to identify host surface proteins in the vicinity of attached IAV particles (49). Entry enhancing, blocking, and neutral proteins were identified and confirmed using siRNA-mediated knockdown. Curiously, TfR1 was not among the identified hits. The trivalent binding of our probe, *vs.* the multivalent binding of full virions, or differences in the proximity ligation approaches, could explain these differences. It remains unclear if TfR1 acts downstream or in parallel with factors such as EGFR or CaV1.2, during virion uptake. For other viruses (HIV, HCV), a stepwise use of multiple receptors has been proposed (50, 51).

With TfR1, we observed an improved attachment of virions to artificial surfaces coated with the recombinant TfR1 ectodomain. This binding depended on sialidation of TfR1. Functionally, overexpression of human TfR1 improved the susceptibility of CHO cells to virus infection. Binding to an entry factor through sialic acid modifications was proposed for EGFR (21) and Ca_v_1.2 (24). However, the gain-of-function experiments in Lec-1 CHO cells (deficient in N-glycosylation) suggest that this is not necessarily required when using a high virus inoculum.

Intriguingly, we found that TfR1 binding of virions is detectable in vitro but is not required for TfR1-dependent uptake into low-susceptible cells, suggesting that TfR1 recycling could act positively on IAV entry in *cis* and in *trans*. This opens the possibility that attachment and entry could actually be promoted by independent host surface proteins as described for other viruses. Notably, TfR1 is enriched in endosomes importing EGFR (47), a second entry factor described for IAV (21). For the entry of IAV, *trans* acting entry factors would require less adaptation of viruses to the host cell and even the host species, a strategic advantage for zoonotic viruses such as IAV. It also implies that the role of TfR1 in IAV entry is distinct from that on New World arenavirus entry (31) or Plasmodium vivax entry (32), where defined protein-protein interfaces between pathogen surface proteins and TfR1 were identified by structural approaches. While TfR1 was implicated in the entry process of several intracellular pathogens, it appears not to be a generic uptake factor for virions, since VSV does not display TfR1 dependence, or for general endocytosis, e.g., of inert beads.

Our in vitro and in vivo data using ferristatin II suggest that chemical targeting of TfR1 might be a therapeutic approach to limit IAV entry. While we observed different sensitivites among IAV strains to ferristatin-dependent inhibition, further work is required to identify which viral properties (particle shape, fusion pH etc.) define this drug sensitivity. Notably, ferristatin II was shown to inhibit SARS-CoV-2 replication in vitro (52). Both fusion at the plasma membrane and endosomal uptake are possible entry routes for SARS-CoV2 (53). TfR1 dependence might be a consequence of the latter in cell culture. The potency of TfR1 inhibition on SARS-CoV-2 entry in vivo has currently not been addressed. At this stage, we cannot rule out that TfR1 might be a broadly active entry factor for viruses using an endosomal fusion strategy, despite the fact that VSV entry was, in our hands, only mildly affected by ferristatin II.

In summary: We are proposing a “revolving door” model (*SI Appendix*, Fig. S18), in which IAV takes advantage of the continuous recycling of TfR1 between surface and endosome to enter the host cell.

### Limitations.

While we provide quantitative assays (Bla-M1 VLP entry assay, viral growth curves), the western blot analysis for early viral protein production showed in some instances experimental variation, leading nonsignificant test results. We would like to stress that the trend in each western blot experiment is, however, supported by our results. Additionally, these qualitative data are supported by the aforementioned quantitative assays, and hence we believe the overall notion that TfR1 is important for entry of IAV, is justified.

As mentioned above, TfR1 is involved in uptake of other viral pathogens. Here we solely used VSV and SeV as negative controls for the specificity of TfR1 for IAV entry. A broader panel of viruses might have been useful to determine the general importance of this surface protein for the entry of other virus families. Clearly, TfR1 acts as entry factor beyond IAV biology.

A control gRNA was not used to generate the A549 and 293T CTRL cells (these express only the empty Cas9 construct). While we used three different gRNAs we cannot entirely rule out the possibility that there expression could contribute to the reduction in entry.

Our data rely in large part on transformed cell lines, since they are easier to manipulate. In contrast, genetic manipulation of primary human cells is still challenging, specifically when targeting essential genes such as TfR1. Consequently, we relied on the use of a chemical inhibitor for the tests in PCLS to support our conclusions from immortalized cell line models.

While this is important for the relevance as an antiviral drug pathway, chemical inhibition might have some off-target effects. It is worth mentioning that the inhibitory effect of ferristatin II on IAV replication was abolished in A549 TFRC KO cells. For VSV virions in cellular supernatants, we found at 8 h inhibition with ferristatin II (but increased titers at 24 h post-infection). A VLP entry assay with VSV-G pseudotyped HIV VLPs did not show differences in reporter expression between DMSO and ferristatin II-treated cells. In contrast, VSV replication was unaffected in TFRC KO cells and early protein production was not altered, neither by ferristatin II treatment nor by TFRC KO. We currently do not have a good explanation for this contradiction, other than ferristatin II could affect later steps in VSV replication.

While our TIRF data suggest a functional association between virion uptake (as shown by fluorescent VLP) and transient colocalization with TfR1, we could not provide high-resolution microscopy data supporting the colocalization under static conditions.

## Materials and Methods

### Materials Availability.

All unique/stable reagents generated in this study are available from the lead contact with a completed materials transfer agreement.

### Ethical Approval.

Work with human patient material was approved by the internal review board under ref ID 2022-01942. All human donors of lung tissue for the generation of PCLS provided informed written consent. Patient samples were deidentified. Animal experiments were approved by institutional and cantonal authorities under the reference GE-7-20.

### Cell Surface Proximity Ligation Assay.

A549 cells were grown in six-well format and incubated with 50 µg of recombinant HA-HRP or HRP alone for 60 min. For the pre-run experiment we used the same protein to cell ration in a 24-well format (10 µg of protein/well). Biotin phenol and H_2_O_2_ were added for 10 min to allow proximity ligation of biotin. Cells were quenched and lysed with lysis buffer (0.4% SDS, 500 mM NaCl, 5 mM EDTA, 50 mM Tris-HCl pH 7.5, 1% Triton-X100, 1 mM DTT, protease inhibitor). Biotinylated proteins were precipitated with streptavidin-agarose beads and prepared for mass spectrometry by on-bead trypsin digest.

### Viral Infection.

Different cell lines were seeded in 12-well plates at subconfluent density (approximately 80%) and infected with each virus at indicated MOI. Three to five hours post-infection, cells were washed 1× with PBS and lysed using protein lysis buffer. Samples were sonicated, heating at 95 °C for 5 min, and then centrifuged at 10,000 rcf for 5 min. Samples were then used in a western blot and blotted for actin, TFR1, influenza NP, VSV-G or SeV N. Viral protein bands were quantified using Biorad Imaging software and normalized to the actin band of the same sample.

### Viral Growth Kinetics.

Cells were seeded in 12-well plates at subconfluent density (80%) and infected with each virus at a MOI of 0.01 to 0.05 in triplicate in the presence of l-1-tosylamide-2-phenylethyl chloromethyl ketone-treated trypsin (Sigma; T1426). Supernatants were collected at the desired time post-infection. Virus titers were determined by plaque assay in MDCK cells.

### Statistics.

Statistical analysis was performed using GraphPad Prism 9. Statistical tests applied are indicated in each respective figure legend.

## Supplementary Material

Appendix 01 (PDF)

Dataset S01 (XLSX)

Dataset S02 (XLSX)

Dataset S03 (XLSX)

Dataset S04 (XLSX)

Dataset S05 (XLSX)

Dataset S06 (XLSX)

Dataset S07 (XLSX)

Movie S1. Video corresponding to **Fig. 5I**

## Data Availability

Code data have been deposited in Github (https://github.com/Mitchzw/viral-tracking) (54). All study data are included in the article and/or *SI Appendix*.
