# Supplementary material for: Influenza A virus exploits transferrin receptor recycling to enter host cells
Source: Proc Natl Acad Sci U S A. 2023 May 16;120(21):e2214936120. doi: 10.1073/pnas.2214936120 (PMC10214170; doi:10.1073/pnas.2214936120)
Supplement: Supplementary file 1 — Appendix 01 (PDF) [file pnas.2214936120.sapp.pdf]

Supplementary Material and Methods

Cell lines and primary cells

A549 (adenocarcinomic human alveolar basal epithelial cells, ATCC), A549-derived cell lines, and HeLa cells (cervical adenocarcinoma epithelial cells, ATCC) were grown in DMEM/F12+ GlutaMAX (Dulbecco's Modified Eagle Medium/Nutrient Mixture F-12, Gibco 10565018). HEK 293T (human embryonic kidney, ATCC), MDCK (Madin-Darby canine kidney, ATCC) and HepG2 (hepatocellular carcinoma cells, ATCC) were grown in DMEM (Dulbecco's Modified Eagle Medium, Gibco 10566016). CHO (Chinese Hamster Ovary) clone Pro5 and clone Lec1 were grown in MEMα with Ribonucleosides, Deoxyribonucleosides and GlutaMAX (Minimum Essential Medium alpha, Gibco 32571028). Human fibroblast with homozygous Y20H substitutions in TfR1 were kindly provided by Dr. Raif Geha, Boston Children's Hospital and cultured in DMEM+GlutaMax (Dulbecco's Modified Eagle Medium, Gibco 10566016). Control skin fibroblasts (HFF, ATCC-CRL-2429) were kindly provided by the Dr. Dominique Soldati-Favre, University of Geneva and cultured under the same conditions. Calu3 (lung epithelial adenocarcinoma cells) were grown in MEM+GlutaMAX (Gibco 41090-28) + 1x MEM non-essential amino acids 100X (Gibco 11140-035) + 10mM HEPES (Gibco 15630-056) + 1mM sodium pyruvate (Gibco 11360-039). Cell culture media were supplemented with 10 % (v/v) heat-inactivated foetal bovine serum (Gibco 10270-106. Lot: 2307592) and pen-strep antibiotics (100 U/ml penicillin and 0.1mg/ml streptomycin, Sigma-Aldrich P0781). Fully differentiated human tracheobronchial epithelial cell grown as pseudostratified 3D tissue in air liquid interface (MucilAir™ Pool of Donors, EP02MP) were purchases from Epithelix (Switzerland) and cultured according to the manufacturer's conditions. All cells were

maintained in low passage at 37 °C with 5 % CO2 and 90 % humidity, absence of mycoplasma was routinely confirmed by PCR(1).

Precision cut lung slices (PCLS)

PCLS were generated in line with published protocols [1,2]. Briefly, healthy human lung tissue was obtained from bystander material of tumor resections of two patients. The airways were perfused with 2% low melting agarose and tissues were rested for 30 min at 4°C. 8mm ∅ tissue cores were generated with an electric coring press and transversely cut into 250 µm thick PCLS with a MD6000 Krumdieck tissue slicer in cold PBS (both instruments Alabama Research and Development). PCLS were cultured in 24-well plates with DMEM F-12 supplemented with antibiotics (100 U/ml penicillin, and 100 µg/ml streptomycin, w/o fetal bovine serum) at 37°C, 5 % CO_2 and 95 % humidity for 24 h before the experiment. For PCLS from each patient two independent sets of infections were performed in biological triplicates. Supernatants of PCLS were collected at indicated time points and viral titers were determined by standard plaque assay on MDCK cells.

Plasmids

pDZ plasmids contain a bidirectional expression cassette for a given influenza A virus gene segment and have been described previously(2, 3). pCAGGS-based expression plasmids contain protein-coding sequences under the control of the chicken β-actin promoter(4). pCAGGS.WSN-HA, pCAGGS.WSN-NA, pCAGGS.WSN-M2 and pCAGGS.WSN-BlaM1 were a gift of Adolfo Garcìa Sastre (Icahn School of Medicine at Mount Sinai, New York, NY). pMD2.G and psPAX2 were a gift from Didier Trono (Addgene; plasmid 12259 and 12260) pLVX-IRES-Puro was purchased from Clontech (#632183). pSpCas9(BB)-2A-GFP (PX458) was a gift from Feng

Zhang (Addgene; plasmid 48138)(5). The cDNA endoding for BFP was inserted via BglII and SalI into pDisplay (Invitrogen). The entire surface expression cassette including the BFP ORF were cut from pDisplay via EcoRI and NotI digest and inserted into pLVX-IRES-puro.

Antibodies

Antibodies for immunoblotting include: mouse anti-actin HRP (Abcam #ab49900), mouse anti-Flag horseradish peroxidase (HRP) (Sigma #A8592), rabbit anti-TFR1 (Sigma-Aldrich #HPA028598), mouse anti-CD44 (Cell Signaling #3570), rabbit anti-influenza NP (ThermoFisher #PA5-32242), mouse anti-Sendai N (a gift from Dr D. Garcin, University of Geneva), mouse anti-VSV G (Kerafast #EB0010), streptavidin-HRP (Thermofisher S-911).

Antibodies for IF: rabbit anti-TFR1 (Sigma-Aldrich #HPA028598), mouse anti TfR1 (OKT9, Thermofisher #17-0719-42), rat anti-CD44 Invitrogen 14-0441-82), goat anti-Rat IgG secondary antibody Alexa Fluor™ 555 (Invitrogen #A-21434), Streptavidin-Alexa Fluor™ 488 (Jackson ImmunoResearch # 016-540-084).

Oligonucleotides for qPCR and gRNA cloning

All oligonucleotides were purchased from Microsynth (France). M1_fw: AGATGAGTCTTCTAACCGAGGTCG, M1_rev: TGCAAAAACATCTTCAAGTCTCTG, TFRC_gRNA1_fw: CACCGCTGAACCGGGTATATGACAA, TFRC_gRNA1_rev: AAACTTGTCATATAC CCGGTTCAGC, TFRC_gRNA2_fw: CACCGCTATACGCCACATAACCCCC, TFRC_gRNA2_rev: AAACGGGGGTTATGTGGCGTATAGC, TFRC_gRNA3_fw: CACCGCAATATAAGCGACGTGCTGC, TFRC_gRNA3_rev: AAACGCAGCACGTCGCTTATATTGC. Guide RNAs were designed using ChopChop v3(6) and cloned into pSP.Cas9wt.GFP (see below).

Viruses

The following viruses were generated by reverse genetic: PR8 (A/Puerto Rico/8/1934 (H1N1)), VN1203 (A/Vietnam/1203/2004 (H5N1)), Neth602 (A/Netherlands/602/2009 (H1N1)), WSN (A/WSN/1933 (H1N1)), Udorn (A/Udorn/1972 (H3N2)), Wyo03 (A/Wyoming/03/2003 (H3N2)). The clinical H3N2 (A/Switzerland/9715293/13-like) isolate of IAV was provided by the Infectious diseases division of the Geneva University hospitals (HUG). The pDZ plasmid were provided by Dr Adolfo Garcìa-Sastre and Dr Peter Palese (Icahn School of Medicine at Mount Sinai, New York, NY). Briefly, recombinant viruses were produced using the eight-plasmid rescue system (62). 35 mm dishes of sub-confluent 293T cells were transfected with 0.5 µg of each pDZ plasmid using Lipofectamine 2000 transfection reagent (ThermoFisher). 24 h post-transfection, 200 µL of 293T supernatant was used to infect 10 days old chicken eggs or MDCK cells. Allantoic fluids or supernatant were harvest 44-48 h post-infection. Unique viral clones were isolated after plaque assay on MDCK, amplified in eggs or MDCK cells and their genome fully sequenced. For virus infections, cells were washed once in PBS, viruses were diluted in PBS-0.2 % bovine serum albumin (BSA) and added to cell monolayer. 45 min post infection, cells were washed once with PBS and 1mL of infection media was added (DMEM/F12 with 0.2 % BSA and 1 % Pen/Strep). VSV-GFP and SeV-GFP were kindly provided by Dr Dominique Garcin, University of Geneva(7, 8).

Generation of a KO cell line using CRISPR/Cas9

Subconfluent A549 or 293T cells in 35-mm dishes were transfected with 2 µg of plasmid pSP.Cas9wt.GFP or pSP.Cas9wt.GFP.TFRCsgRNA (3 different ones) using 3 µl of Fugene (Promega)/µg DNA for A549 or 2µl of Trans-IT LT1 (Mirus)/µg of DNA for 293T according to

the manufacturer's protocol. Seventy-two hours post transfection, green fluorescent protein (GFP)-positive cells were sorted individually into 96wells. For A549 we obtained four TFRC KO clones for 293T one TFRC KO clone. The efficiency of knockout was assessed by Western blotting. Cell lines generated using a guide RNA directed against TFRC are named TFRC KO and those transfected with an empty pSP.Cas9wt.GFP plasmid are indicated as CTRL.

Mass spectrometry

Proteins were trypsin-digested on-beads and peptides were analysed by nanoLC-MSMS using an easynLC1000 (Thermo Fisher) coupled with a Qexactive Plus mass spectrometer (Thermo Fisher). Database search was performed with Mascot (Matrix Science) using the Human Reference Proteome database (Uniprot). Proteins were cross referenced with the cell surface protein atlas(9). Biotin-tyramide was added as additional variable modification. Data were analysed and validated with Scaffold (Proteome Software) with 1% of protein FDR and at least 2 unique peptide per protein with a 0.1% of peptide FDR. For identification of host surface proteins specifically enriched in the vicinity of HA-HRP we applied the following cutoffs: 1. Protein must be 1.5fold enriched of HRP control, 2. Protein must be present with five peptides in three independent runs, 3. Protein must be present in less than 100/713 experiments of the CRAPome database for biotinylation experiments using human cells.

Generation of overexpressing cell lines with lentiviral transduction systems

Subconfluent 293T cells were transfected at a ratio of 1:3:4 with the following plasmids: pMD2.G (vesicular stomatitis virus G protein [VSV-G]), psPAX2 (HIV gag-pol), and pLVX.IRESpuro or pLVX.TFRCx.IRESpuro (where x is either: wt, Δ3-28, Y20C, 120aa, wt-flag,

120aa-flag, 120aa Y20C-flag or wt-BFP) using 2 µl/µg of DNA of Trans-IT LT1 (Mirus) according to the manufacturer's protocol. Twenty-four hours post transfection, 293T medium was replaced with target cell medium. Target cells (A549 TFRC KO, CHO Pro5 or Lec1) were seeded in a 6-well plate at subconfluent density (50%). At 48 h post transfection, the 293T cell supernatant containing lentiviruses was harvested with a syringe and pressed slowly through a 0.44-µm sterile filter. The filtered supernatant was complemented with 8 µg/ml of Polybrene. Target cells were washed one time with PBS before addition of 2 ml of the supernatant-Polybrene mixture. Four hours after infection with lentiviruses, the 293T cell supernatant was removed from target cells and replaced with the appropriate medium. Two days post infection, target cells were split and subjected to selection using puromycin at 2 µg/ml for A549, 6 µg/ml for CHO Lec1 or 8 µg/ml for CHO Pro5. The efficiency of overexpression was assessed by WB. Cells generated using a vector expressing TfR1 are named TFRC OE, and those using an empty vector are named CTRL.

Minigenome assay

Minigenome assays were performed as described previously(10). Briefly, cells were transfected with pCAGGS expression plasmids for PB2, PB1, PA and NP and a human Pol1-driven negative sense Luciferase reporter flanked by IAV promoter regions of the NS segment(11). pCAGGS Renilla luciferase was contransfected as a control. Dual luciferase Reporter assays were performed 24h post transfection according to the manufacturers instructions (Promega).

Plaque assay

A confluent monolayer of MDCK cells was infected with 200 µl of serially diluted virus. Viruses were diluted in PBS–0.2 % (wt/vol) bovine serum albumin (BSA) (Millipore; 126579).1h post infection, the inoculum was removed and an agarose overlay (final concentrations: 1X minimal essential medium (MEM), 1 % agarose, 100 mM L-glutamine, 2.5 % sodium bicarbonate, 0.5 M HEPES, 5 mg/ml of Pen-Strep, 0.2 % BSA, and 0.01 % DEAE-dextran) was added. Cells were incubated at 37 °C for 40-48h. Then cells were fixed in 4% formaldehyde, the overlay was removed, and the cell monolayer was stained with a solution of crystal violet.

Virus-Like Particles (VLP) production

To generate WSN BlaM1 VLPs, sub-confluent 35 mm-dish of 293T cells were transfected with 2.5 µg pCAGGS.WSN-BlaM1, 0.5 µg each of pCAGGS.WSN-HA and pCAGGS.WSN-NA and 0.2 µg of pCAGGS.WSN-M2 using TransIT-LT1 transfection reagent (Mirus) according to manufacturer's instructions. 5h post transfection, cells were washed twice with PBS and 2.5 mL of OptiMEM – 1 % pen/strep was added. The supernatant containing BlaM1 VLPs was harvested 72 h post transfection and subjected to centrifugation to remove cell debris. Aliquots were stored at -80 °C. WSN-mNeonGreenM1 VLPs were generated using the same protocol but replacing pCAGGS.WSN-BlaM1 with pCAGGS.WSN-mNeonGreenM1.

VLP entry assay and intracellular β-lactamase detection

Aliquots of VLP were thawed on ice. VLPs were diluted in fresh OptiMEM media to a ratio of 1:5 or 1:2 for subsequent infection of 293T or CHO cells respectively. First, VLPs were treated with 5 µg/mL of TPCK-trypsin for 20 min at 37 °C in order to activate the HA at the surface of the VLPs. The TPCK-trypsin was inactivated using 10 µg/mL of trypsin inhibitor from Glycine max (Soybean) (Sigma #T6414) for 20 min at 37 °C. Then 1 % DEAE was added and ammonium

chloride was added to a final concentration of 20 mM if needed. Prior to infection, the target cells (293T or CHO approximately 80 % confluent) were washed twice with PBS. For a 24-well plate, 400 µL of conditioned VLPs were added per wells. Cells were incubated 3 h at 37 °C. Following infection, cells were detached by pipetting and centrifuged for 5 min at 300 x g. Cells were washed once in PBS. Supernatant was removed and cells were resuspended in 100 µL OptiMEM. Then, 20 µL of Alternative Substrate Loading Solution (ASLS) were added. The ASLS was prepared according to manufacturer instructions, as follow for the equivalent of a well of 24-well plate: 0.25 µL CCF2-AM, 1.2 µL solution B, 17.95 µL solution C and 0.6 µL solution D. Loading of CCF2-AM into cells was done for 30 min at 37 °C. Then, cells were centrifuged for 5 min at 1200 rpm, resuspended in PBS and analyzed at the UNIGE Flow Cytometry facility on a machine name typically recording 10,000 events. Samples were gated on live cells and analyzed for the cleavage of CCF2-AM using FlowJo 10.7.1 software. The gating strategy is visualized in **Supplementary Fig. 15**.

LDL uptake assay

Cells were incubated with LDLDyLightTM 550 (Abcam) for four hours and LDL uptake was visualized by standard fluorescence microscopy, comparing to untreated cells. Cell density was monitored by brightfield imaging.

Flow cytometry

For detection of TfR1 on the cell surface after ferristatin II treatment, A549 cells were seeded in 12 well plates at a subconfluent density (80%). The cells were incubated for 4h with 1:1000 DMSO and 50um of Ferristatin II. After the incubation, cells were washed with PBS, detached using trypsin and centrifuged at 300g for 10 mins at 4°C. The supernatant was removed and

the cells were washed once with PBS 1% BSA 0.01% NaN3. The cells were centrifuged at 300g for 10 mins at 4°C and the supernatant was removed. The cell pellets were stained with CD71 Mouse anti-Human, PE (BD Biosciences #561938) at 1:250 for 1h at 4°C in dark. After the staining, the cell pellets were washed twice with PBS 1% BSA 0.01% NaN3 followed by fixing with PBS 2% formaldehyde for 20 mins at 4°C. The cells were centrifuged at 300g for 10 mins and the supernatant removed. The cells resuspended in PBS and analysed at the UNIGE Flow Cytometry facility on a Beckman Coulter Cytoflex typically recording 10,000 events. Samples were gated on live cells and analysed for the surface expression of PE using FlowJo 10.7.1 software.

For uptake of fluorescent beads cells, A549 and CHO cells were seeded in 12 well plates at 80 % density. In line with the IAV infection experiments the cells were incubated with 10 or 1000 yellow-green FluoSpheres (Thermofisher #F8848)/cell. Beads were incubated for 45 min or 5 h on the cells. After the incubation, the cells were washed three times with PBS, detached using trypsin and centrifuged at 1500rpm for 5 mins. The supernatant was removed and the cells were resuspended in PBS and analysed at the CMU Flow Cytometry facility on a Beckman Coulter Cytoflex typically recording 10,000 events. Samples were gated for live cells and analysed for the expression of FITC using FlowJo 10.7.1 software. The gating strategies visualized in **Supplementary Fig. 19-21**.

Purified proteins

Trimeric HA of A/California/04/2009 was previously described(12). Based on this sequence we added the coding sequence for HRP on the 3’ end of the T4foldon, connected via a GSGSG-linker and followed by a His10 tag (sequence provided in **Supplementary Fig. 22-24**). The Y98F mutant was derived by targeted mutagenesis. A trimerized HRP control was designed with

the same T4foldon and His10 tag. The CDS for a recombinant TfR1 ectodomain was previously published(13). All constructs were synthesized by GeneArt and provided in a pFastBac1 vector for production of baculoviruses at the protein core facility of the CMU.

Briefly, recombinant secreted proteins were expressed in baculovirus infected Sf9 insect cells. Baculovirus were generated using a modified pFastBac vector encoding C-terminally tagged i) WT Cal9, ii) mutated Cal9 or iii) no protein. All three proteins had a C-terminal tag composed of fused HA epitope - HRP and a 10-histidine tag. The gene encoding the transferrin receptor was placed following a gp67 secretion signal. All proteins were purified following a similar protocol.

Media containing the proteins of interest was clarified by centrifugation at 4000 g for 15 min at 4 °C and filtered using 0.22 micrometer filters. Media was concentrated to 50 ml final, adjusted to 10 mM imidazole concentration and applied to a 5 ml His-trap FF column (Cytiva). Column was washed with 100 ml of PBS supplemented with 1 M NaCl and 10 mM Imidazole and eluted with 15 ml of elution buffer (1 x PBS, 200 mM NaCl, 450 mM imidazole). Eluted protein was concentrated to 1 ml using AMICON 30 MWCO concentrators and loaded on a Size Exclusion Chromatography Superdex 200 10/300 column at 4 °C equilibrated in PBS. Fractions containing pure protein were pooled, concentrated and flash frozen in liquid nitrogen.

TfR1-HA pull down in 293T

Subconfluent 293T cells were transfected with either 1 µg of pDisplay.Flag-TfR1ecto or 250 ng of pCAGGS.Flag-ZsGreen as indicated. 48 h post transfection, cells were washed twice and lysed in 500 µL of IP Lysis buffer (50 mM Tris HCl pH 7.5, 150 mM NaCl, 0.5 % v/v NP40, 5 mM

EDTA, with protease inhibitors). The lysates were incubated on ice for 15 min, sonicated twice for 10 sec using microtip, output 6-9W and cleared by centrifugation at 10000 xg at 4 °C for 20 min. Whole cell lysates were prepared by mixing 50 µL of lusate with 50 µL of 2X Protein Lysis buffer (Tris-glycerol-SDS-DTT). The anti-FLAG M1 agarose affinity gel (Sigma # A4596) slurry (30 µL per condition) was washed thrice with IP lysis buffer and resuspended in 600 µL of IP lysis buffer (per condition). For immunoprecipitation, 400 µL of lysate and 600 µL of beads in buffer were incubated over night at 4 °C on a rotating wheel. The next day, beads were washer 3 times with IP lysis buffer without protease inhibitors, resuspended in 500 ul IP lysis buffer with protease inhibitor and 1 µg of either HA wt or Tf was added as indicated. Tubes were incubated over night at 4 °C on a rotating wheel. The next day, beads were washer 3 times with IP lysis buffer without protease inhibitors, all buffer was removed and and 50 µL of 1X Protein Lysis buffer (0.05M Tris, pH6.8, 10%v/v glycerol, 0.05M DTT, 2% w/v SDS) added. Samples were boiled at 95 °C for 5 min, spun at 10000 xg for 10 min and 15 ul were loaded on a SDS-PAGE and western blotting analysis.

TIRF microscopy

Microscopic analysis of interaction between VLP and TfR1-BFP or BFP expressing CHO cells was performed on a Nikon Eclipse Ti with perfect focus system with a 100x/1.49 NA oil immersion objective and a Hamamatsu Orca-Fusion C15440-20UP CMOS camera. Cells were cultured on glass-bottom petri dishes for 4 h before moving them to the microscope. Cells were incubated with undiluted VLP while being on the microscopic stage and imaging started around 15 min after addition of VLP. Cells and VLP were imaged in the respective fluorescent channel in total internal reflection fluorescence (TIRF) illumination mode limiting the observation depth in axial direction to around 100 nm. VLP were additionally imaged with

epifluorescent illumination to observe VLP after leaving the TIRF plane (i.e., after internalization; see also **Fig. 5I**). Fluorescent movies were acquired with 1 frame/min. Cells showed no noticeable retraction within the imaging period indicating low levels of phototoxicity.

TIRF data analysis

VLP were tracked with the Fiji software package (14) and the plugin TrackMate (15). Before tracking, movies were drift corrected with the 'Descriptor-based registration' plugin based on VLP that adsorbed to the glass surface around cells and that did not move during the time course of the movie. Tracking was performed on VLP imaged with TIRF in TrackMate. Only tracks with the following conditions were considered for further analysis: 1) following definition of the cell edges, we used the LoG Detector with an estimated object diameter of 6 pixels, 2) the initial threshold was left untouched while filters on spots was adjusted depending on the experimental background, 3) then as a tracker, we used the simple LAP tracker with a linking max distance and a gap-closing distance of 10 pixel, the gap-closing max frame gap was set to 2, 4) all tracks shorter than 3 frames were filtered out, 5) the tracks and branch hierarchy analisis were export as .cvs files for further analysis. Intensity of TfR-BFP or surface expressed BFP was corrected for bleaching by measuring TfR intensity at the beginning and at the end of movies and by correcting for the loss in fluorescence in all time frames.

TIRF Software availability

Relevant data was extracted from TrackMate-exported files via custom-written code in a Jupyter notebook. The notebook is available from https://github.com/Mitchzw/viral-tracking

2-step-Enzyme-linked lectin assay

Recombinant N-terminal human IgG1 Fc-tagged transferrin (Cayman, produced in HEK293T cells) was coated o/n at 4°C onto Nunc MaxiSorb plates (1µg/ml, 100µl/well). All washing steps were done using 200µl of buffer. Plates were washed 3x with PBS + 1% BSA. PNGase (or PBS) was added (1U/well) and incubated at 37°C for 45min. Plates were washed 3x with PBS + 1% BSA and blocked for 1h at 37°C with PBS + 1% BSA. Plates were washed 1x with PBS + 1% BSA. 5x 10e8 pfu of PR8 was added per well or an equivalent dilution of allantoic fluid in PBS in presence or absence of 0.4ng/ml of holotransferin (Sigma). Plates were incubated 2h at 4°C. Plates were washed 3x with PBS + 1% BSA and 3x with PBS.Fetuin was added at 5 µg/ml (100µl/well) for 2h at 37°C. Supernatants were transferred to a new plate and incubated o/n at 4°C. Plates were washed with PBS (3x), blocked 1h at RT with 200 µl PBS-5% BSA, washed 3x PBS 0.5% Tween-20 (200µl) and once with 1x PBS. Wells were incubated with 100 µl Po-PNA at 5 µg/ml in PBS at RT for 2h in the dark. Plates were washed 6x with with PBS 0.5% Tween-20 and 1x with PBS. 100µl of TMB Sure Slow substrate were added per well and incubated for 5-10min at RT in the dark. The reaction was stopped by adding 50µl of 2 M sulfuric acid and the plates were read at 450 nm.

***In vivo* infection experiments**

C57BL/6J mice (female, 8 weeks of age) were purchased from Charles River Laboratories (France) and housed under SPF/BSL2 conditions. All animals were housed for 7 days to adjust to housing conditions under a strict 12h light/dark cycle and fed ad libitum. On the three days preceding infection, mice were injected twice a day intraperitoneally with 40mg/kg of ferristatin II(16) or an equivalent volume of DMSO in PBS. On the day of IAV infection, mice

were injected intra peritoneally with a mix of ketamin/xylazine (100 and 5 mg/kg, respectively) in 200 µl of sterile PBS. Upon reaching deep anesthesia, mice were inoculated intra-nasally with 40 µl of PBS or with 40 µl of PBS containing 40 pfu of VN1203. Animal weights were measured daily starting on the day of the first CB injection. Animals were euthanized using controlled CO2 exposure at 2, 4 and 6 d post infection. Whole lungs were sampled immediately after euthanasia using sterile tools. Tools were changed in between experimental groups to avoid cross-contamination. Whole lungs were stored at -80 °C until being processed. Whole lungs were homogenized with 1/4'' stainless steel grinding balls (MPBio, USA) in 1 ml PBS, using a Bead Blaster 24 (Benchmark Scientific, USA) with a speed setting of 6 m/s for 30s and 30 s intervals, repeated 2 times. Samples were centrifuged at 2000 x g for 5 min and supernatant was used for plaque assay in MDCK cells.

References

1. D. H. Persing, Diagnostic molecular microbiology. Current challenges and future directions. *Diagn Microbiol Infect Dis* **16**, 159-163 (1993).
2. M. Quinlivan *et al.*, Attenuation of equine influenza viruses through truncations of the NS1 protein. *J Virol* **79**, 8431-8439 (2005).
3. S. Anchisi, A. R. Goncalves, B. Mazel-Sanchez, S. Cordey, M. Schmolke, Influenza A Virus Genetic Tools: From Clinical Sample to Molecular Clone. *Methods Mol Biol* **1836**, 33-58 (2018).
4. H. Niwa, K. Yamamura, J. Miyazaki, Efficient selection for high-expression transfectants with a novel eukaryotic vector. *Gene* **108**, 193-199 (1991).
5. F. A. Ran *et al.*, Genome engineering using the CRISPR-Cas9 system. *Nat Protoc* **8**, 2281-2308 (2013).
6. K. Labun *et al.*, CHOPCHOP v3: expanding the CRISPR web toolbox beyond genome editing. *Nucleic Acids Res* **47**, W171-W174 (2019).
7. L. Strahle, D. Garcin, P. Le Mercier, J. F. Schlaak, D. Kolakofsky, Sendai virus targets inflammatory responses, as well as the interferon-induced antiviral state, in a multifaceted manner. *J Virol* **77**, 7903-7913 (2003).
8. D. Ostertag, T. M. Hoblitzell-Ostertag, J. Perrault, Overproduction of double-stranded RNA in vesicular stomatitis virus-infected cells activates a constitutive cell-type-specific antiviral response. *J Virol* **81**, 503-513 (2007).
9. D. Bausch-Fluck *et al.*, A mass spectrometric-derived cell surface protein atlas. *PLoS One* **10**, e0121314 (2015).

10. B. Mazel-Sanchez, I. Boal-Carvalho, F. Silva, R. Dijkman, M. Schmolke, H5N1 Influenza A Virus PB1-F2 Relieves HAX-1-Mediated Restriction of Avian Virus Polymerase PA in Human Lung Cells. *J Virol* **92** (2018).
11. S. Pleschka *et al.*, A plasmid-based reverse genetics system for influenza A virus. *J Virol* **70**, 4188-4192 (1996).
12. J. Stevens *et al.*, Structure of the uncleaved human H1 hemagglutinin from the extinct 1918 influenza virus. *Science* **303**, 1866-1870 (2004).
13. J. Gruszczyk *et al.*, Transferrin receptor 1 is a reticulocyte-specific receptor for Plasmodium vivax. *Science* **359**, 48-55 (2018).
14. J. Schindelin, C. T. Rueden, M. C. Hiner, K. W. Eliceiri, The ImageJ ecosystem: An open platform for biomedical image analysis. *Mol Reprod Dev* **82**, 518-529 (2015).
15. D. Ershov *et al.*, Bringing TrackMate into the era of machine-learning and deep-learning. *bioRxiv* 10.1101/2021.09.03.458852, 2021.2009.2003.458852 (2021).
16. S. L. Byrne *et al.*, Ferristatin II promotes degradation of transferrin receptor-1 in vitro and in vivo. *PLoS One* **8**, e70199 (2013).

Supplementary Figure legends:

Supplementary Figure 1: A) Graphical summary of the CSPL technique. The graphic was generated with BioRender.com. **B)** Representative SDS-PAGE of insect cells expresses HRP, HA wt HRP or HA Y98F HRP. 5µl of purified protein containing fractions were loaded onto a 10% SDS-polyacrylamide gel. The gel was stained with Coomassie Blue after electrophoretic separation. **C)** HRP activity of insect cell produced proteins. Processing of the TMB substrate was measured at 450nm and plotted against the concentration of the provided protein. Measures were performed in triplicates in two independent experiments.

Supplementary Figure 2: Cell surface proximity ligation assay. A) A549 cells after cell surface proximity ligation. Cells were permeabilized and stained with anti CD44 (epithelial cell surface marker, red), streptavidin-ALEXA488 (green) and DAPI (blue). Merged images for all three channels are shown for representative cells (from n=3-6 per condition). **B)** Streptavidin-

pulldown of biotinylated host cell proteins after CSPL. Biotinylated proteins were probed with streptavidin-HRP.

Supplementary Figure 3: A) Heat map of number of peptides found in mass spectrometry Run 1 and Run 2 for the three experimental conditions. **B)** Venn diagram depicting the number of proteins found in each of the three mass spectrometry experiments and their respective overlap **C)** Hits (indicated by Gene ID) from three mass spectrometry experiments organized according to the Venn diagram sections in C.

Supplementary Figure 4: A) List of proteins used for the STRING analysis. Left two columns indicate hits found in at least two out of three mass spectrometry experiments, the right two columns indicate "background" proteins found in only one of the three experiments.
B-C) String network of hits **(B)** and background proteins (**C**) and top five biological processes and molecular function according to gene ontology classification and sorted by lowest to highest false discovery rate (FDR). In **(B and C)**, proteins affiliated with the biological process "entry into host" and molecular function "virus receptor activity" are labeled in blue and/or red, respectively.

Supplementary Figure 5: Minigenome assay for the viral polymerase complex of PR8 **(A)** and VN **(B)** in A549 CTRL or TFRC KO cells. Viral polymerase driven firefly luciferase activity was normalized to the activity of constitutively expressed Renilla luciferase and normalized to the mean activity in control cells (100%). The line indicates median polymerase activity, each dot represents a measurement from two independent experiments performed with three

technical replicates per clone (n=4 clonal cell lines). *P*-values were determined using the Mann Whitney test.

Supplementary Figure 6: A) A549ctrl and TFRC KO were infected with 5 MOI of SeV-GFP. Total cell lysates were separated by SDS-PAGE and analyzed by western blot for SeV N and TfR1 levels. Equal loading was confirmed by probing for beta actin. A representative blot of 3 independent experiments is shown. **B**) Quantification of SeV N levels from four independent infection experiments as shown in (E). Each symbol refers to one independent experiment, performed with one A549 CTRL and one A549 TFRC KO clone, respectively. Statistical significance was determined by a Mann-Whitney test. *p*-values are indicated. **C)** Multi-cycle growth curve of SeV-GFP in A549 CTRL or A549 TFRC KO cells. Cells were infected with 0.01 MOI of SeV-GFP. Relative counts of GFP positive cells were determined by FACS (10^4 cells single cells were analysed/sample). Mean values +- SD of two independent experiments with two biological replicates each are indicated. Statistical significance was determined by multiple Wilcoxon test. *P*-values are indicated.

Supplementary Figure 7: A) Calibration curve for M1 specific qPCR. pDZ-M1 plasmid was diluted in 50x steps and the respective copy number was calculated. For each dilution qPCR values using M1 specific primers are indicated as circles. Measurements were performed as technical triplicates. Linear regression curves were plotted and the R square is indicated. **B-C)** A549 ctrl and TFRC KO (**B**) or CHO Lec1 ctrl and CHO Lec1 TFRC OE (**C**) were incubated on ice with the indicated MOI of PR8. Non attached virus was removed by washing and attached virus was quantified by RT-qPCR from total RNA. Each dot represents a biological replicate. For (**A**) a representative assay of two independent assays with a different A549 TFRC KO vs

A549 CTRL clone combination is shown. Lines indicate median copy numbers as compared to the calibration curve in **(A)**. *p*-Values were determined by one way ANOVA.

Supplementary Figure 8: LDL DyLight550 entry into A549 ctrl and A549 TFRC KO (**A**) or CHO Lec 1 ctrl or CHO Lec 1 TFRC OE cells (**B**) was visualized by microscopy. Cells were incubated for 4h ours with LDL DyLight550, which was detected in the red channel. Total cells were monitored in a brightfield.

Supplementary Figure 9: Latex bead entry into A549 ctrl and A549 TFRC KO cells. Cells were incubated with 1000 fluorescent beads/cell for 45min (**A**) or 5h (**B**), washed three times and analysed by FACS. Percentage of positive cells (left panel) or mean fluorescent intensity per cell (right panel are indicated for three independent experiments). Statistical significance was determined with one way ANOVA. *P*-values are indicated.

Supplementary Figure 10: Latex bead entry into CHO PRO5 ctrl and CHO PRO 5TFRC OE cells. Cells were incubated with 1000 fluorescent beads/cell for 45min (**A**) or 5h (**B**), washed three times and analysed by FACS. Percentage of positive cells (left panel) or mean fluorescent intensity per cell (right panel are indicated for three independent experiments). Statistical significance was determined with one way ANOVA. *P*-values are indicated.

Supplementary Figure 11: A-B) Cytotoxicity of Ferristatin II. A549 cells were incubated with the indicated concentrations of ferristatin II and LDH release was measured after 4h of ferristatin II preincubation. Six technical repeats from one experiment are shown (**A**). Intracellular ATP levels were measured after 4h of ferristatin II preincubation (for early

replication experiments, 3 technical repeats from one experiment are shown) (**B**) or 4h and 24/48h of ferristatin II treatment (for multicyle growth curves, 5 technical repeats from one experiment are shown). Measures were done in duplicates in two independent experiments. Statistical significance was determined by multiple t-tests. *p*-values are indicated.

Supplementary Figure 12: Human skin fibroblasts were infected with VSV (**A**) or SeV (**B**). Control fibroblast, C, or patient fibroblasts, P, with a homozygous Y20H substitution in TfR1 were infected with 5 MOI of virus for the indicated times. Total cell lysates were separated by SDS-PAGE and analysed by Western blot probing for virus antigen (VSV-G or SeV N) and TfR1. Equal loading was confirmed by probing for beta actin. Representative blots of three independent experiments are shown. Quantification of the NP signal is depicted below. Statistical significance was determined by one way ANOVA. *P*-values are indicated.

Supplementary Figure 13: TfR1 purified from 293T cells does not coimmuneprecipitate with purified recombinant influenza HA, independent of the presence of holotransferrin (Tf). 1µg of each of the indicated proteins was co-incubated and TfR1 was pulled down with anti-Flag beads. Coprecipitated proteins were separated by SDS-PAGE and probed with specific antibodies.

Supplementary Figure 14: Colocalization of mNeonGreenM1 VLPs with TfR1-BFP. TfR1-BFP signal at the position of the VLP was quantified for the enveloped VLPs on the cell surface. Only tracks longer than 6 frames were considered. Tracks which resulted in entry before frame 30 (entry, n=199) were compared to those, which did not enter until frame 30 (no entry, n=113). Only the first three and the last three frames of each track were considered.

Each dot represents an individual data point from the tracks. Tracks were recorded from three cells in three independent experiments. *p*-values were calculated with one way ANOVA.

Supplementary Figure 15: Chemical targeting of TfR1 suppresses IAV entry. A) Left panel: A549 cells were treated for 4h with 50µM of ferristatin II or an equivalent amount of DMSO. Cells were infected for 3h with 5 MOI of the indicated IAV strains. Total cell lysates were separated by SDS-PAGE and analyzed by western blot for NP and TfR1 levels. Equal loading was confirmed by probing for beta actin. A representative blot of four independent experiments is shown. **Right panel:** A549 were infected with a clinical H3N2 IAV isolate (5MOI for 6h). Total cell lysates were separated by SDS-PAGE and analyzed by western blot for NP and TfR1 levels. Equal loading was confirmed by probing for beta actin. A representative blot of two independent experiments is shown. **B)** Quantification of NP band intensity as depicted in (**A**). Lines indicate the respective experimental pairs of DMSO control and ferristatin II treated samples. Statistical significance was determined by paired t-test. *p*-values are indicated. **C)** A549 were treated as in (**A**) and infected with indicated IAV strains at an MOI of 0.01. Infectious viral particles were quantified by standard plaque assay on MDCK cells. Three independent experiments with biological duplicates were analyzed. Statistical significance was determined by unpaired t-test. *p*-values are indicated. **D)** PCLS from two human patients were infected with 10^5 pfu of A/Wyoming/03/2003 (H3N2) after DMSO (empty circle) or ferristatin II treatment (black square). Supernatants were collected at the indicated time points. Viral titers were determined by standard plaque assay. Median pfu/ml are indicated from 6 PCLS per patient infected in two independent experiments with three biological replicates. Statistical significance was calculated with multiple Mann Whitney tests. p-values are indicated.

Supplementary Figure 16: A) Surface TfR1 levels as determined by FACS. A549 cells were treated with 50µM ferristatin II or equivalent amounts of DMSO and analysed by FACS. Values are normalized to the mean fluorescence intensity of surface TfR1 staining of A549 ctrl. Gating strategy is plotted on the right hand side Measures were performed in duplicates in two independent experiments. *p*-values were calculated by one way ANOVA. **B**) Total TfR1 levels in A549 after 8h of ferristatin treatment as determined by WB. Equal loading was insured by probing for beta actin. A representative blot is shown. **C**) 293T cells were treated as in (**A**) and infected with BlaM1-VLPs displaying the HA and NA of WSN or no envelope. Ammonium chloride was used to inhibit endosomal entry. Only cells with substrate conversion (BlaM1 positive) are depicted. (n=2 with biological duplicates). Statistical significance was determined by two way ANOVA. Adjusted *p*-values are indicated. **D-E**) A549 cells treated with 50µM of ferristatin II for 4 were infected with 5 MOI of VSV-GFP or SeV-GFP and cells were lysed at the indicated time points. Viral replication was approximated by blotting for VSV-G (**D**) or SeV N (**E**). Representative blots of 2 independent experiments for the VSV-GFP infection and 3 independent experiments for the SeV-GFP infection are shown. **F**) Quantifications of the viral protein levels from panel D and E signal. Statistical significance was determined by one way ANOVA. *P*-values are indicated. **G**) A549 ctrl or TFRC KO cells were incubated for 4h with ferristatin II or DMSO and infected with 10 MOI of PR8 for 4h. NP bands were quantified and normalized to actin. Each dot represents an individual measurement from three independent experiments with four cell clones. Normalized NP band intensity was set to 1 for A549 ctrl cells treated with DMSO. The line indicates mean value. *p*-values were determined with one way ANOVA. **H)** VLP entry assay using VSV-G pseudotyped replication deficient HIV VLPs encoding Gaussia luciferase. Median nfold relative light units normalized to non-envelopped

VLP for DMSO treated and ferristatin II treated A549 cells are indicated for three independent experiments. Statistical significance was determined by a paired t-test. *p*-values are indicated. **I)** Multicycle growth curve of VSV-GFP in presence or absence of ferristatin II. Median viral titers (pfu/ml) of four biological replicates from two independent experiments are indicated. Statistical significance was determined by a one way ANOVA test. *p*-values are indicated. **J)** Multi-cycle growth curve of SeV-GFP in A549 in presence or absence of ferristatin II. A549 cells were infected with an MOI of 0.5 for indicated time points. Relative counts of GFP positive cells were determined by FACS. Median values of two independent experiments with two biological replicates each are indicated. Statistical significance was determined by multiple t-tests. *p*-values are indicated.

Supplementary Figure 17: A) Normalized body weights from 30 animals treated twice a day for three days with 40mg/kg ferristatin II or DMSO. On day 4 mice were infected and 2, 4 and 6 days post infection 10 animals were sacrificed for viral lung titers. The graph combines data from two independent experiments with 5 animals per group each. Mean relative body weight normalized to initial body weight is depicted. No significant difference was determined by two way ANOVA and multiple comparisons of the two groups at every time point. **I)** C57BL6/J mice were injected twice daily with 40mg/kg ferristatin II (i.p.). Mice were infected with 40pfu of VN1203. Viral lung titers from n=8-10 animals/group were determined at indicated time points from total lung homogenates by standard plaque assay on MDCK cells. Statistical significance was determined by two way ANOVA. *p*-values are indicated.

Supplementary Figure 18: Revolving door model. IAV exploits TfR1 recycling either by direct binding to its glycan modifications (cis) or by binding to a near-by glycoprotein (trans). Virions

enter with TfR1 into the endosome via clathrin mediated endocytosis. After pH induced viral and host membrane fusion, TfR1 recycles back to the cell surface. To simplify we depicted only the endocytosis for the entry in trans. Image was generated with Biorender.

Supplementary Figure 19: Gating strategy for the TfR1 cell surface staining.

Supplementary Figure 20: Gating strategy for the BlaM1 assays

Supplementary Figure 21: Gating strategy for the latex bead uptake assay for A549 (A) or CHO cells (B).

Supplementary Figure 22: recombinant trimeric HRP construct for expression in insect cells.

Supplementary Figure 23: recombinant trimeric HA-WT-HRP construct for expression in insect cells.

Supplementary Figure 24: recombinant trimeric HA-Y98F-HRP construct for expression in insect cells.

Supplemental table 1: patient information for donors of lung tissue samples used for the generation of PCLS

Movie S1: Video corresponding to **Fig. 5I**

Dataset S1: Raw identified peptide counts of the prerun experiment for the three molecular probes are indicated in column E-G.

Dataset S2: Filtered peptide counts of the prerun experiment for the three molecular probes are indicated in column E-G.

Dataset S3: Raw identified peptide counts of the run 1 experiment for the three molecular probes are indicated in column E-G.

Dataset S4: Filtered peptide counts of the run 1 experiment for the three molecular probes are indicated in column E-G.

Dataset S5: Raw identified peptide counts of the run 2 experiment for the three molecular probes are indicated in column E-G.

Dataset S6: Filtered peptide counts of the run 2 experiment for the three molecular probes are indicated in column E-G.

Dataset S7: Overlap of filtered peptide sets from run 1 and run 2.

Supplemental table 1: PCLS donor information

Patient	Gender	Age (y)	Tumor type	History	Smoker
N°1	F	56	Adenocarcinoma	Renal cell cancer	Yes
N°2	M	70	Adenocarcinoma	Chronic infection with *Streptococcus mitis*; hypertension, atrial fibrillation	Yes

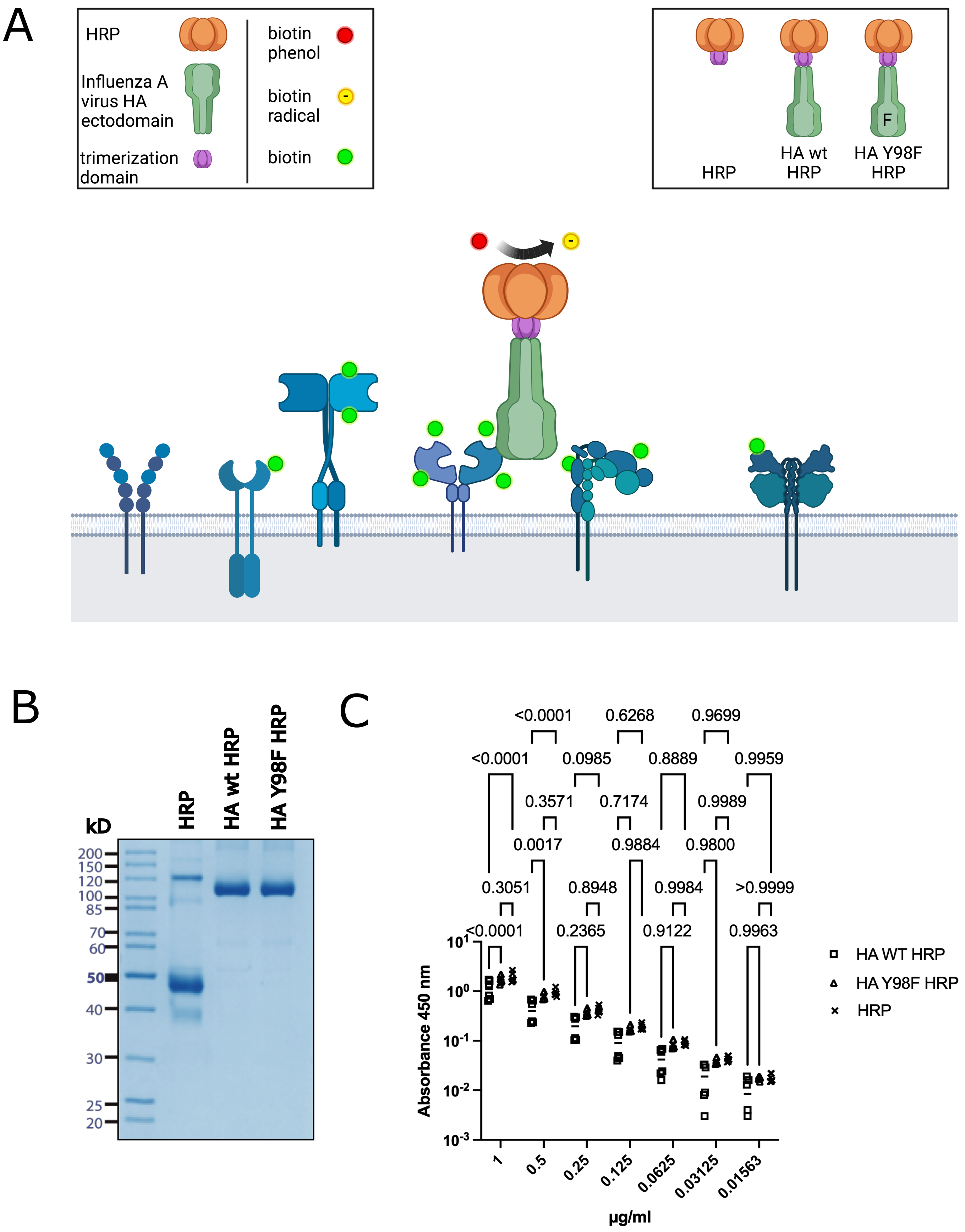

Supplementary Figure 1

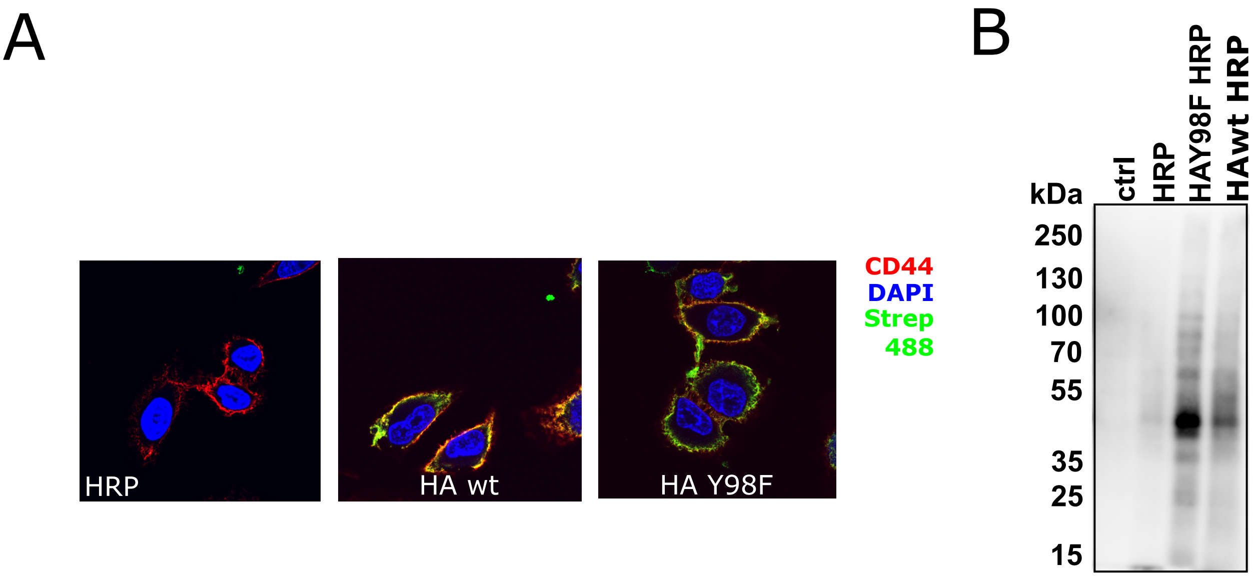

Supplementary Figure 2

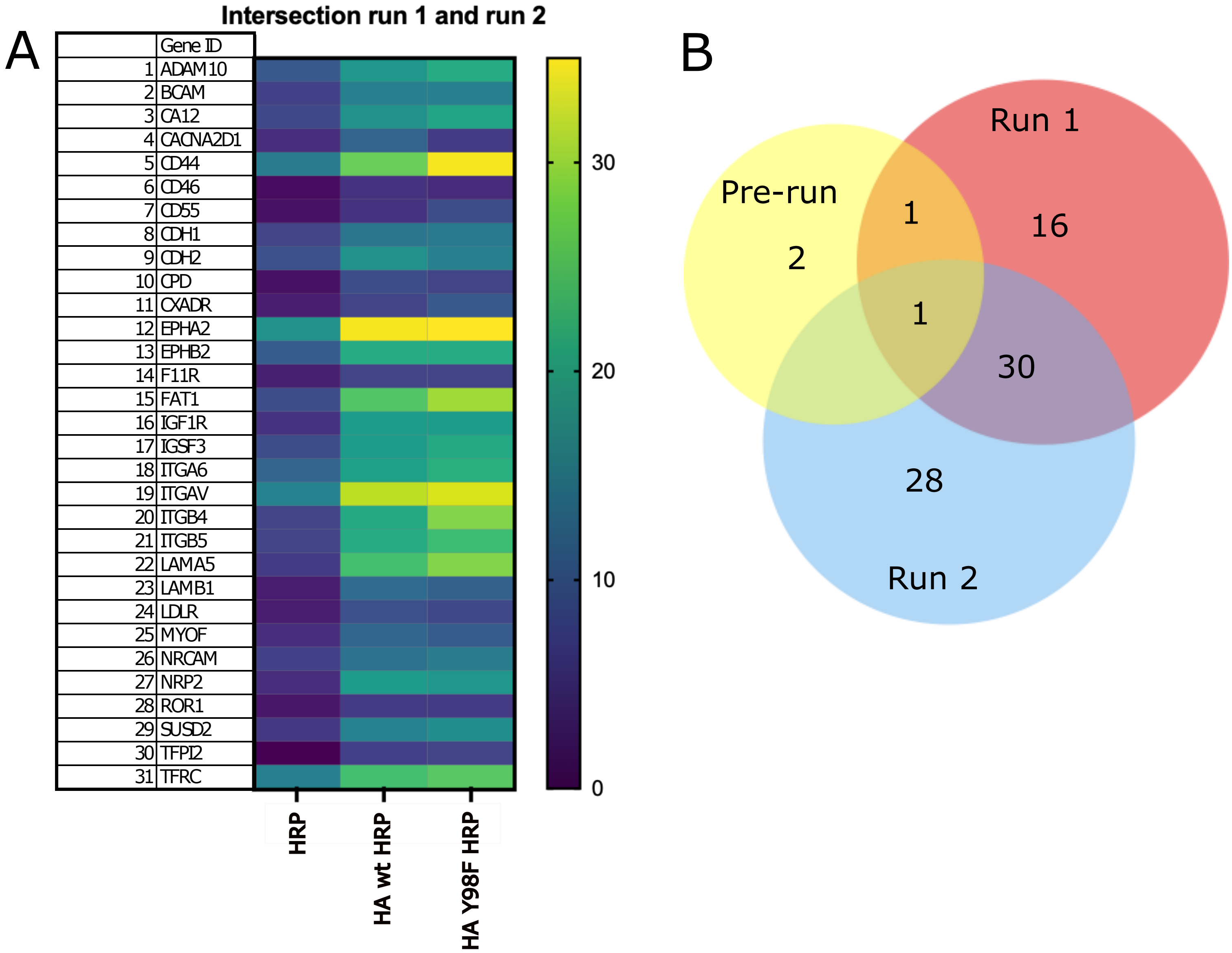

C

Experiment	Hits, (gene, name)
All runs	TFRC
Pre-run, Run, 1	ITGB1
Run, 1, Run, 2	CD44, NRCAM, CDH1, LAMA5, LAMB1, EPHA2, CD46, CD55, ITGB5, CA12, MYOF, BCAM, CACNA2D1, ADAM10, ITGB4, IGF1R, TFPI2, LDLR, ITGA6, IGSF3, ROR1, SUSD2, CPD, EPHB2, CXADR, NRP2, ITGAV, CDH2, F11R, FAT1
Pre-run	CDSN, CP
Run, 1	ITGA3, MELTF, HSPG2, AGRN, CNTN1, CADM4, ROBO1, ALCAM, LAMC1, PLXNB2, ITGA2, EGFR, DCBLD2, CD109, PVR, NRP1
Run, 2	LTF, NEO1, LEPR, L1CAM, ITGA5, PTPRK, ANTXR1, PLXNA1, LRP1, LNPEP, ANPEP, THSD7A, PTPRF, PTGFRN, MRC2, PLXND1, MET, AXL, CRIM1, CD276, NTRK3, IGF2R, PTPRM, CADM1, ADAM17, ERBB2, CDH17, PLAUR

Supplementary Figure 3

A

hits		background		
ITGB5	ITGB1	AGRN	CD276	NRP1
LAMA5	ITGB4	ALCAM	CDH17	NTRK3
LAMB1	ADAM10	CADM4	CDSN	PLAUR
LDLR	BCAM	CD109	CP	PLXNA1
MYOF	CA12	CNTN1	CRIM1	PLXND1
NRCAM	CACNA2D1	DCBLD2	ERBB2	PTGFRN
NRP2	CD44	EGFR	HSPG2	PTPRF
CDH1	CD46	HSPG2	IGF2R	PTPRK
CDH2	CD55	ITGA2	ITGA3	PTPRM
CPD	ROR1	MELTF	ITGA5	ROBO1
CXADR	SUSD2	PLXNB2	L1CAM	THSD7A
EPHA2	TFPI2	PVR	LAMC1	AXL
EPHB2	TFRC	ADAM17	LEPR	CADM1
F11R	IGSF3	AGRN	LNPEP	CADM4
FAT1	ITGA6	ANPEP	LRP1	MET
IGF1R		ANTXR1	LTF	
ITGAV		MRC2	NEO1	

B

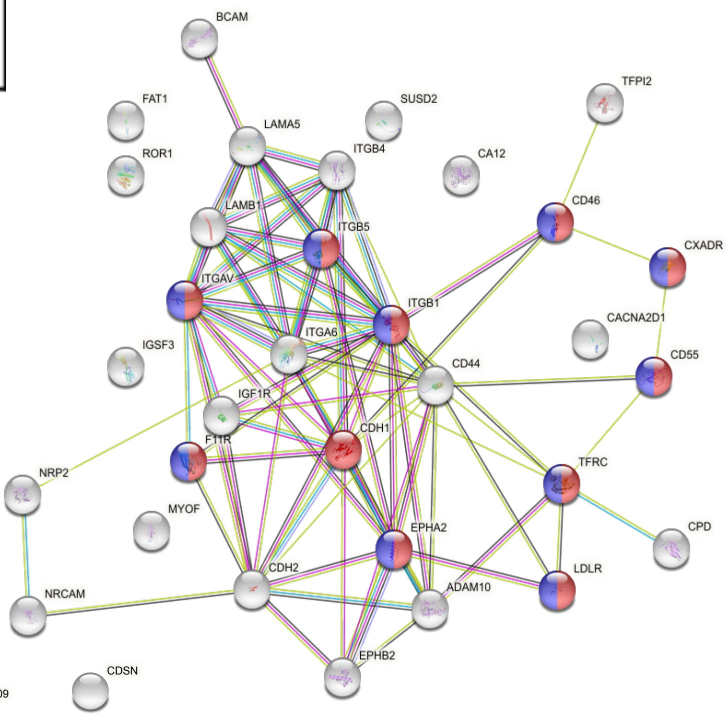

hits (biological process)				
GO term	description	count in netv	strength	FDR
GO:0044409	Entry into host	11 of 103	1.81	2.36E-13
GO:0007155	Cell adhesion	17 of 925	1.05	2.49E-11
GO:0098609	Cell-cell adhesion	13 of 505	1.2	1.07E-09
GO:0016477	Cell migration	15 of 896	1.01	3.51E-09
GO:0030198	Extracellular matrix organization	11 of 338	1.3	5.79E-09

hits (molecular function)				
GO term	description	count in netv	strength	FDR
GO:0001618	Virus receptor activity	10 of 74	1.92	1.72E-13
GO:0050839	Cell adhesion molecule binding	16 of 538	1.26	1.72E-13
GO:0005178	Integrin binding	9 of 147	1.57	1.97E-09
GO:0044877	Protein-containing complex binding	15 of 1216	0.88	1.14E-07
GO:0004714	Transmembrane receptor protein tyrosin	5 of 63	1.69	4.37E-05

C

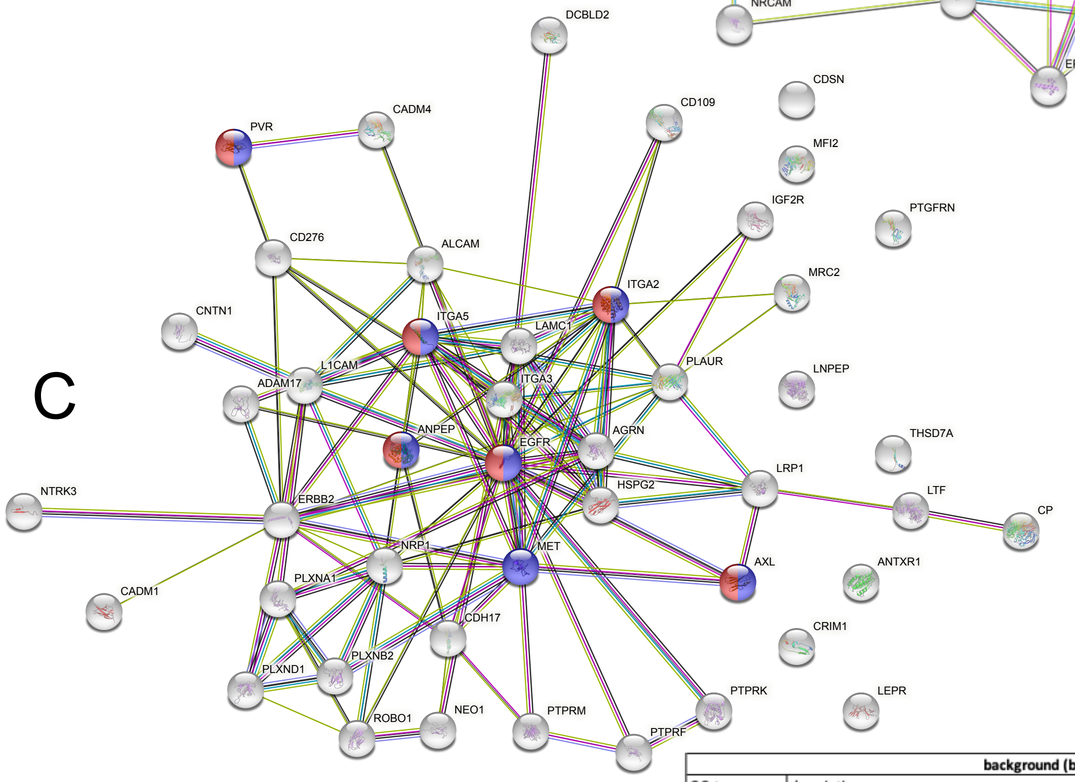

background (biological process)				
GO term	description	count in netv	strength	FDR
GO:0007155	Cell adhesion	23 of 925	1.02	1.55E-14
GO:0009653	Anatomical structure morphogenesis	30 of 2165	0.77	1.71E-14
GO:0040011	Locomotion	24 of 1251	0.91	1.67E-13
GO:0030154	Cell differentiation	35 of 3702	0.6	2.04E-13
GO:0000902	Cell morphogenesis	18 of 726	1.02	4.13E-11
GO:0044409	Entry into host	7 of 103	1.46	0.00000136

baclground (molecular function)				
GO term	description	count in netv	strength	FDR
GO:0038023	Signaling receptor activity	25 of 1453	0.86	3.34E-13
GO:0004714	Transmembrane receptor protein tyrosine kinase activity	8 of 63	1.73	4.96E-09
GO:0004888	Transmembrane signaling receptor activity	19 of 1240	0.81	1.22E-08
GO:0017154	Semaphorin receptor activity	5 of 12	2.25	0.000000185
GO:0001618	Virus receptor activity	6 of 74	1.54	0.0000127

Supplementary Figure 4

A

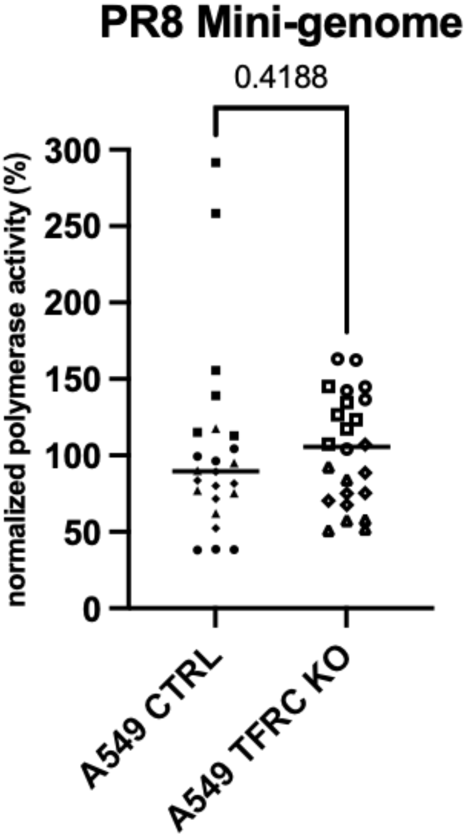

B

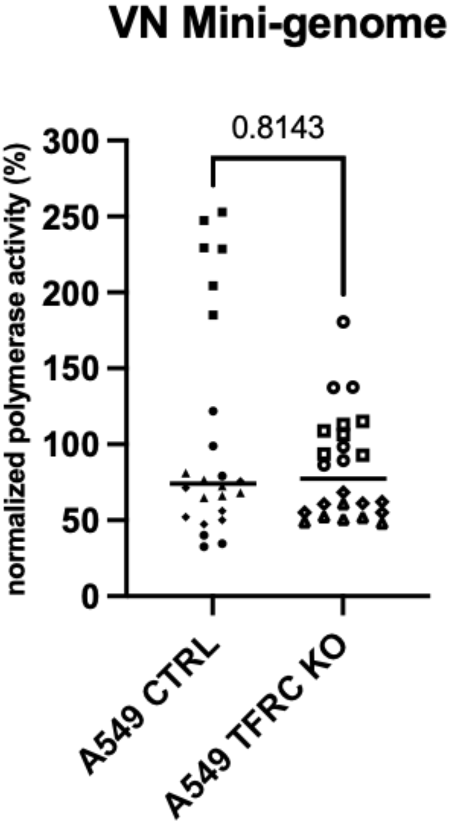

Supplementary Figure 5

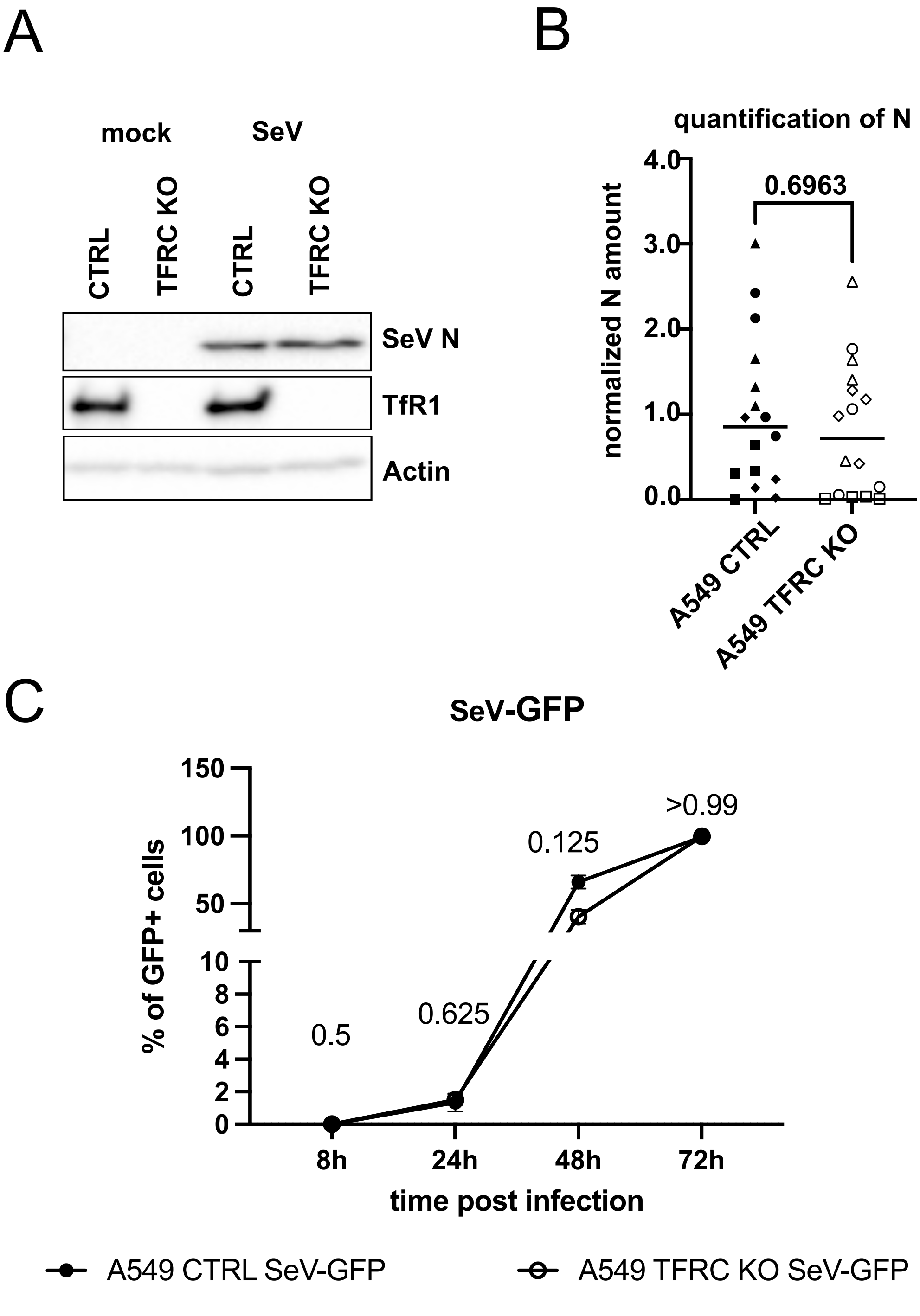

Supplementary Figure 6

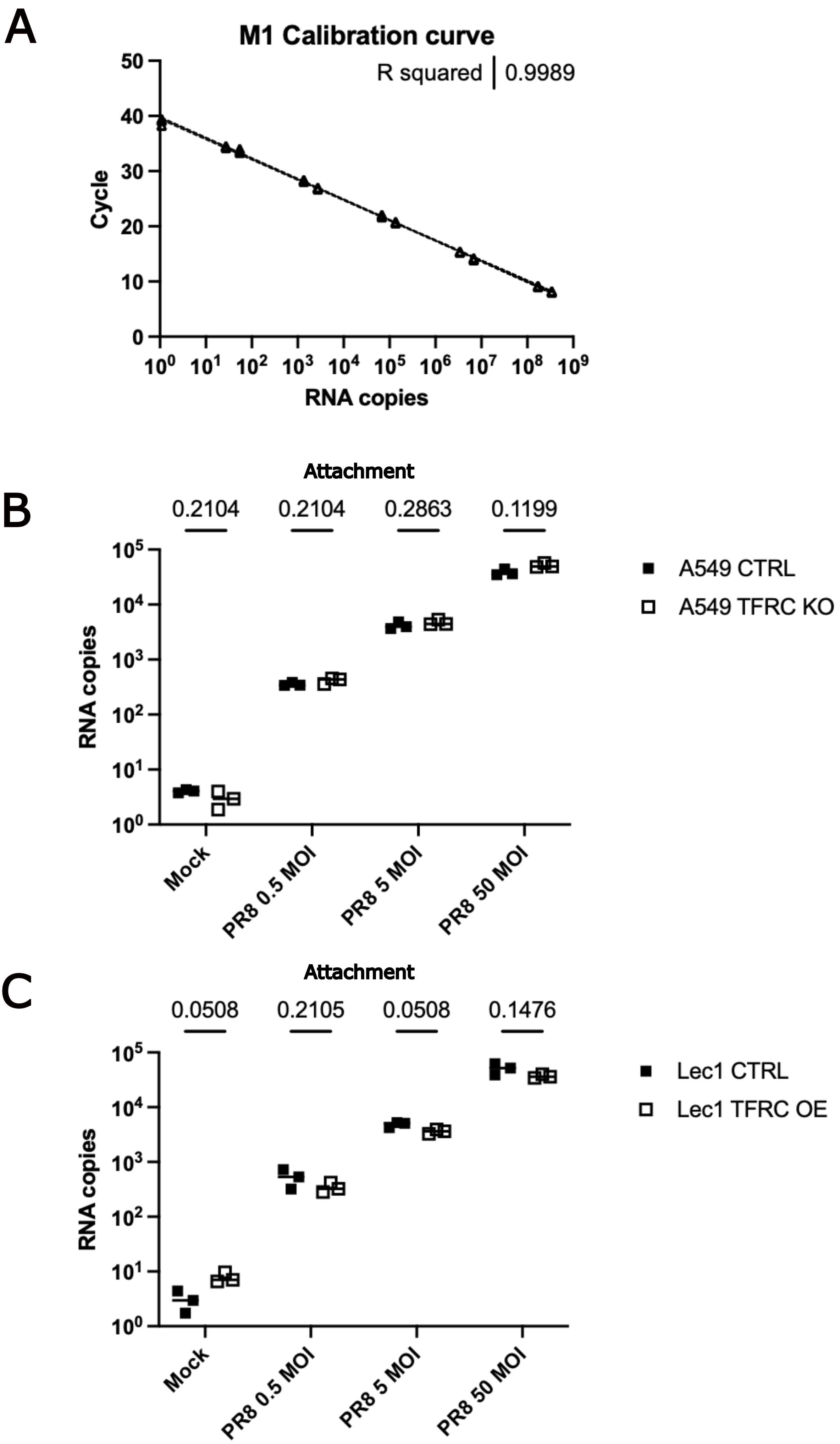
A
M1 Calibration curve
R squared | 0.9989
Cycle
RNA copies
Attachment
B
0.2104
0.2104
0.2863
0.1199
RNA copies
Mock
PR8 0.5 MOI
PR8 5 MOI
PR8 50 MOI
A549 CTRL
A549 TFRC KO
Attachment
C
0.0508
0.2105
0.0508
0.1476
RNA copies
Mock
PR8 0.5 MOI
PR8 5 MOI
PR8 50 MOI
Lec1 CTRL
Lec1 TFRC OE

Supplementary Figure 8

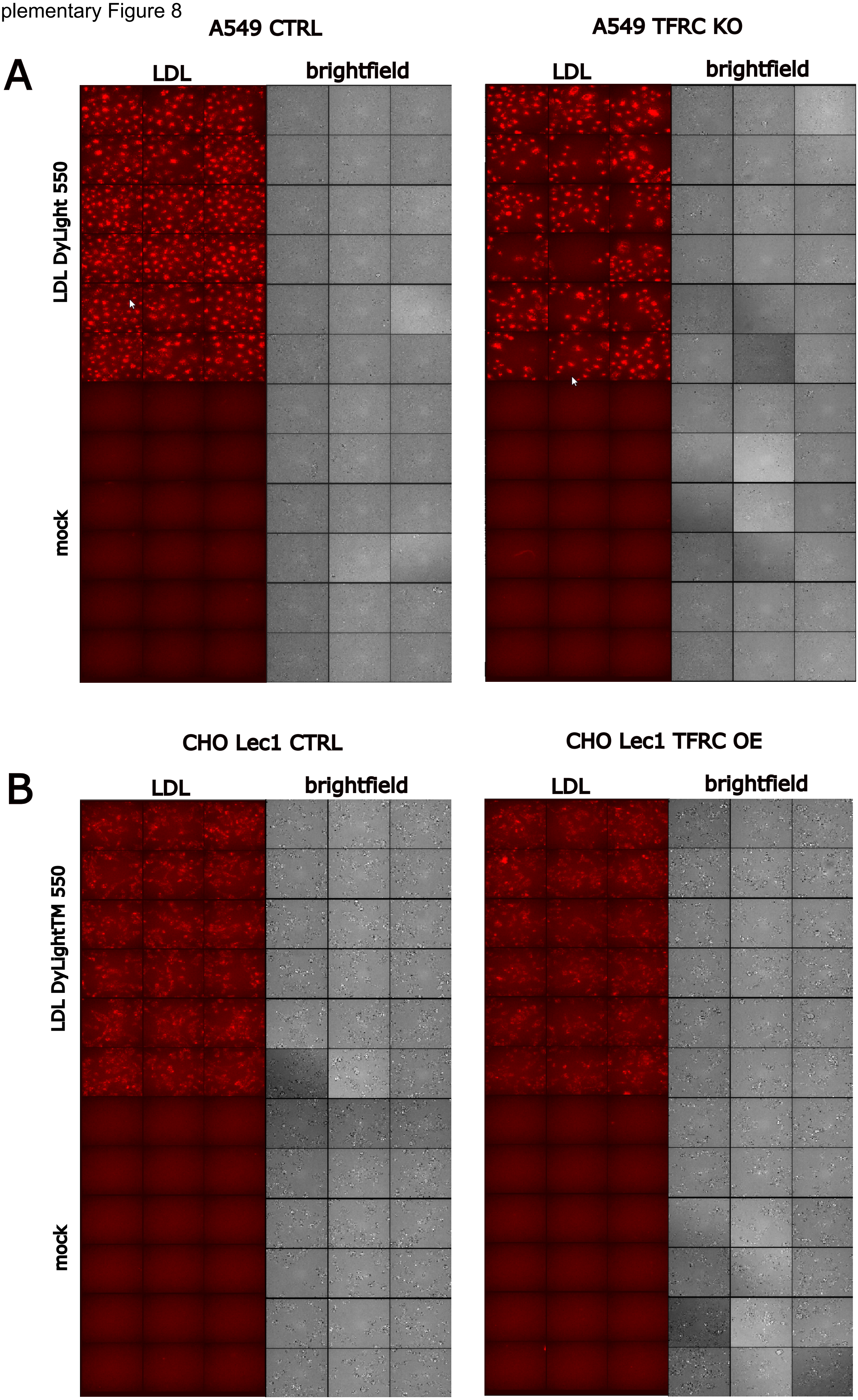

Supplementary Figure 9

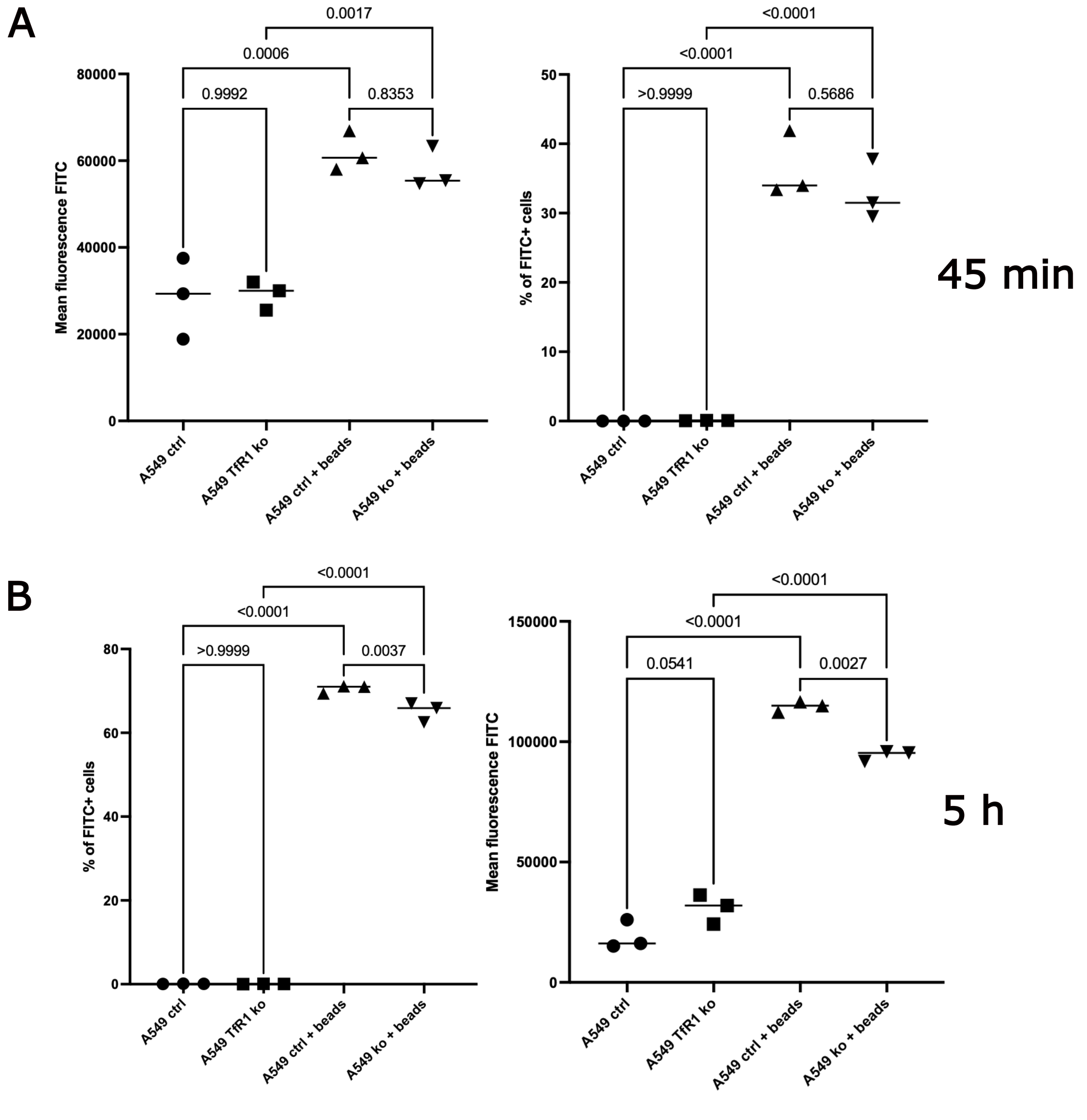

Supplementary Figure 10

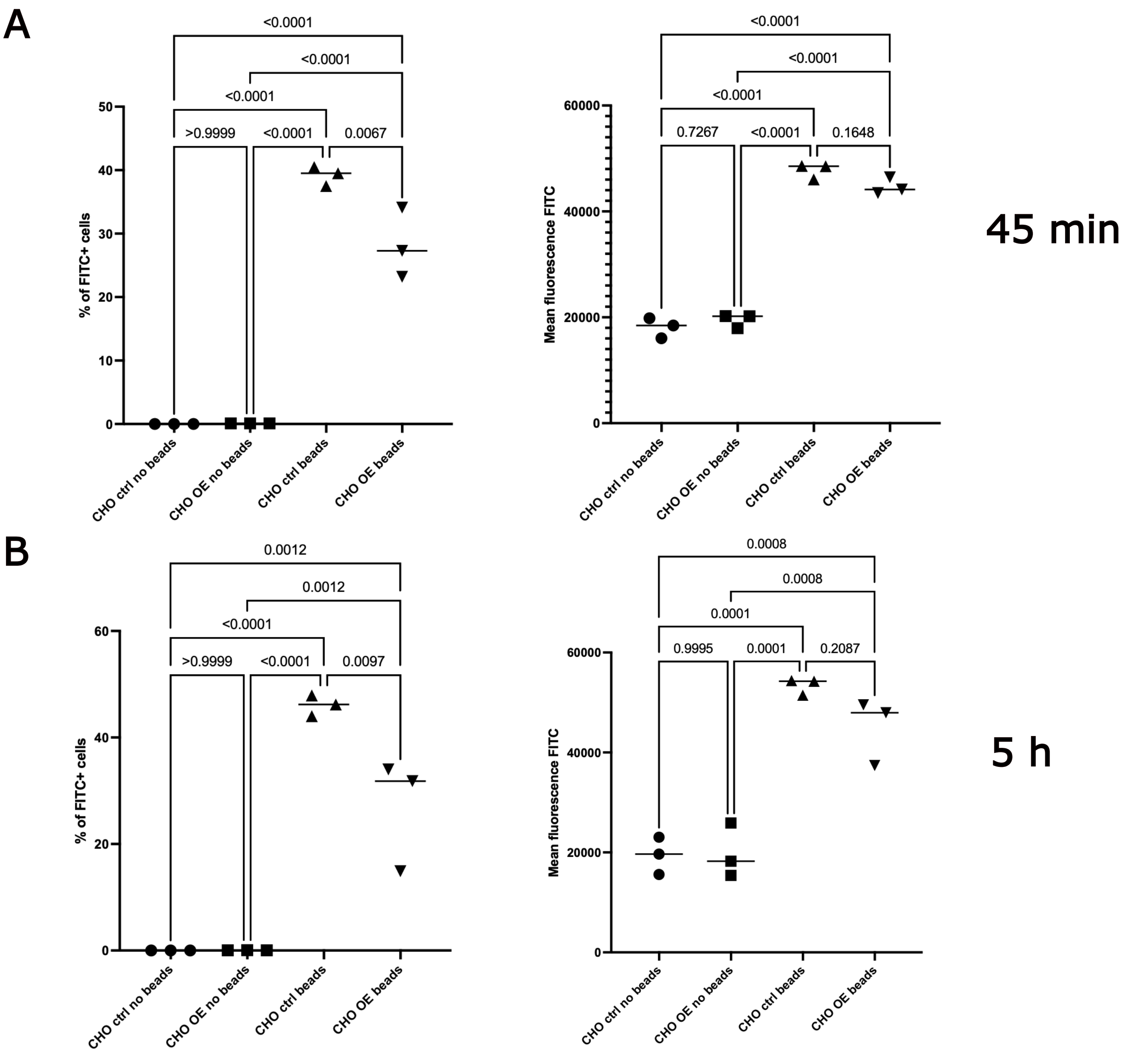

Supplementary Figure 11

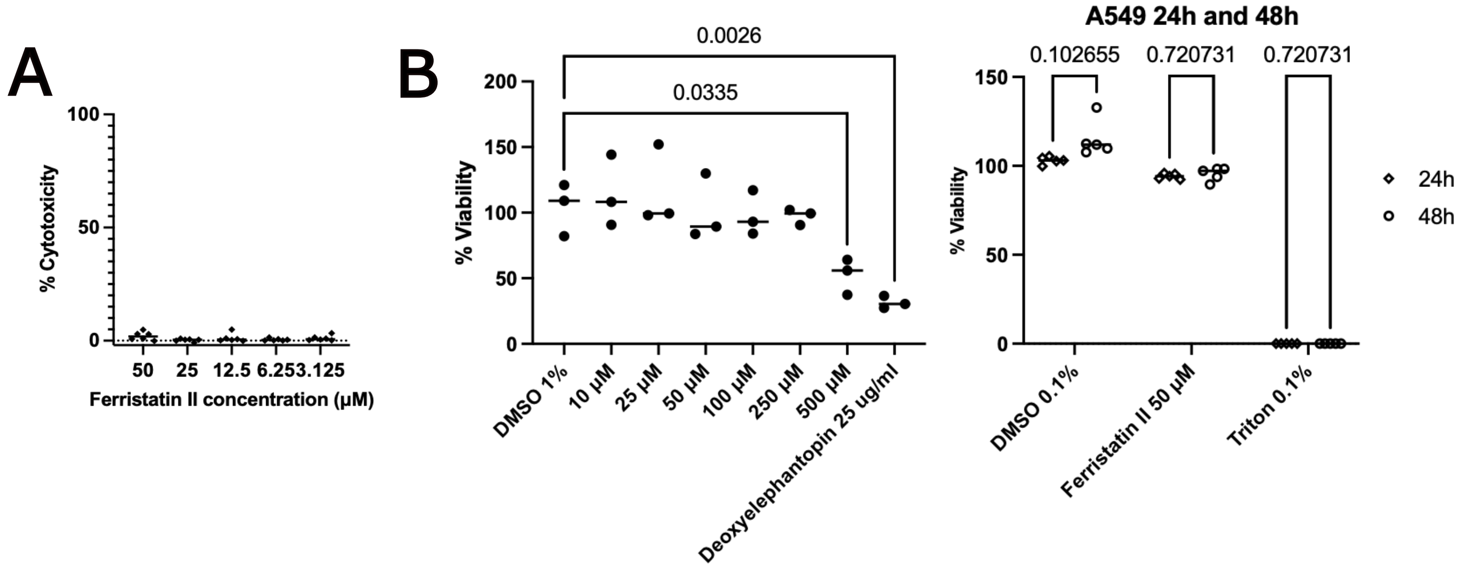

Supplementary Figure 12

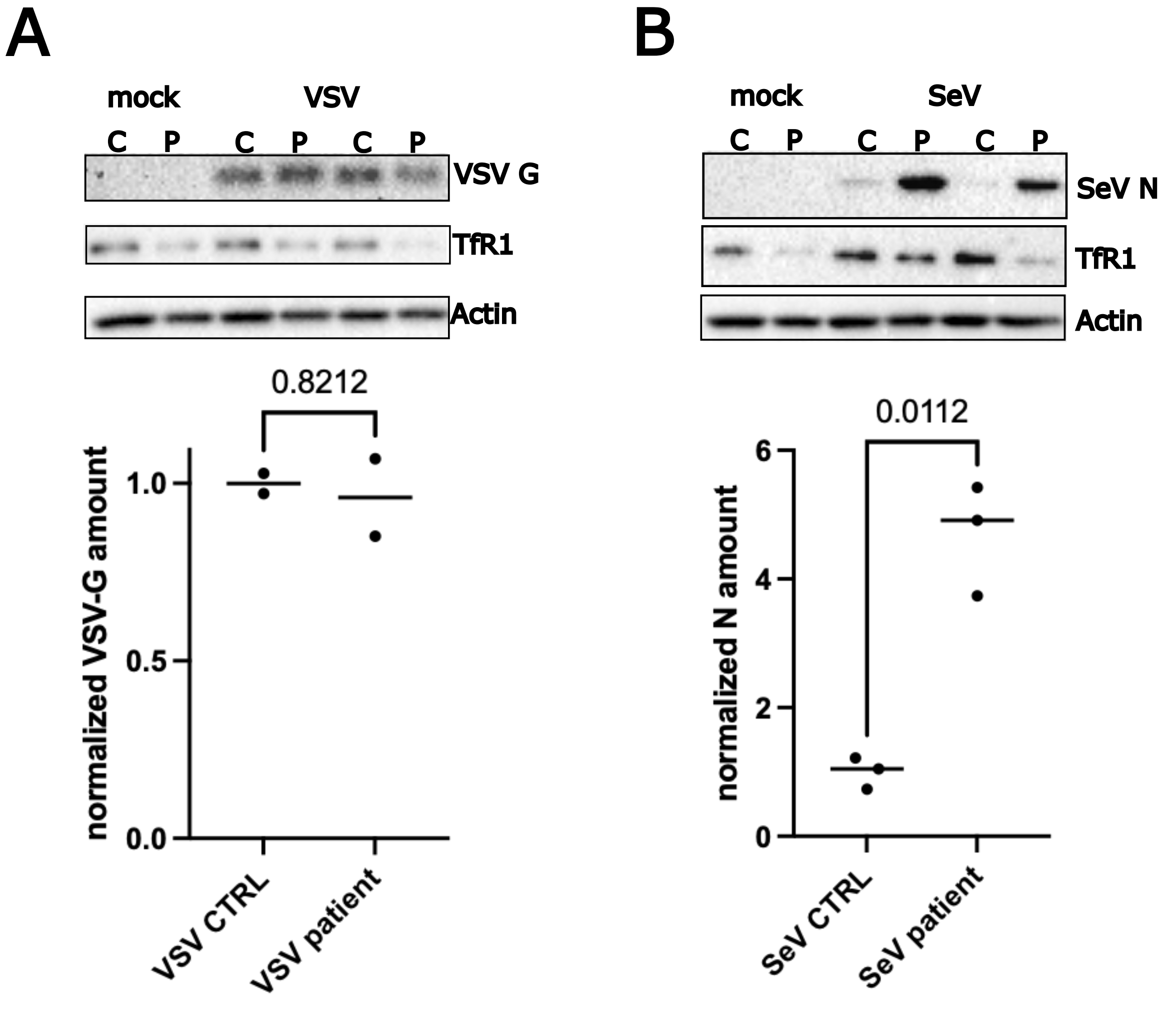

Supplementary Figure 13

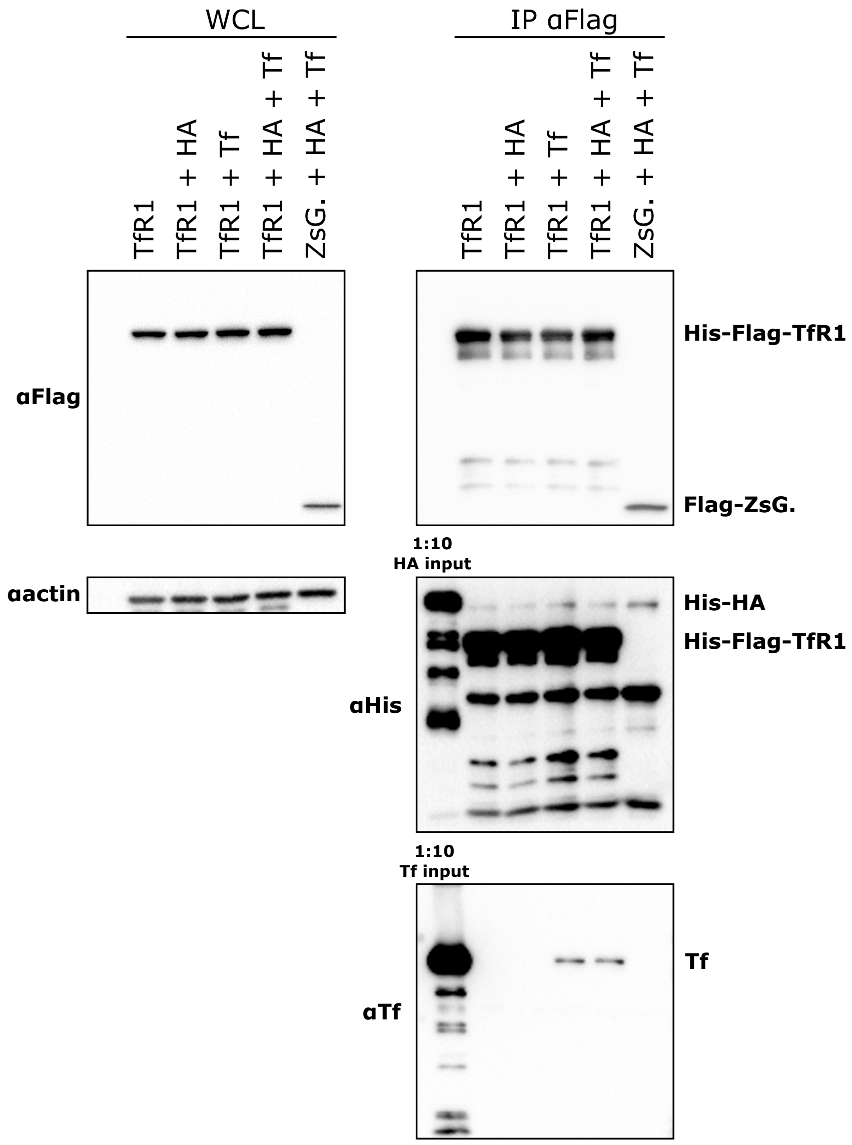

Supplementary Figure 14

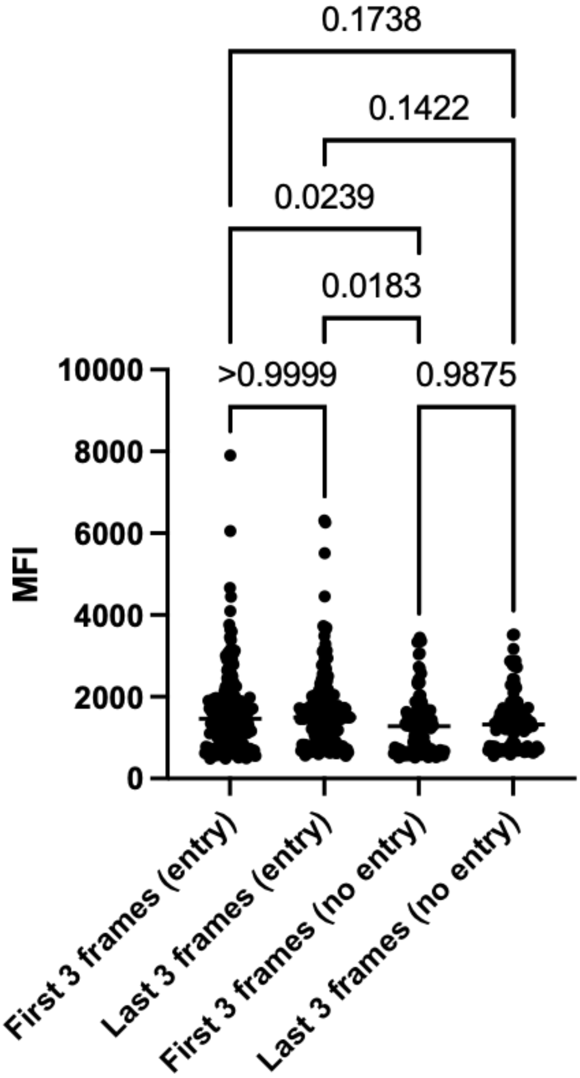

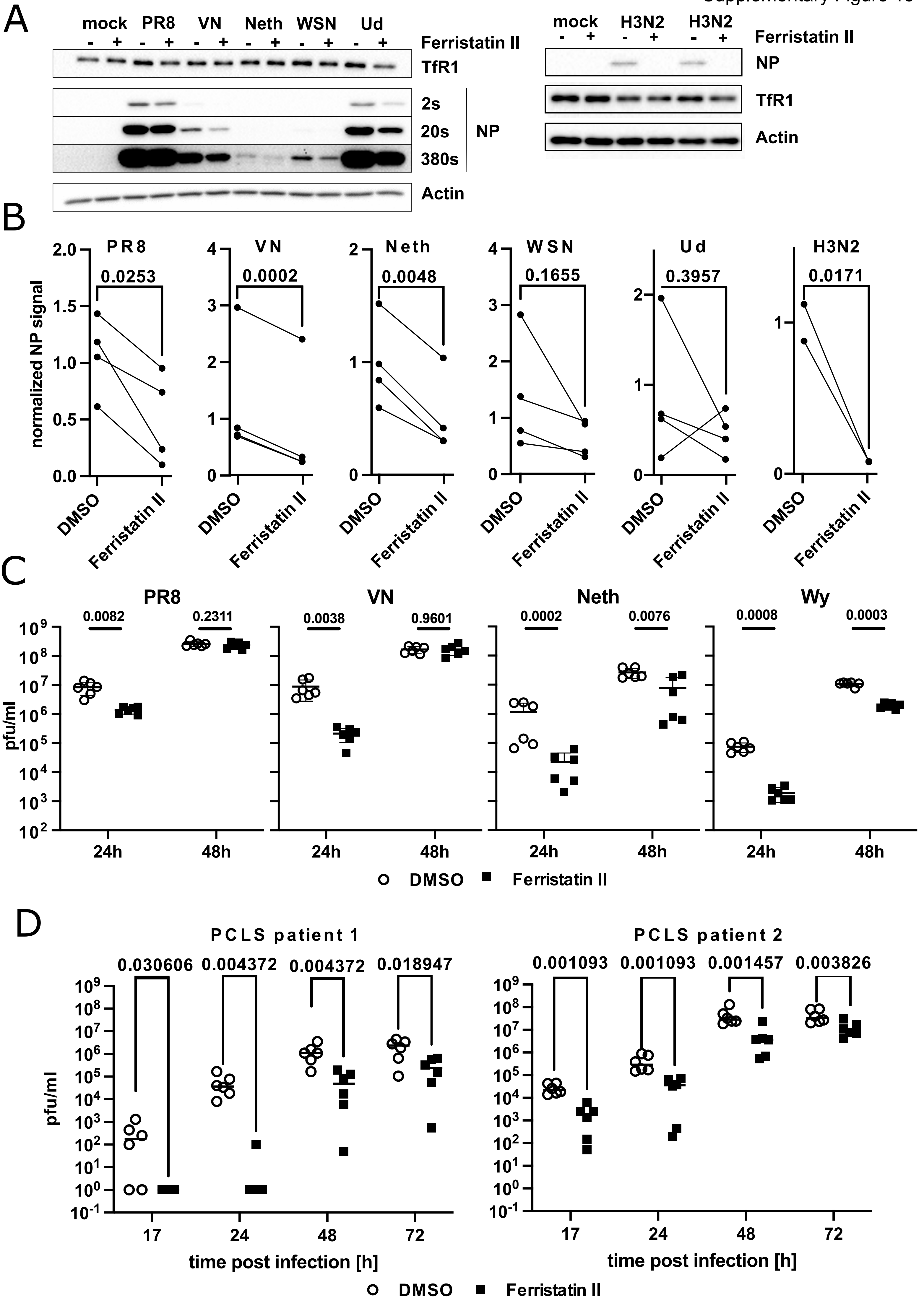

A
mock PR8 VN Neth WSN Ud
Ferristatin II
TfR1
2s
20s
380s
NP
Actin
mock H3N2 H3N2
Ferristatin II
NP
TfR1
Actin
B
PR8 VN Neth WSN Ud H3N2
0.0253
0.0002
0.0048
0.1655
0.3957
0.0171
normalized NP signal
DMSO
Ferristatin II
C
PR8 VN Neth Wy
0.0082
0.2311
0.0038
0.9601
0.0002
0.0076
0.0008
0.0003
pfu/ml
24h
48h
DMSO Ferristatin II
D
PCLS patient 1
PCLS patient 2
0.030606
0.004372
0.004372
0.018947
0.001093
0.001093
0.001457
0.003826
pfu/ml
17
24
48
72
time post infection [h]
DMSO Ferristatin II

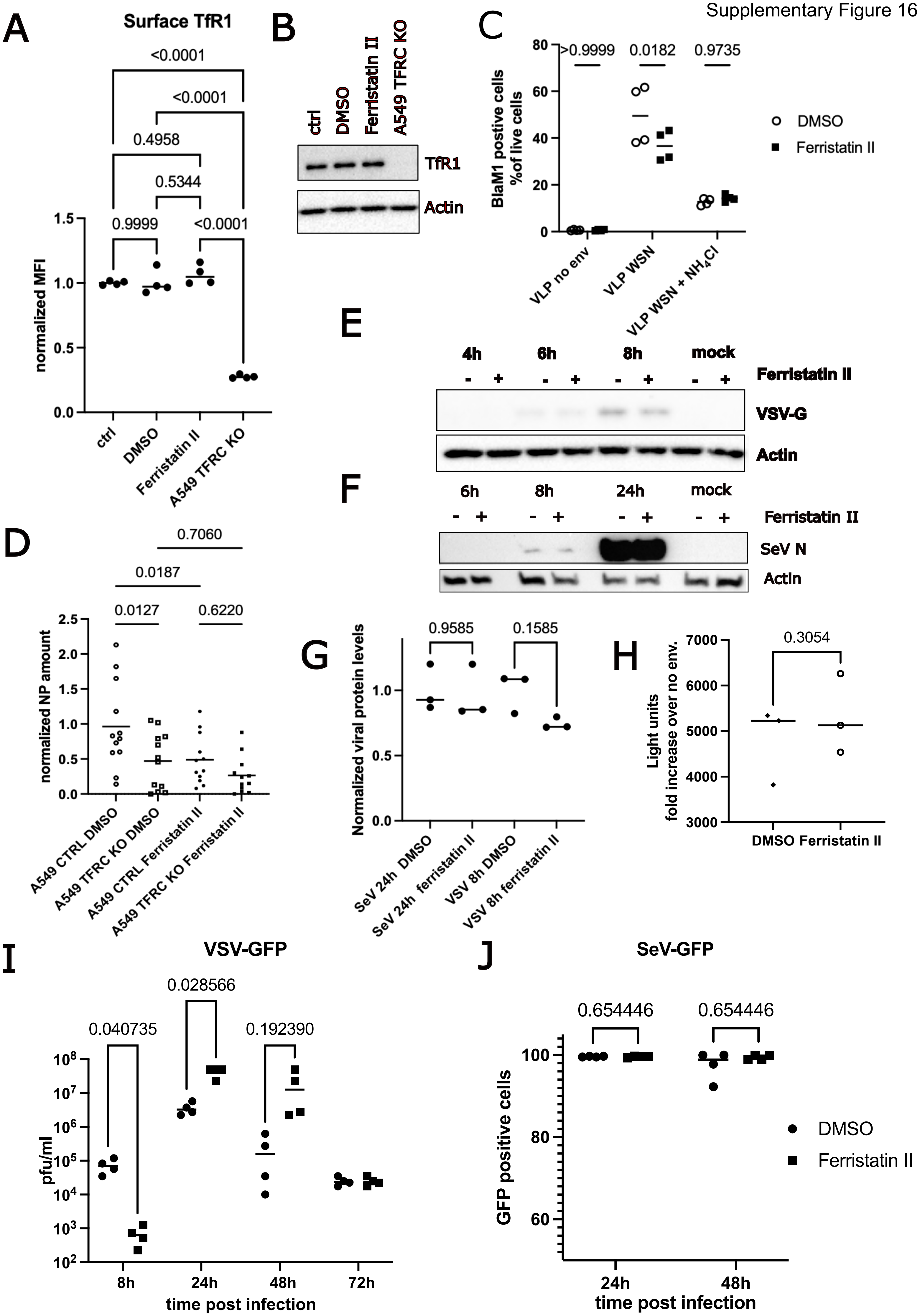
A
Surface TfR1
<0.0001
<0.0001
0.4958
0.5344
0.9999
<0.0001
normalized MFI
ctrl
DMSO
Ferristatin II
A549 TFRC KO
B
ctrl
DMSO
Ferristatin II
A549 TFRC KO
TfR1
Actin
C
>0.9999
0.0182
0.9735
BlaM1 positve cells %of live cells
DMSO
Ferristatin II
VLP no env
VLP WSN
VLP WSN + NH4Cl
D
0.7060
0.0187
0.0127
0.6220
normalized NP amount
A549 CTRL DMSO
A549 TFRC KO DMSO
A549 CTRL Ferristatin II
A549 TFRC KO Ferristatin II
E
4h
6h
8h
mock
Ferristatin II
VSV-G
Actin
F
6h
8h
24h
mock
Ferristatin II
SeV N
Actin
G
0.9585
0.1585
Normalized viral protein levels
SeV 24h DMSO
SeV 24h ferristatin II
VSV 8h DMSO
VSV 8h ferristatin II
H
0.3054
Light units fold increase over no env.
DMSO
Ferristatin II
I
VSV-GFP
0.040735
0.028566
0.192390
pfu/ml
8h
24h
48h
72h
time post infection
J
SeV-GFP
0.654446
0.654446
GFP positive cells
DMSO
Ferristatin II
24h
48h
time post infection

Supplementary Figure 17

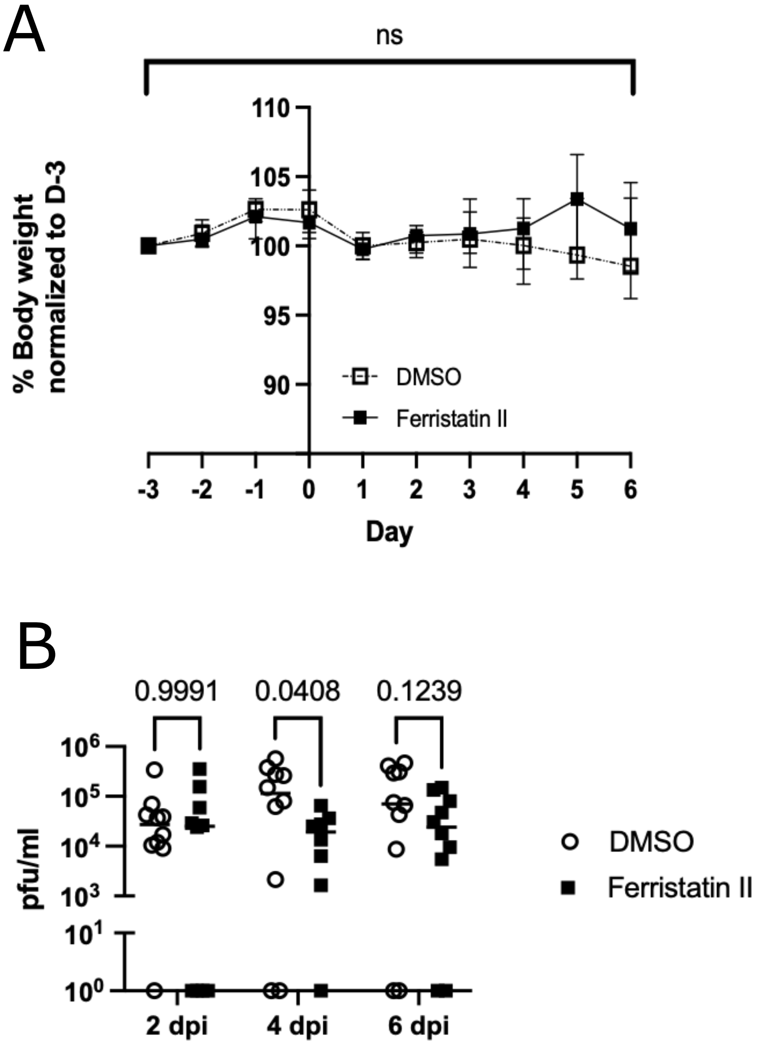

Supplementary Figure 18

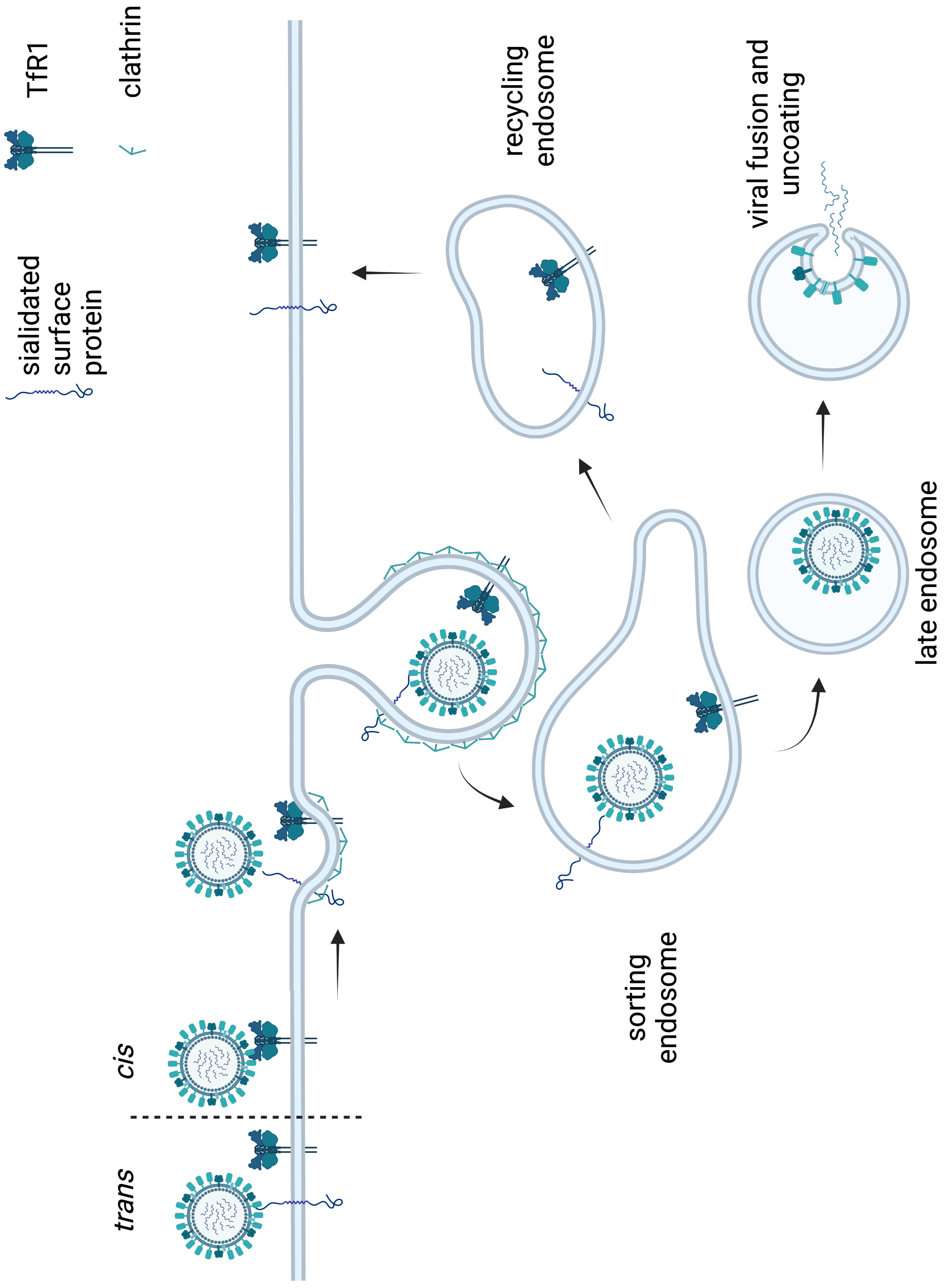

Supplementary Figure 19

Gating strategy (example) for BlaM1 assay analysis (Pro5 CTRL):

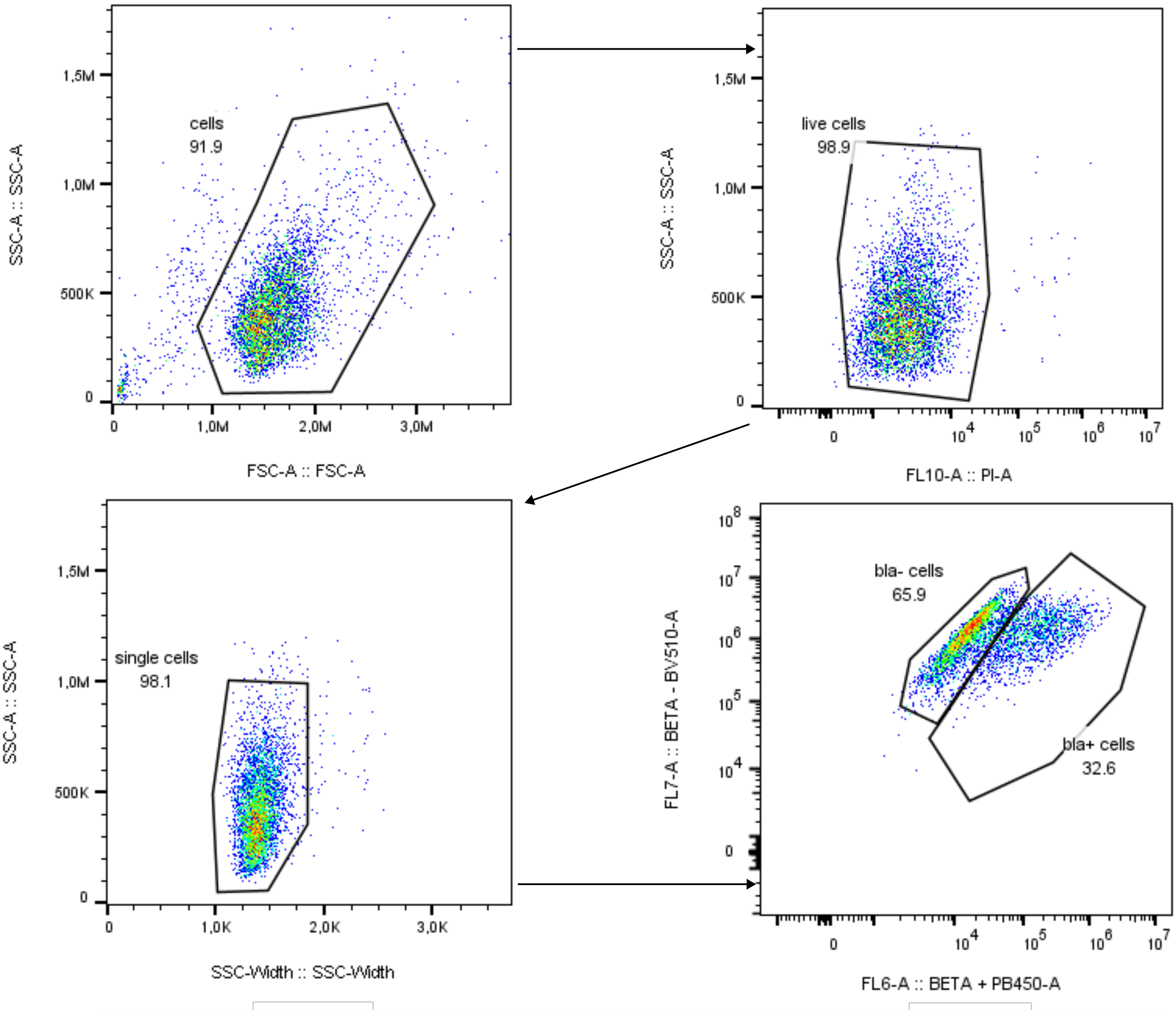

Supplementary Figure 20

Gating strategy (example) for cell surface staining of TfR1

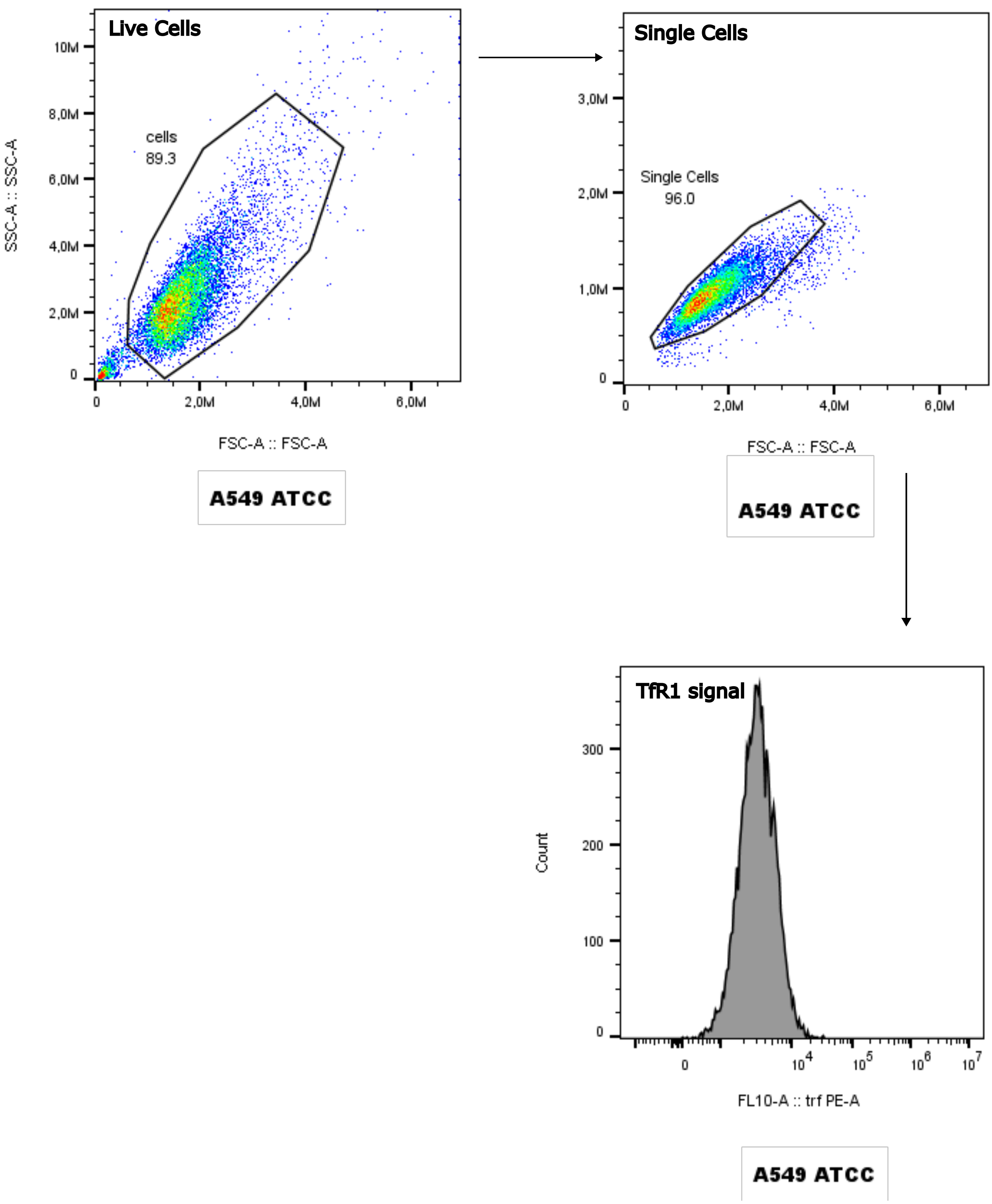

Supplementary Figure 21

A

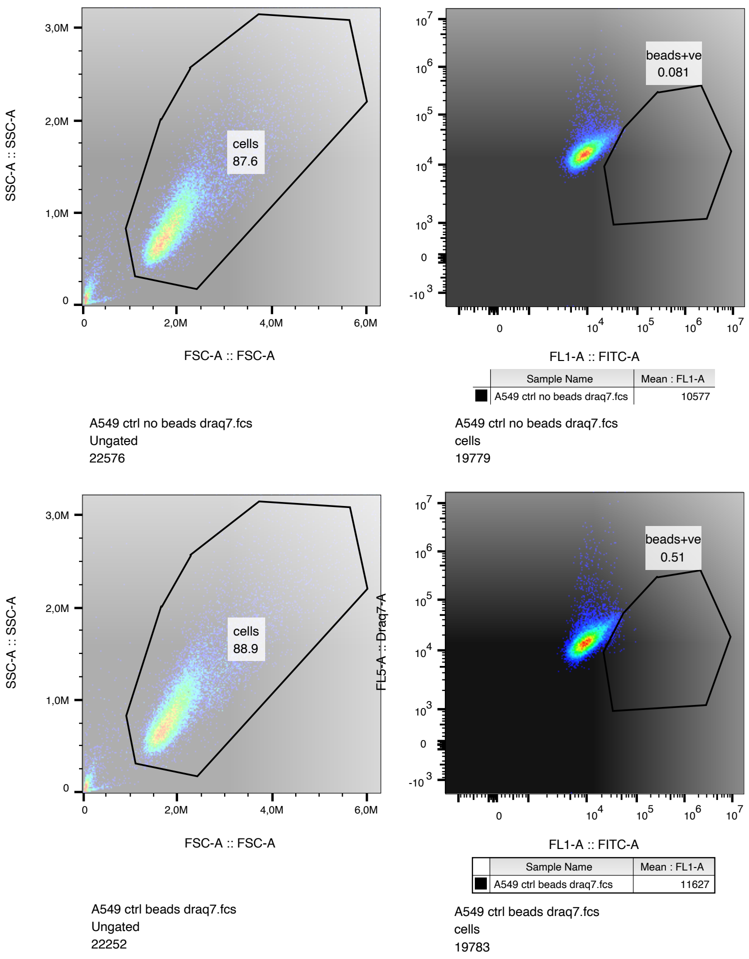

B

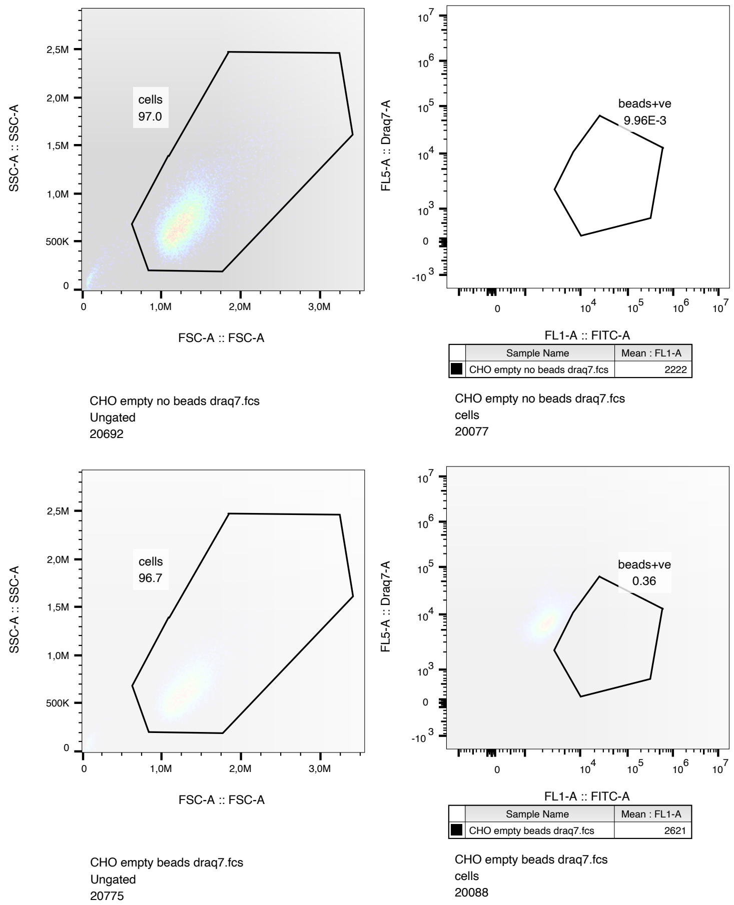

Supplementary Figure 22

Sequence name: hrp

Sequence type: DNA

Biosafety level: Level 1

Express cloning vector / RS: pFastBac1, BamHI/EcoRI

TSE free: No

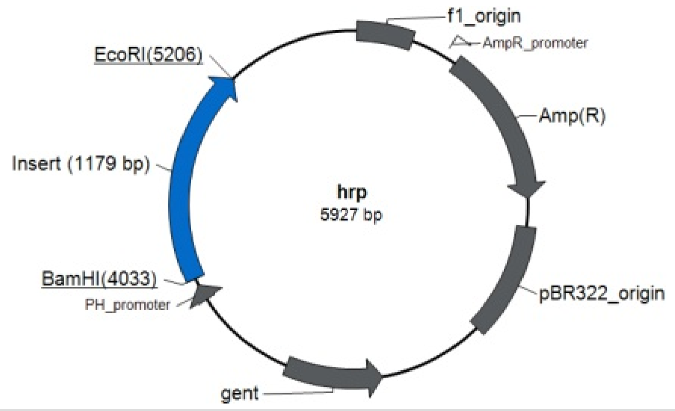

Sequence name / optimized for

hrp/ Non optimized

ORF	Protected sites	Protected areas	Motifs to avoid
7-1173 [ATG...TAA]	1-6 BamHI [GGATCC]<br>1174-1179 EcoRI [GAATTC]		BamHI [GGATCC]<br>EcoRI [GAATTC]

```
              M  K  A  I  L  V  V  L  L  Y  T  F  A  T  A  N  R  L  V  P  R
1.     GGATCCATGAAGGCGATTTTGGTTGTGTTGCTCTACACTTTCGCTACTGCCAACCGCTTGGTCCCACGT
        G  S  P  G  S  G  Y  I  P  E  A  P  R  D  G  Q  A  Y  V  R  K  D  G
70.    GGCTCACCCGGATCTGGATACATCCCGGAGGCCCCTAGGGACGGTCAAGCTTACGTGAGAAAGGACGGC
        E  W  V  L  L  S  T  F  L  G  G  S  G  S  G  S  G  M  Q  L  T  P  T
139.   GAATGGGTTCTGCTGTCGACCTTCTTGGGAGGATCAGGTTCTGGATCAGGTATGCAGTTAACCCCTACA
        F  Y  D  N  S  C  P  N  V  S  N  I  V  R  D  T  I  V  N  E  L  R  S
208.   TTCTACGACAATAGCTGTCCCAACGTGTCCAACATCGTTCGCGACACAATCGTCAACGAGCTCAGATCC
        D  P  R  I  A  A  S  I  L  R  L  H  F  H  D  C  F  V  N  G  C  D  A
277.   GATCCCAGGATCGCTGCTTCAATATTACGTCTGCACTTCCATGACTGCTTCGTGAATGGTTGCGACGCT
        S  I  L  L  D  N  T  T  S  F  R  T  E  K  D  A  F  G  N  A  N  S  A
346.   AGCATATTACTGGACAACACCACCAGTTTCCGCACTGAAAAGGATGCATTCGGGAACGCTAACAGCGCC
        R  G  F  P  V  I  D  R  M  K  A  A  V  E  S  A  C  P  R  T  V  S  C
415.   AGGGGCTTTCCAGTGATCGATCGCATGAAGGCTGCCGTTGAGTCAGCATGCCCACGAACAGTCAGTTGT
        A  D  L  L  T  I  A  A  Q  Q  S  V  T  L  A  G  G  P  S  W  R  V  P
484.   GCAGACCTGCTGACTATAGCTGCGCAACAGAGCGTGACTCTTGCAGGCGGACCGTCCTGGAGAGTGCCG
        L  G  R  R  D  S  L  Q  A  F  L  D  L  A  N  A  N  L  P  A  P  F  F
553.   CTCGGTCGACGTGACTCCCTACAGGCATTCCTAGATCTGGCCAACGCCAACTTGCCTGCTCCATTCTTC
        T  L  P  Q  L  K  D  S  F  R  N  V  G  L  N  R  S  S  D  L  V  A  L
622.   ACCCTGCCCCAGCTGAAGGATAGCTTTAGAAACGTGGGTCTGAATCGCTCGAGTGACCTTGTGGCTCTG
        S  G  G  H  T  F  G  K  N  Q  C  R  F  I  M  D  R  L  Y  N  F  S  N
691.   TCCGGAGGACACACATTTGGAAAGAACCAGTGTAGGTTCATCATGGATAGGCTCTACAATTTCAGCAAC
        T  G  L  P  D  P  T  L  N  T  T  Y  L  Q  T  L  R  G  L  C  P  L  N
760.   ACTGGGTTACCTGACCCCACGCTGAACACTACGTATCTCCAGACACTGAGAGGCTTGTGCCCACTGAAT
        G  N  L  S  A  L  V  D  F  D  L  R  T  P  T  I  F  D  N  K  Y  Y  V
829.   GGCAACCTCAGTGCACTAGTGGACTTTGATCTGCGGACCCCAACCATCTTCGATAACAAGTACTATGTG
        N  L  E  E  Q  K  G  L  I  Q  S  D  Q  E  L  F  S  S  P  N  A  T  D
898.   AATCTAGAGGAGCAGAAAGGCCTGATACAGAGTGATCAAGAACTGTTTAGCAGTCCAAACGCCACTGAC
        T  I  P  L  V  R  S  F  A  N  S  T  Q  T  F  F  N  A  F  V  E  A  M
967.   ACCATCCCACTGGTGAGAAGTTTTGCTAACTCTACTCAAACCTTCTTTAACGCCTTCGTGGAAGCCATG
        D  R  M  G  N  I  T  P  L  T  G  T  Q  G  Q  I  R  L  N  C  R  V  V
1036.  GACCGTATGGGTAACATTACCCCTCTGACGGGTACCCAAGGCCAGATTCGTCTGAACTGCAGAGTGGTC
        N  S  N  S  G  S  G  S  G  S  G  H  H  H  H  H  H  H  H  H  H  *  *
1105.  AACAGCAACTCTGGATCAGGTTCTGGATCAGGTCATCATCATCATCATCACCATCATCATCACTAATAA
1174.  GAATTC
```

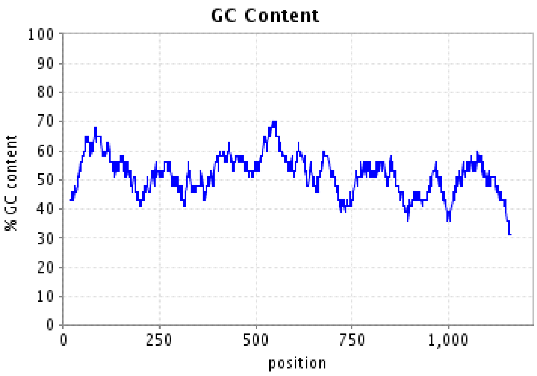

The plots show the GC content in a 40 bp window centered at the indicated nucleotide position.

Supplementary Figure 23

Sequence name: Cal9HAhrp

Sequence type: DNA

Biosafety level: Level 1

Express cloning vector / RS: pFastBac1, BamHI/EcoRI

TSE free: No

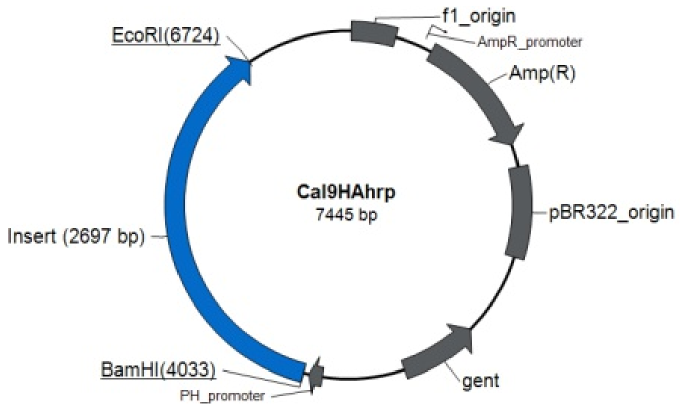

Sequence name / optimized for

Cal9HAhrp/ Non optimized

ORF	Protected sites	Protected areas	Motifs to avoid
7-2691 [ATG...TAA]	1-6 BamHI [GGATCC]<br>2692-2697 EcoRI [GAATTC]		BamHI [GGATCC]<br>EcoRI [GAATTC]

```
         M  K  A  I  L  V  V  L  L  Y  T  F  A  T  A  N  A  D  T  L  C
1.    GGATCCATGAAGGCGATTTTGGTTGTGTTGCTCTACACTTTCGCTACTGCCAACGCCGATACTCTCTGT
      I  G  Y  H  A  N  N  S  T  D  T  V  D  T  V  L  E  K  N  V  T  V  T
70.   ATTGGATACCACGCCAACAACTCTACCGACACTGTGGACACTGTTCTGGAGAAAAACGTGACTGTTACC
      H  S  V  N  L  L  E  D  K  H  N  G  K  L  C  K  L  R  G  V  A  P  L
139.  CACTCTGTAAACCTGCTGGAGGACAAACACAACGGTAAACTGTGTAAACTGAGGGGAGTCGCTCCACTC
      H  L  G  K  C  N  I  A  G  W  I  L  G  N  P  E  C  E  S  L  S  T  A
208.  CATTTGGGAAAATGTAACATTGCCGGCTGGATCTTGGGAAACCCTGAGTGTGAATCCCTGTCGACTGCT
      S  S  W  S  Y  I  V  E  T  P  S  S  D  N  G  T  C  Y  P  G  D  F  I
277.  TCTTCCTGGTCCTACATCGTCGAAACTCCGTCTAGTGATAACGGAACGTGCTACCCTGGTGATTTCATC
      D  Y  E  E  L  R  E  Q  L  S  S  V  S  S  F  E  R  F  E  I  F  P  K
346.  GACTACGAGGAGCTGAGGGAACAACTGTCATCCGTGTCATCATTCGAGCGCTTCGAGATCTTCCCGAAA
      T  S  S  W  P  N  H  D  S  N  K  G  V  T  A  A  C  P  H  A  G  A  K
415.  ACCTCTTCTTGGCCAAACCACGATTCGAACAAGGGAGTAACCGCTGCTTGCCCACATGCTGGTGCCAAA
      S  F  Y  K  N  L  I  W  L  V  K  K  G  N  S  Y  P  K  L  S  K  S  Y
484.  TCCTTCTACAAAAACCTCATCTGGCTGGTGAAAAAGGGAAACTCCTACCCGAAACTCTCCAAATCGTAC
      I  N  D  K  G  K  E  V  L  V  L  W  G  I  H  H  P  S  T  S  A  D  Q
553.  ATCAACGACAAGGGAAAGGAAGTGCTGGTTCTCTGGGGAATCCATCATCCTAGTACTAGTGCCGATCAG
      Q  S  L  Y  Q  N  A  D  T  Y  V  F  V  G  S  S  R  Y  S  K  K  F  K
622.  CAATCTCTCTACCAAAACGCCGACACTTACGTGTTCGTCGGTTCCTCTCGCTACTCTAAAAAGTTCAAA
      P  E  I  A  I  R  P  K  V  R  D  Q  E  G  R  M  N  Y  Y  W  T  L  V
691.  CCGGAGATCGCTATTCGTCCTAAAGTTCGTGATCAAGAAGGTAGAATGAACTACTACTGGACCTTGGTG
      E  P  G  D  K  I  T  F  E  A  T  G  N  L  V  V  P  R  Y  A  F  A  M
760.  GAACCTGGTGACAAAATCACATTCGAGGCTACCGGAAACCTGGTTGTACCACGCTACGCTTTCGCCATG
      E  R  N  A  G  S  G  I  I  I  S  D  T  P  V  H  D  C  N  T  T  C  Q
829.  GAACGTAACGCTGGTAGTGGCATCATCATCTCGGACACACCTGTTCATGATTGTAACACCACATGCCAA
      T  P  K  G  A  I  N  T  S  L  P  F  Q  N  I  H  P  I  T  I  G  K  C
898.  ACACCAAAGGGCGCTATCAACACTTCACTGCCCTTCCAAAACATCCACCCTATCACAATTGGCAAATGC
      P  K  Y  V  K  S  T  K  L  R  L  A  T  G  L  R  N  I  P  S  I  Q  S
967.  CCGAAATACGTCAAATCGACCAAACTGAGACTGGCTACTGGCTTGAGAAACATTCCTAGCATTCAATCC
      R  G  L  F  G  A  I  A  G  F  I  E  G  G  W  T  G  M  V  D  G  W  Y
1036. CGTGGCCTGTTCGGTGCTATCGCTGGCTTCATTGAGGGCGGCTGGACTGGAATGGTGGACGGTTGGTAC
      G  Y  H  H  Q  N  E  Q  G  S  G  Y  A  A  D  L  K  S  T  Q  N  A  I
1105. GGATACCATCATCAAAACGAGCAGGGCTCTGGTTACGCCGCCGACTTGAAATCCACTCAAAACGCCATC
      D  E  I  T  N  K  V  N  S  V  I  E  K  M  N  T  Q  F  T  A  V  G  K
1174. GACGAGATCACAAACAAAGTGAACTCGGTGATCGAAAAAATGAACACCCAATTCACCGCCGTTGGTAAA
      E  F  N  H  L  E  K  R  I  E  N  L  N  K  K  V  D  D  G  F  L  D  I
```

```
     [illegible]
1243. GAGTTCAACCACCTCGAGAAACGTATCGAAAACCTGAACAAAAAAGTGGACGACGGTTTCCTGGACATT
      W  T  Y  N  A  E  L  L  V  L  L  E  N  E  R  T  L  D  Y  H  D  S  N
1312. TGGACCTACAACGCTGAACTCTTGGTGCTGTTGGAAAACGAGCGTACTCTGGATTACCATGACTCCAAC
      V  K  N  L  Y  E  K  V  R  S  Q  L  K  N  N  A  K  E  I  G  N  G  C
1381. GTGAAAAACCTCTACGAGAAAGTCCGTTCACAACTGAAAAACAACGCCAAGGAAATCGGTAACGGTTGC
      F  E  F  Y  H  K  C  D  N  T  C  M  E  S  V  K  N  G  T  Y  D  Y  P
1450. TTCGAGTTCTACCACAAATGTGACAACACGTGTATGGAGTCCGTCAAAAACGGAACTTACGACTACCCG
      K  Y  S  E  E  A  K  L  N  R  E  E  I  D  G  V  S  G  R  L  V  P  R
1519. AAATACTCCGAGGAAGCCAAGCTCAACCGCGAGGAGATCGACGGAGTCAGCGGCCGCTTGGTCCCACGT
      G  S  P  G  S  G  Y  I  P  E  A  P  R  D  G  Q  A  Y  V  R  K  D  G
1588. GGCTCACCCGGATCTGGATACATCCCGGAGGCCCCTAGGGACGGTCAAGCTTACGTGAGAAAGGACGGC
      E  W  V  L  L  S  T  F  L  G  G  S  G  S  G  S  G  M  Q  L  T  P  T
1657. GAATGGGTTCTGCTGTCGACCTTCTTGGGAGGATCAGGTTCTGGATCAGGTATGCAGTTAACCCCTACA
      F  Y  D  N  S  C  P  N  V  S  N  I  V  R  D  T  I  V  N  E  L  R  S
1726. TTCTACGACAATAGCTGTCCCAACGTGTCCAACATCGTTCGCGACACAATCGTCAACGAGCTCAGATCC
      D  P  R  I  A  A  S  I  L  R  L  H  F  H  D  C  F  V  N  G  C  D  A
1795. GATCCCAGGATCGCTGCTTCAATATTACGTCTGCACTTCCATGACTGCTTCGTGAATGGTTGCGACGCT
      S  I  L  L  D  N  T  T  S  F  R  T  E  K  D  A  F  G  N  A  N  S  A
1864. AGCATATTACTGGACAACACCACCAGTTTCCGCACTGAAAAGGATGCATTCGGGAACGCTAACAGCGCC
      R  G  F  P  V  I  D  R  M  K  A  A  V  E  S  A  C  P  R  T  V  S  C
1933. AGGGGCTTTCCAGTGATCGATCGCATGAAGGCTGCCGTTGAGTCAGCATGCCCACGAACAGTCAGTTGT
      A  D  L  L  T  I  A  A  Q  Q  S  V  T  L  A  G  G  P  S  W  R  V  P
2002. GCAGACCTGCTGACTATAGCTGCGCAACAGAGCGTGACTCTTGCAGGCGGACCGTCCTGGAGAGTGCCG
      L  G  R  R  D  S  L  Q  A  F  L  D  L  A  N  A  N  L  P  A  P  F  F
2071. CTCGGTCGACGTGACTCCCTACAGGCATTCCTAGATCTGGCCAACGCCAACTTGCCTGCTCCATTCTTC
      T  L  P  Q  L  K  D  S  F  R  N  V  G  L  N  R  S  S  D  L  V  A  L
2140. ACCCTGCCCCAGCTGAAGGATAGCTTTAGAAACGTGGGTCTGAATCGCTCGAGTGACCTTGTGGCTCTG
      S  G  G  H  T  F  G  K  N  Q  C  R  F  I  M  D  R  L  Y  N  F  S  N
2209. TCCGGAGGACACACATTTGGAAAGAACCAGTGTAGGTTCATCATGGATAGGCTCTACAATTTCAGCAAC
      T  G  L  P  D  P  T  L  N  T  T  Y  L  Q  T  L  R  G  L  C  P  L  N
2278. ACTGGGTTACCTGACCCCACGCTGAACACTACGTATCTCCAGACACTGAGAGGCTTGTGCCCACTGAAT
      G  N  L  S  A  L  V  D  F  D  L  R  T  P  T  I  F  D  N  K  Y  Y  V
2347. GGCAACCTCAGTGCACTAGTGGACTTTGATCTGCGGACCCCAACCATCTTCGATAACAAGTACTATGTG
      N  L  E  E  Q  K  G  L  I  Q  S  D  Q  E  L  F  S  S  P  N  A  T  D
2416. AATCTAGAGGAGCAGAAAGGCCTGATACAGAGTGATCAAGAACTGTTTAGCAGTCCAAACGCCACTGAC
      T  I  P  L  V  R  S  F  A  N  S  T  Q  T  F  F  N  A  F  V  E  A  M
2485. ACCATCCCACTGGTGAGAAGTTTTGCTAACTCTACTCAAACCTTCTTTAACGCCTTCGTGGAAGCCATG
      D  R  M  G  N  I  T  P  L  T  G  T  Q  G  Q  I  R  L  N  C  R  V  V
2554. GACCGTATGGGTAACATTACCCCTCTGACGGGTACCCAAGGCCAGATTCGTCTGAACTGCAGAGTGGTC
      N  S  N  S  G  S  G  S  G  S  G  H  H  H  H  H  H  H  H  H  H  *  *
2623. AACAGCAACTCTGGATCAGGTTCTGGATCAGGTCATCATCATCATCATCACCATCATCATCACTAATAA
2692. GAATTC
```

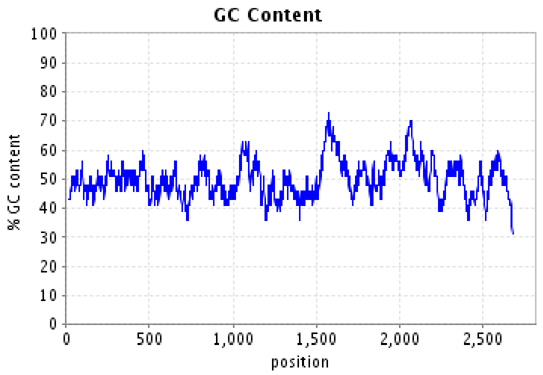

The plots show the GC content in a 40 bp window centered at the indicated nucleotide position.

Supplementary Figure 24

Sequence name: Cal9HAhrpY98F
Sequence type: DNA
Biosafety level: Level 1

Express cloning vector / RS: pFastBac1, BamHI/EcoRI
TSE free: No

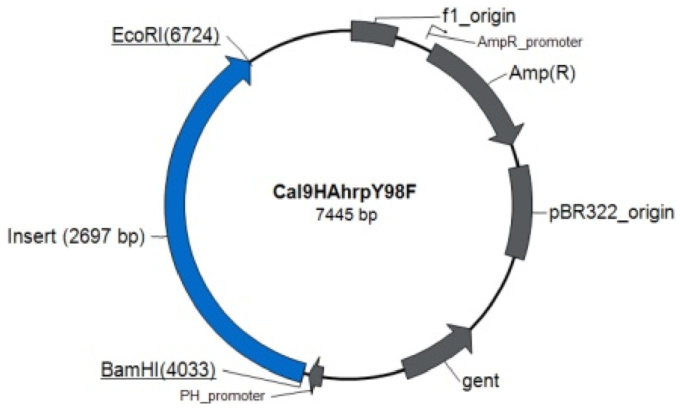

Sequence name / optimized for

Cal9HAhrpY98F/ Non optimized

ORF	Protected sites	Protected areas	Motifs to avoid
7-2691 [ATG...TAA]	1-6 BamHI [GGATCC]<br>2692-2697 EcoRI [GAATTC]		BamHI [GGATCC]<br>EcoRI [GAATTC]

```
         M  K  A  I  L  V  V  L  L  Y  T  F  A  T  A  N  A  D  T  L  C
1.    GGATCCATGAAGGCGATTTTGGTTGTGTTGCTCTACACTTTCGCTACTGCCAACGCCGATACTCTCTGT
      I  G  Y  H  A  N  N  S  T  D  T  V  D  T  V  L  E  K  N  V  T  V  T
70.   ATTGGATACCACGCCAACAACTCTACCGACACTGTGGACACTGTTCTGGAGAAAAACGTGACTGTTACC
      H  S  V  N  L  L  E  D  K  H  N  G  K  L  C  K  L  R  G  V  A  P  L
139.  CACTCTGTAAACCTGCTGGAGGACAAACACAACGGTAAACTGTGTAAACTGAGGGGAGTCGCTCCACTC
      H  L  G  K  C  N  I  A  G  W  I  L  G  N  P  E  C  E  S  L  S  T  A
208.  CATTTGGGAAAATGTAACATTGCCGGCTGGATCTTGGGAAACCCTGAGTGTGAATCCCTGTCGACTGCT
      S  S  W  S  Y  I  V  E  T  P  S  S  D  N  G  T  C  F  P  G  D  F  I
277.  TCTTCCTGGTCCTACATCGTCGAAACTCCGTCTAGTGATAACGGAACGTGCTTCCCTGGTGATTTCATC
      D  Y  E  E  L  R  E  Q  L  S  S  V  S  S  F  E  R  F  E  I  F  P  K
346.  GACTACGAGGAGCTGAGGGAACAACTGTCATCCGTGTCATCATTCGAGCGCTTCGAGATCTTCCCGAAA
      T  S  S  W  P  N  H  D  S  N  K  G  V  T  A  A  C  P  H  A  G  A  K
415.  ACCTCTTCTTGGCCAAACCACGATTCGAACAAGGGAGTAACCGCTGCTTGCCCACATGCTGGTGCCAAA
      S  F  Y  K  N  L  I  W  L  V  K  K  G  N  S  Y  P  K  L  S  K  S  Y
484.  TCCTTCTACAAAAACCTCATCTGGCTGGTGAAAAAGGGAAACTCCTACCCGAAACTCTCCAAATCGTAC
      I  N  D  K  G  K  E  V  L  V  L  W  G  I  H  H  P  S  T  S  A  D  Q
553.  ATCAACGACAAGGGAAAGGAAGTGCTGGTTCTCTGGGGAATCCATCATCCTAGTACTAGTGCCGATCAG
      Q  S  L  Y  Q  N  A  D  T  Y  V  F  V  G  S  S  R  Y  S  K  K  F  K
622.  CAATCTCTCTACCAAAACGCCGACACTTACGTGTTCGTCGGTTCCTCTCGCTACTCTAAAAAGTTCAAA
      P  E  I  A  I  R  P  K  V  R  D  Q  E  G  R  M  N  Y  Y  W  T  L  V
691.  CCGGAGATCGCTATTCGTCCTAAAGTTCGTGATCAAGAAGGTAGAATGAACTACTACTGGACCTTGGTG
      E  P  G  D  K  I  T  F  E  A  T  G  N  L  V  V  P  R  Y  A  F  A  M
760.  GAACCTGGTGACAAAATCACATTCGAGGCTACCGGAAACCTGGTTGTACCACGCTACGCTTTCGCCATG
      E  R  N  A  G  S  G  I  I  I  S  D  T  P  V  H  D  C  N  T  T  C  Q
829.  GAACGTAACGCTGGTAGTGGCATCATCATCTCGGACACACCTGTTCATGATTGTAACACCACATGCCAA
      T  P  K  G  A  I  N  T  S  L  P  F  Q  N  I  H  P  I  T  I  G  K  C
898.  ACACCAAAGGGCGCTATCAACACTTCACTGCCCTTCCAAAACATCCACCCTATCACAATTGGCAAATGC
      P  K  Y  V  K  S  T  K  L  R  L  A  T  G  L  R  N  I  P  S  I  Q  S
967.  CCGAAATACGTCAAATCGACCAAACTGAGACTGGCTACTGGCTTGAGAAACATTCCTAGCATTCAATCC
      R  G  L  F  G  A  I  A  G  F  I  E  G  G  W  T  G  M  V  D  G  W  Y
1036. CGTGGCCTGTTCGGTGCTATCGCTGGCTTCATTGAGGGCGGCTGGACTGGAATGGTGGACGGTTGGTAC
      G  Y  H  H  Q  N  E  Q  G  S  G  Y  A  A  D  L  K  S  T  Q  N  A  I
1105. GGATACCATCATCAAAACGAGCAGGGCTCTGGTTACGCCGCCGACTTGAAATCCACTCAAAACGCCATC
      D  E  I  T  N  K  V  N  S  V  I  E  K  M  N  T  Q  F  T  A  V  G  K
1174. GACGAGATCACAAACAAAGTGAACTCGGTGATCGAAAAAATGAACACCCAATTCACCGCCGTTGGTAAA
      E  F  N  H  L  E  K  R  I  E  N  L  N  K  K  V  D  D  G  F  L  D  I
```

```
        [illegible]
1243.   GAGTTCAACCACCTCGAGAAACGTATCGAAAACCTGAACAAAAAAGTGGACGACGGTTTCCTGGACATT
        W  T  Y  N  A  E  L  L  V  L  L  E  N  E  R  T  L  D  Y  H  D  S  N
1312.   TGGACCTACAACGCTGAACTCTTGGTGCTGTTGGAAAACGAGCGTACTCTGGATTACCATGACTCCAAC
        V  K  N  L  Y  E  K  V  R  S  Q  L  K  N  N  A  K  E  I  G  N  G  C
1381.   GTGAAAAACCTCTACGAGAAAGTCCGTTCACAACTGAAAAACAACGCCAAGGAAATCGGTAACGGTTGC
        F  E  F  Y  H  K  C  D  N  T  C  M  E  S  V  K  N  G  T  Y  D  Y  P
1450.   TTCGAGTTCTACCACAAATGTGACAACACGTGTATGGAGTCCGTCAAAAACGGAACTTACGACTACCCG
        K  Y  S  E  E  A  K  L  N  R  E  E  I  D  G  V  S  G  R  L  V  P  R
1519.   AAATACTCCGAGGAAGCCAAGCTCAACCGCGAGGAGATCGACGGAGTCAGCGGCCGCTTGGTCCCACGT
        G  S  P  G  S  G  Y  I  P  E  A  P  R  D  G  Q  A  Y  V  R  K  D  G
1588.   GGCTCACCCGGATCTGGATACATCCCGGAGGCCCCTAGGGACGGTCAAGCTTACGTGAGAAAGGACGGC
        E  W  V  L  L  S  T  F  L  G  G  S  G  S  G  S  G  M  Q  L  T  P  T
1657.   GAATGGGTTCTGCTGTCGACCTTCTTGGGAGGATCAGGTTCTGGATCAGGTATGCAGTTAACCCCTACA
        F  Y  D  N  S  C  P  N  V  S  N  I  V  R  D  T  I  V  N  E  L  R  S
1726.   TTCTACGACAATAGCTGTCCCAACGTGTCCAACATCGTTCGCGACACAATCGTCAACGAGCTCAGATCC
        D  P  R  I  A  A  S  I  L  R  L  H  F  H  D  C  F  V  N  G  C  D  A
1795.   GATCCCAGGATCGCTGCTTCAATATTACGTCTGCACTTCCATGACTGCTTCGTGAATGGTTGCGACGCT
        S  I  L  L  D  N  T  T  S  F  R  T  E  K  D  A  F  G  N  A  N  S  A
1864.   AGCATATTACTGGACAACACCACCAGTTTCCGCACTGAAAAGGATGCATTCGGGAACGCTAACAGCGCC
        R  G  F  P  V  I  D  R  M  K  A  A  V  E  S  A  C  P  R  T  V  S  C
1933.   AGGGGCTTTCCAGTGATCGATCGCATGAAGGCTGCCGTTGAGTCAGCATGCCCACGAACAGTCAGTTGT
        A  D  L  L  T  I  A  A  Q  Q  S  V  T  L  A  G  G  P  S  W  R  V  P
2002.   GCAGACCTGCTGACTATAGCTGCGCAACAGAGCGTGACTCTTGCAGGCGGACCGTCCTGGAGAGTGCCG
        L  G  R  R  D  S  L  Q  A  F  L  D  L  A  N  A  N  L  P  A  P  F  F
2071.   CTCGGTCGACGTGACTCCCTACAGGCATTCCTAGATCTGGCCAACGCCAACTTGCCTGCTCCATTCTTC
        T  L  P  Q  L  K  D  S  F  R  N  V  G  L  N  R  S  S  D  L  V  A  L
2140.   ACCCTGCCCCAGCTGAAGGATAGCTTTAGAAACGTGGGTCTGAATCGCTCGAGTGACCTTGTGGCTCTG
        S  G  G  H  T  F  G  K  N  Q  C  R  F  I  M  D  R  L  Y  N  F  S  N
2209.   TCCGGAGGACACACATTTGGAAAGAACCAGTGTAGGTTCATCATGGATAGGCTCTACAATTTCAGCAAC
        T  G  L  P  D  P  T  L  N  T  T  Y  L  Q  T  L  R  G  L  C  P  L  N
2278.   ACTGGGTTACCTGACCCCACGCTGAACACTACGTATCTCCAGACACTGAGAGGCTTGTGCCCACTGAAT
        G  N  L  S  A  L  V  D  F  D  L  R  T  P  T  I  F  D  N  K  Y  Y  V
2347.   GGCAACCTCAGTGCACTAGTGGACTTTGATCTGCGGACCCCAACCATCTTCGATAACAAGTACTATGTG
        N  L  E  E  Q  K  G  L  I  Q  S  D  Q  E  L  F  S  S  P  N  A  T  D
2416.   AATCTAGAGGAGCAGAAAGGCCTGATACAGAGTGATCAAGAACTGTTTAGCAGTCCAAACGCCACTGAC
        T  I  P  L  V  R  S  F  A  N  S  T  Q  T  F  F  N  A  F  V  E  A  M
2485.   ACCATCCCACTGGTGAGAAGTTTTGCTAACTCTACTCAAACCTTCTTTAACGCCTTCGTGGAAGCCATG
        D  R  M  G  N  I  T  P  L  T  G  T  Q  G  Q  I  R  L  N  C  R  V  V
2554.   GACCGTATGGGTAACATTACCCCTCTGACGGGTACCCAAGGCCAGATTCGTCTGAACTGCAGAGTGGTC
        N  S  N  S  G  S  G  S  G  S  G  H  H  H  H  H  H  H  H  H  H  *  *
2623.   AACAGCAACTCTGGATCAGGTTCTGGATCAGGTCATCATCATCATCATCACCATCATCATCACTAATAA
2692.   GAATTC
```

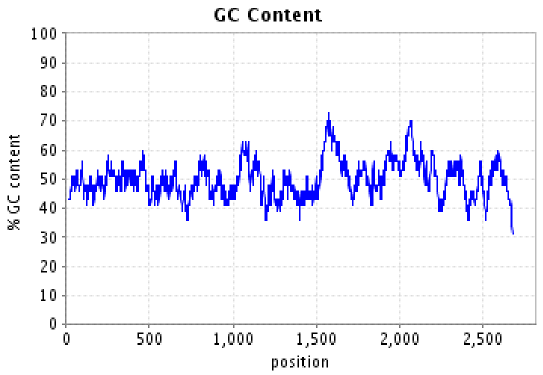

The plots show the GC content in a 40 bp window centered at the indicated nucleotide position.
